# DFT investigation of the mechanism and role of N-heterocyclic carbene (NHC) in constructing asymmetric organosilanes using NHC-catalyzed [4+2] cycloaddition reaction†

**DOI:** 10.1039/d4ra03676j

**Published:** 2024-11-06

**Authors:** Batoul Alipour

**Affiliations:** a Department of Chemistry, Tarbiat Modares University P.O. Box 14115 175 Tehran Iran a.batoul@modares.ac.ir

## Abstract

Herein, the mechanism and origin of stereoselectivity for the asymmetric [4+2] cycloaddition between (*E*)-3-(*p*-tolyl)acrylaldehyde (R1) and phenyl-3-(trimethylsilyl)prop-2-en-1-one (R2) in the presence of an N-heterocyclic carbene (NHC) were theoretically scrutinized. The desirable catalytic cycle is characterized by five steps: (1) the coupling reaction of the NHC catalyst with R1, the formation of the Breslow and enolate intermediates in the second and third steps, (4) the formal [4+2] cycloaddition reaction to form the stereoselective C–C bond, and (5) the regeneration of NHC to obtain asymmetric organosilanes. In the most energetically favorable pathway, the formation of the enolate intermediate exhibits the highest energy barrier of about 19.48 kcal mol^−1^ (*Re*-TS_2_BA) and is the rate-determining step. The [4+2] cycloaddition reaction is the stereoselectivity-determining step forming the chiral C–C bond with *RR*, *RS*, *SR* and *SS* configurations, among which *RS* is the most desirable configuration. The origin of stereoselectivity was investigated using distortion energy analysis. The first and fourth steps helped in investigating the effects of electron-donating (Me) and electron-withdrawing (Cl) groups on cinnamaldehyde. Conceptual DFT (CDFT) analysis was carried out to confirm the critical role of the NHC catalyst as a Lewis base during the reaction processes.

## Abstract 2

The mechanism and origin of stereoselectivity for the synthesis of asymmetric organosilanes using NHC are theoretically investigated.

## Graphical abstract

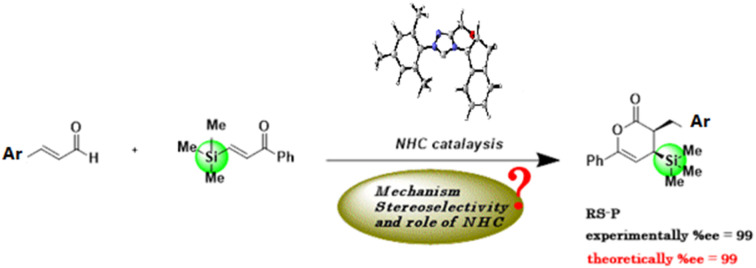

## 1. Introduction

**Scheme 1:**
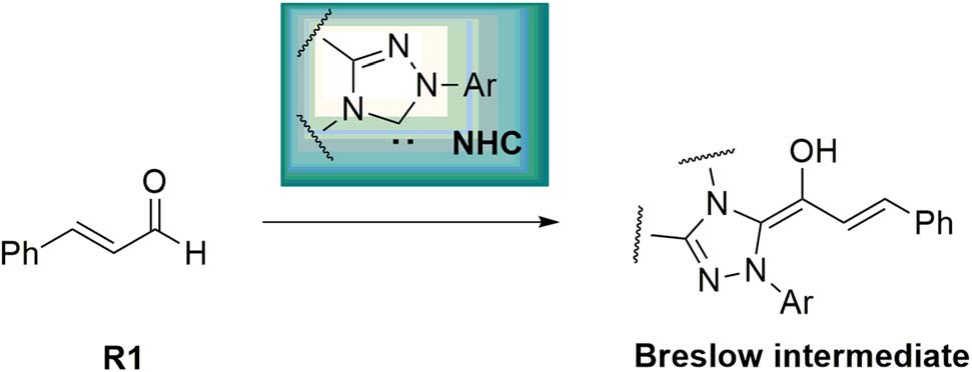
Formation of the Breslow intermediate.

Asymmetric organosilanes have interesting and useful applications in different fields, including organic synthesis,^1^ agroscience,^2^ materials science,^3^ and medicinal chemistry.^4^ Owing to the presence of the silicon atom in their structures, they have a particular physical and electronic nature. Several synthetic strategies have been developed to produce asymmetric organosilanes, which rely on transition metals as catalysts.^5,6^ In contrast, developing organocatalysis-based strategies to construct asymmetric organosilanes has become a great and essential challenge.^7^ N-Heterocyclic carbenes (NHCs) act as key and powerful tools to construct chiral C–X (X = C, N or O) bonds in various asymmetric organic reactions, such as Stetter, cross-benzoin, Mannich, Michael, and cycloaddition reactions.^8–12^ Interestingly, many annulation reactions in the formation of carbocyclic and heterocyclic systems using NHC catalysts have been reported. These reactions have been performed with excellent stereo- and regioselectivity. NHC-catalyzed annulation reactions were carried out with the Umpolung of enals and diverse electrophilic substrates, such as aldehydes, imines, and ketones.^13^ In fact, the complexation of NHCs and enals results in the inversion of natural reactivity (*i.e.*, Umpolung reaction) and forms the Breslow intermediate, which works as a prenucleophile, as shown in Scheme 1.

**Scheme 2:**
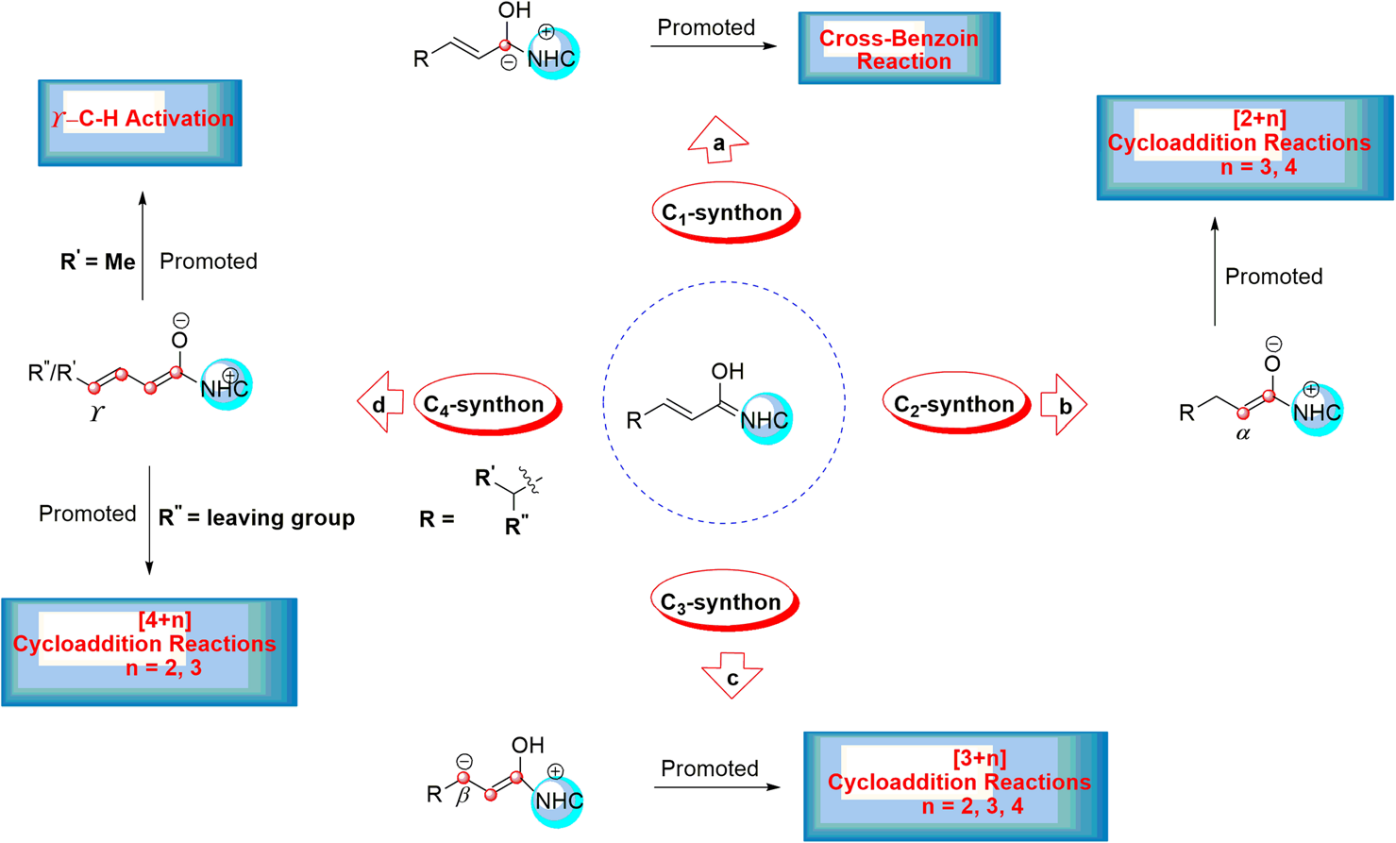
The formation of different reactive intermediates from the Breslow intermediate.

This leads to the formation of various reactive intermediates possessing one, two, three, and four active carbon centers. α-Carbon (enolate intermediate b),^14,15^ β-carbon (homoenolate intermediate c),^16–20^ γ-carbon (dienolate intermediate d),^21–23^ and carbonyl carbon (acyl anion equivalent intermediate a) are among these intermediates (Scheme 2).^24–26^

This intermediate serves as a C_*X*_ (*X* = 1, 2, 3, and 4) carbon synthon in diverse annulation reactions (Scheme 2). For instance, the cross-benzoin reaction occurs when the Breslow intermediate acts as a C_1_-synthon.^24–26^ A C_2_-synthon (enolate intermediate) performs different types of [2+*n*] (*n* = 3, 4) annulation reactions.^27,28^ A C_3_-synthon (homoenolate intermediate) shows different types of [3+*n*] (*n* = 3, 4) annulation reactions.^8^ Finally, the NHC-catalyzed [4+*n*] (*n* = 2, 3) annulation reaction occurs through a C_4_-synthon (dienolate intermediate).^29,30^ Glorius and Bode reported that the NHC-catalyzed reactions between enals and aldehydes resulted in the production of γ-lactones generated by the homoenolate intermediates.^16,17^ These results lead to much attention for the construction of different kinds of compounds through the activated β-carbon in the homoenolates.^31^ Also, Bode suggested that the conjugated Breslow intermediate can undergo the protonation process and form the enol or enolate intermediate.^32^ In addition, Scheidt^33^ and Chi^15^ indicated that the proton transfer of the β-carbon of homoenolate led to the generation of the enolate intermediate under certain experimental conditions.

**Scheme 3:**
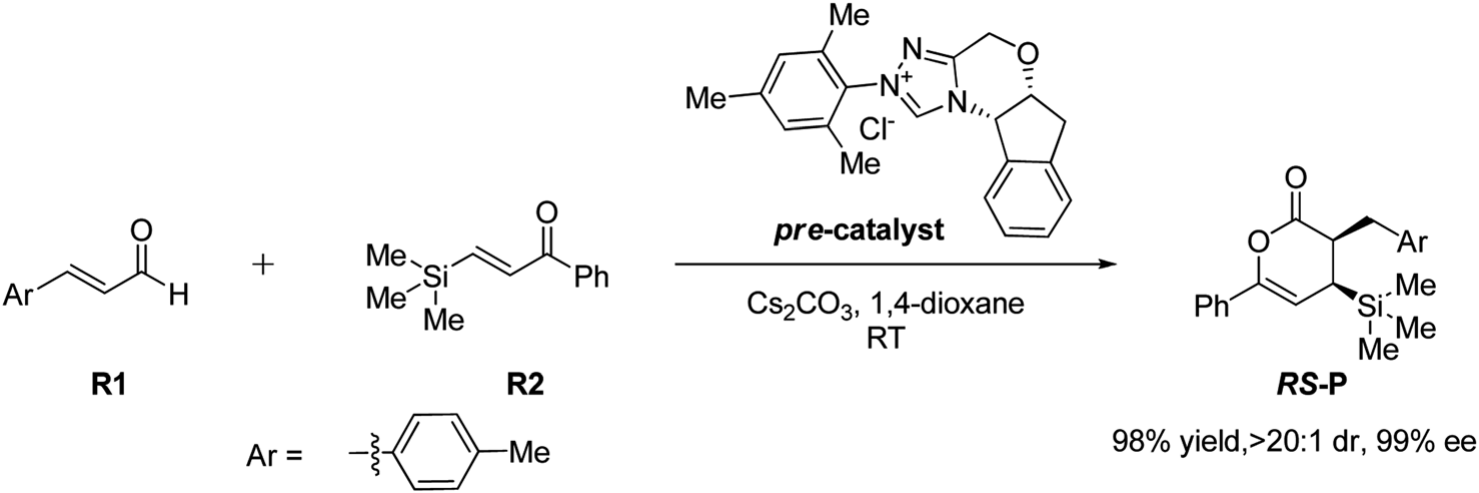
NHC-catalyzed [4+2] annulation reaction of (*E*)-3-(*p*-tolyl)acrylaldehyde (R1) and phenyl-3-(trimethylsilyl)prop-2-en-1-one (R2).

In 2018, Fu and Huang advanced an interesting [4+2] cycloaddition reaction between (*E*)-3-(*p*-tolyl)acrylaldehyde (R1) and phenyl-3-(trimethylsilyl)prop-2-en-1-one (R2).^34^ This reaction was performed in the presence of triazolium salt as a precatalyst and Cs_2_CO_3_ and 1,4-dioxane (DO) as the base and solvent, respectively. This reaction proceeded smoothly at ambient temperature to afford the asymmetric organosilanes in good yields and excellent stereoselectivities (Scheme 3).

Although construction methods of carbon–carbon chiral bonds have been developed, the comprehension of their mechanism continues to be challenging. Over the years, numerous theoretical studies have been published to elucidate the characterization of NHC as a catalyst and the origin of stereoselectivity in annulation reactions.^35–39^ Sunoj and co-workers explored the detailed mechanism of several reactions in NHC-catalyzed [3+2] and [4+2] annulation reactions using DFT calculations.^40^ Wei and co-workers revealed the origin of stereoselectivity in different types of NHC-catalyzed cycloaddition reactions through density functional theory (DFT).^37,41,42^ More theoretical investigations have been reported to elucidate the detailed mechanism of NHC-catalyzed [*n*+2] (*n* = 2, 3, 4) and [3+4] annulation reactions by other groups.^36,43–46^

Herein, DFT computations of the NHC-catalyzed [4+2] cycloaddition reaction between R1 and R2 have been performed, as shown in Scheme 3. This study aims to describe the potential role of N-heterocyclic carbene (NHC) as an organocatalyst in promoting [4+2] cycloaddition reactions with a deep insight and a broad understanding of their important and influential role in organic synthesis, which solved the following questions.

(1) How can the unsaturated sp^2^ carbon of R1 be activated in this reaction? (2) How can proton transfer be performed in this reaction? (3) What is the role of NHC and Cs_2_CO_3_? (4) Which step determines the rate or stereoselectivity? (5) What is the origin of the stereoselectivity?

And (6) what are the effects of substitution on the addition of NHC to R1 and stereoselectivity?

## 2. Computational method

Gaussian 09 software was employed to investigate all DFT calculations.^47^ The reported DFT calculations show that the M06-2X method gives an actual outcome of the kinetic and thermodynamic parameters.^48–55^ The geometries of all structures were investigated using the M06-2X^48,56,57^ method along with the 6-31G(d,p) basis set to optimize the C, H, N, and O atoms. Also, lanl2dz^58,59^ was applied to optimize the Cs atom. The solvent environmental effects were considered in all theoretical calculations in 1,4-dioxane solvent through the integral equation formalism polarizable continuum model (IEF-PCM).^60,61^ The vibrational frequency was computed at the same level to access the corrections in the Gibbs free energies and verify the character of each stationary point. The minima state had no imaginary frequencies (NIMAG = 0), while the transition state (TS) had only one imaginary frequency (NIMAG = 1). Single-point energy for all optimized structures were calculated at the M06-2X/6-311++G(2df,2pd) level in implicit 1,4-dioxane using IEF-PCM. Also, tight convergence criterion and ultrafine integral grid were used to enhance the accuracy of the calculations. In this study, all of the detailed mechanisms were scrutinized using the Gibbs free energies resulting from the addition of the thermal corrections Gibbs free energies at the M06-2X/6-31G(d,p)//(dioxane) IEF/PCM level (L1) to the relevant single point energies at the M06-2X/6-311++G(2df,2pd)//(dioxane) IEF/PCM level (L2). Intrinsic reaction coordinate (IRC)^62,63^ calculations were done at the M06-2X/6-31G(d,p) level to confirm the transition states that led to the desired reactants and products. Natural bond orbital (NBO) analysis^64–66^ was used at the M06-2X/6-31G(d,p) level to calculate the atomic charge distribution. In addition, the CDFT-derived reactivity index was described similarly to the global reactivity index (GRI) analysis,^67^ which was executed to indicate the role of the organocatalyst. In this analysis, the electrophilicity index *ω* was obtained through *ω* = (*μ*^2^/2*η*) (eV), in which *μ* was defined as electronic chemical potential and *η* was defined as chemical hardness.^68,69^ With the use of expressions *μ* ≈ (*E*_H_ − *E*_L_)/2 and *η* ≈ (*E*_L_ − *E*_H_), the values of *μ* and *η* were obtained. The *E*_H_ and *E*_L_ are the one-electron energy of the HOMO and LUMO orbitals in the corresponding molecules, respectively. It is worth mentioning that the nucleophilicity index *N* = *E*_H(Nu)_ − *E*_H(TCNE)_, in which tetracyanoethylene (TCNE) is known as the ref. 70–72. The Parr functions (*P*_k_^+^ and *P*_k_^−^)^73,74^ were assessed to compute both the local electrophilicity and local nucleophilicity indexes, which were obtained through expressions *ω*_k_ (*ω*_k_ = *ωP*_k_^+^)^75^ and *N*_k_ (*N*_k_ = *NP*_k_^−^)^76^ for some key atoms in the reactants and intermediates, respectively. Three-dimensional (3D) structures in this article were performed using the CYL-view software.^77^ In addition, Boltzmann distribution calculation was performed to determine the extent of enantioselectivity in terms of enantiomeric excess (ee%). The value of diastereomeric (ee%) transition states were obtained by the following equations.
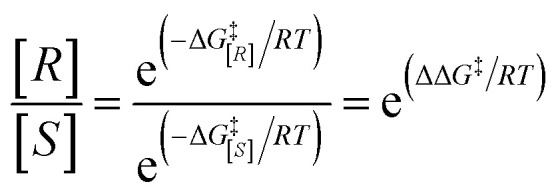

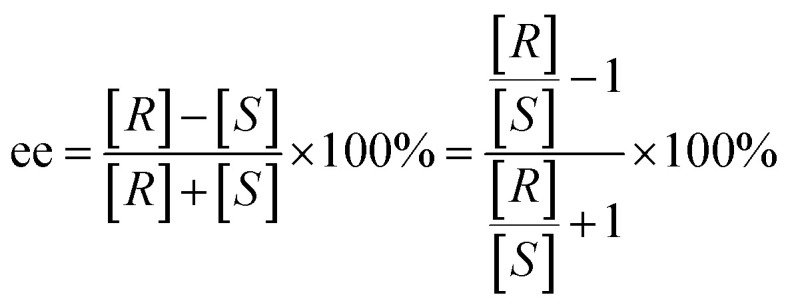
where *G*^‡^ is the Gibbs free energy barrier of the competing diastereomeric transition state and Δ*G*^‡^ is the Gibbs free energy difference between two competing intended diastereomeric transition states.

## 3. Results and discussion

In this study, two possible routes for constructing the asymmetric organosilanes of (*E*)-3-(*p*-tolyl)acrylaldehyde (R1) and phenyl-3-(trimethylsilyl)prop-2-en-1-one (R2) are proposed and investigated, which are discussed in detail as follows.

### 3.1. The first reaction route

**Scheme 4:**
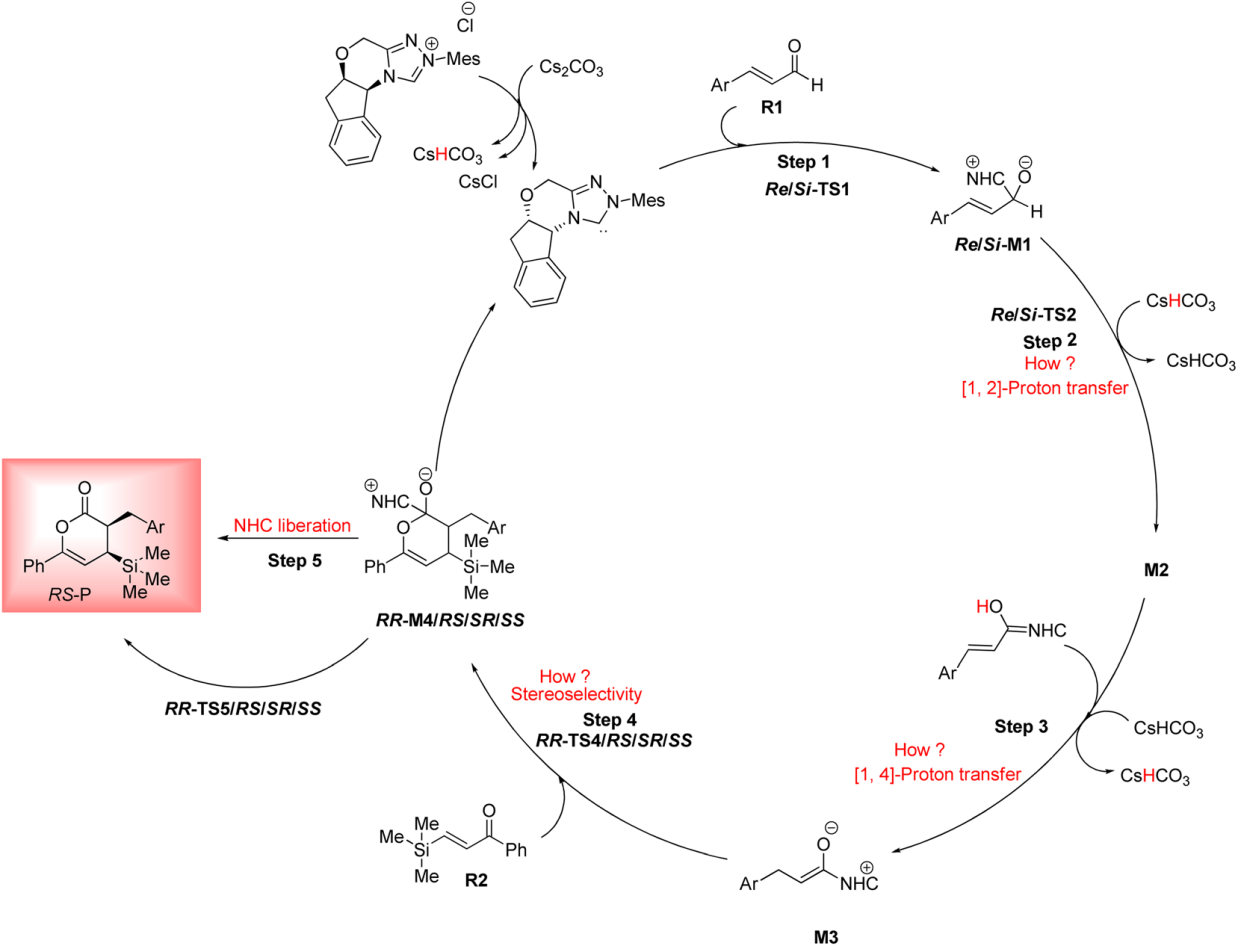
Possible mechanism for the NHC-catalyzed [4+2] annulation reaction of (*E*)-3-(*p*-tolyl)acrylaldehyde (R1) and phenyl-3-(trimethylsilyl)prop-2-en-1-one (R2).

As shown in Scheme 4, a possible mechanism has been proposed for this reaction by initiating the removal of a proton of the pre-catalyst by Cs_2_CO_3_ base to prepare the active NHC, a molecule of CsCl and a molecule of CsHCO_3_. The catalytic cycle involves five steps: (1) the formation of a zwitterionic intermediate by the addition of NHC to R1; (2) [1,2]-proton transfer processes to form the Breslow intermediate; (3) [1,4]-proton transfer processes to generate the enolate intermediate; (4) formal [4+2] annulation reaction or Diels–alder reaction; (5) regeneration of NHC to afford the expected product.

#### 3.1.1. First step: the formation of the zwitterionic intermediate *Re*/*Si*-M1 by the addition of NHC to R1

The first step commences with the nucleophilic attack of the NHC catalyst on the *Re*/*Si*-face carbonyl group of R1 to afford the intermediate *Re*/*Si*-M1, known as the zwitterionic intermediate, *via* the transition state *Re*/*Si*-TS1. The optimized geometries demonstrate that the distance between C1 and C2 atoms reduces from 1.92/1.94 Å in *Re*/*Si*-TS1 to 1.58/1.56 Å in *Re*/*Si*-M1, respectively (ESI†). This showed that the C1–C2 bond is completely coordinated between NHC and R1 in the first step. DFT calculations indicated that the energy barrier *Re*-TS1 (13.88 kcal mol^−1^) is 0.80 kcal mol^−1^ lower than that of *Si*-TS1 (14.68 kcal mol^−1^). More importantly, *Re*-M1 (9.69 kcal mol^−1^) is 3.24 kcal mol^−1^ lower than that of *Si*-M1 (12.93 kcal mol^−1^). These results show that the nucleophilic addition of NHC to the *Re*-face of R1 is both thermodynamically and kinetically favorable.

#### 3.1.2. Second step: formation of Breslow intermediate *Re*/*Si*-M2 by [1,2]-proton transfer

**Scheme 5:**
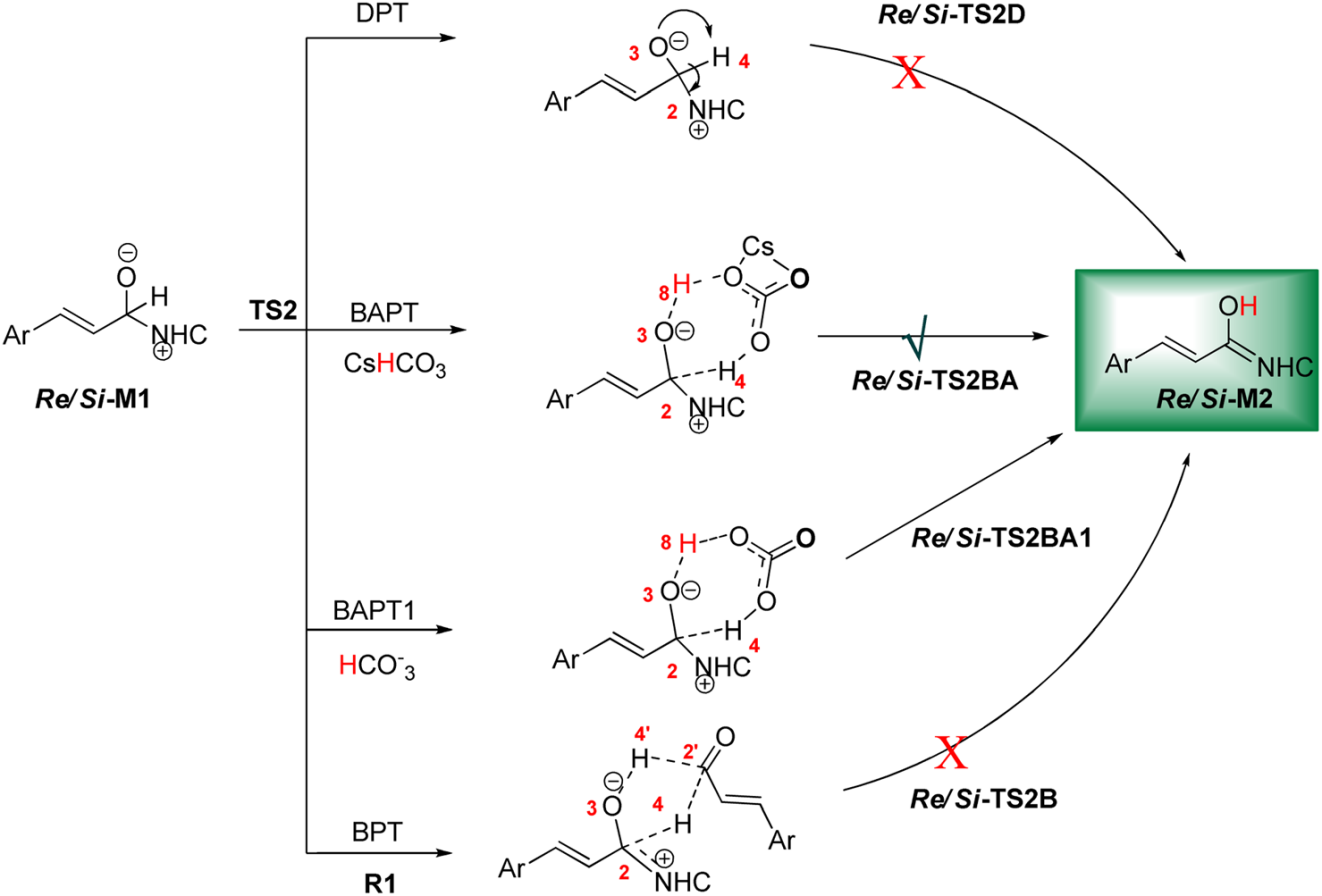
The possible pathways for the formation of the Breslow intermediate.

In this step, the Breslow intermediate *Re*/*Si*-M2 was obtained through the intramolecular proton transfer of H4 in the C2 to O3 atom of the intermediate *Re*/*Si*-M1 (Scheme 5). This process forms a three-membered ring with high strain due to the direct proton transfer (DPT) route. Inspired by the reported investigations in the literature,^78–82^ the other three possible routes are suggested, including the bimolecular proton transfer (BPT) happening using another molecule of enals R1. Cesium bicarbonate (CsHCO_3_) works as a medium for facilitating and assisting proton transfer that was obtained from the reaction between Pre-Cat with Cs_2_CO_3_ base and is shown as the BAPT route. Also, the bicarbonate anion (HCO_3_^−^) serves as a medium that assisted in proton transfer and depicted as the BAPT1 route (Scheme 5). The transition states TS2D, TS2B, TS2BA, and TS2BA1 are denoted as the DPT, BPT, BAPT and BAPT1 routes, respectively.

DPT route: The optimized geometry of *Re*/*Si*-TS2D reveals the formation of a three-membered ring between C2, O3, and H4 atoms. The bond length of O3–H4 is reduced from 2.08/2.08 Å in *Re*/*Si*-M1 to 1.36/1.35 Å in *Re*/*Si*-TS2D, whereas the distance between C2 and H4 is elongated from 1.13/1.12 Å in *Re*/*Si*-M1 to 1.17/1.18 Å in *Re*/*Si*-TS2D, respectively (ESI†). The free energy barrier was calculated at 37.93/33.49 (kcal mol^−1^) in *Re*/*Si*-TS2D, which is challenging to overcome at room temperature.

BPT route: Using the reported DFT studies on the NHC-catalyzed dimerization,^79,82^ this route has been proposed. This mechanism describes a bimolecular proton transfer that occurs using another molecule of enals R1. When the enal R1 approaches intermediate *Re*/*Si*-M1, the H4′ atom is transferred from the C2′ atom to the O3 atom from *Re*/*Si*-M1 and the H4 atom is shifted from the C2 atom to the C2′ atom of R1. This leads to the formation of a five-membered ring (C2, H4, O3, C2′, and H4′) through the *Re*/*Si*-TS2B transition state. The energy barrier (37.61/45.35 kcal mol^−1^ in *Re*/*Si*-TS2B) is higher than that of *Re*/*Si*-TSD, which is difficult to overcome at room temperature. The optimized geometry of *Re*/*Si*-TS2B indicates that these two proton transfers are asynchronous, and the transfer of the H4′ atom occurs faster than that of the H4 atom.

**Fig. 1:**
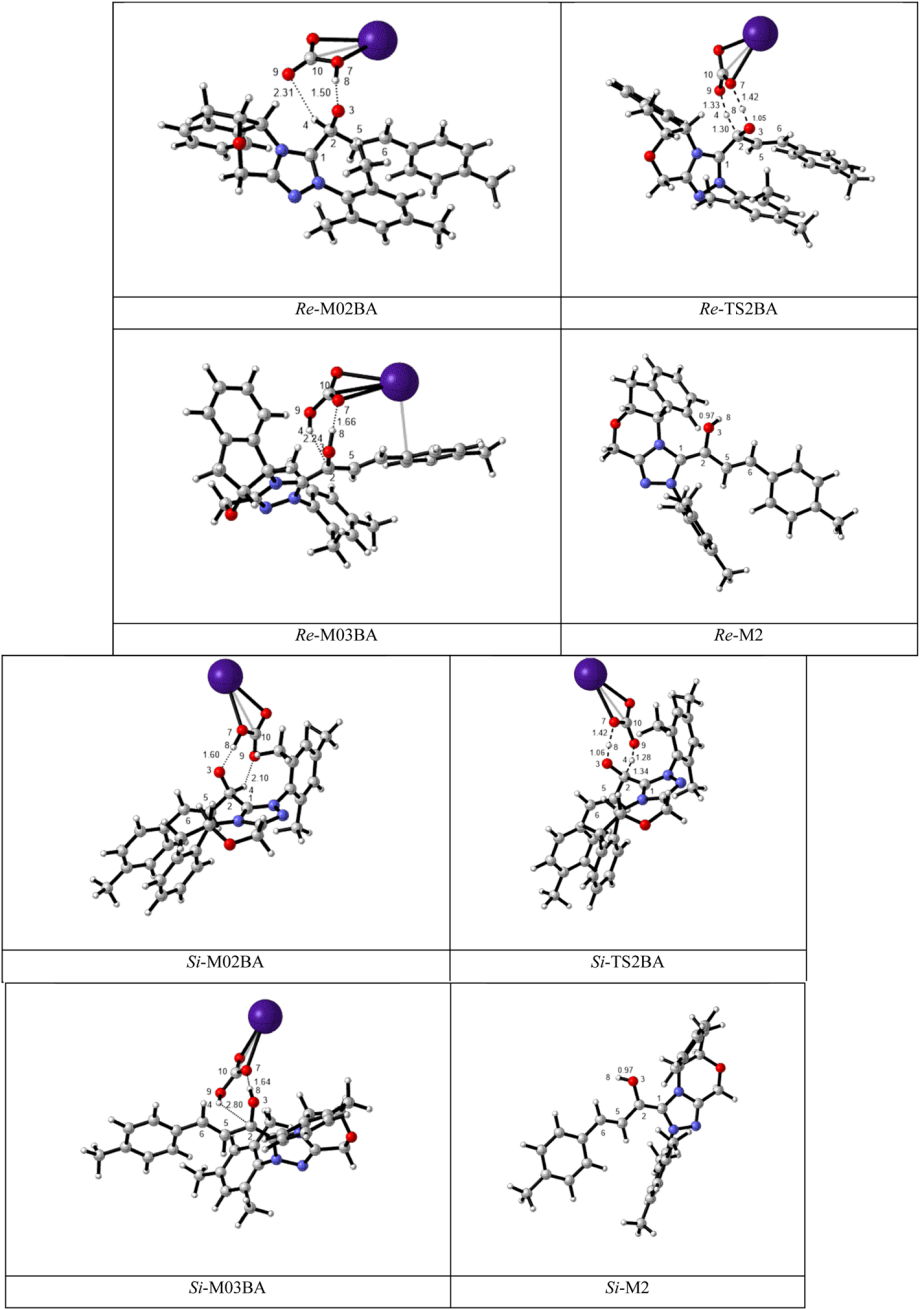
The geometry and optimization of the BAPT route for the formation of the Breslow intermediate.

BAPT route: As shown in Fig. 1, this route is scrutinized as an intermolecular proton transfer process in which cesium bicarbonate (CsHCO_3_) serves as the base and assists in performing proton transfer under the experimental conditions. The investigation of this mechanism was inspired by the reported studies in the literature.^82,83^ Cesium bicarbonate, for the aid of proton transfer, can reduce the energy barrier through the formation of the seven-membered ring (C2–H4–O9–C10–O7–H8–O3). This mechanism is more energetically favorable than that of the described mechanisms. As shown in Fig. 1, *Re*/*Si*-M1 in the presence of CsHCO_3_ forms *Re*/*Si*-M02BA. Next, *Re*/*Si*-M02BA performs the proton transfer through the transition state *Re*/*Si*-TS2BA and produces *Re*/*Si*-M03BA. In this *Re*/*Si*-TS2BA, the H4 is shifted from the C2 atom to the O9 of carbonyl, and following the hydrogen, H8 from the O7 atom is transferred to the O3 atom. The calculated free energy barrier was 9.23/14.49 kcal mol^−1^ in *Re*/*Si*-TS2BA, which has the lowest energy barrier of all the routes and is easily performed under experimental conditions. In addition, the energy barriers of *Re*/*Si*-M02BA and *Re*/*Si*-M03BA were obtained as 9.96/15.87 and −3.95/−4.55 kcal mol^−1^, respectively. These outcomes show that CsHCO_3_ can be easily linked with *Re*/*Si*-M1. In contrast, the energy barrier *Re*/*Si*-M03BA shows that CsHCO_3_ is quickly dissociated and the Breslow intermediate *Re*/*Si*-M2 is formed. The formation of a seven-membered ring leads to decreased strain and energy barriers compared to the other routes. Owing to the ring expansion, the reactivity centers increase in three-, five-, and seven-membered rings, respectively. As discussed above, the desired catalytic cycle was performed and continued using *Re*-M2 because the complexation of NHC and R1 (*Re*-face) in both thermodynamics and kinetics is more favorable than the coupling of NHC and R1 (*Si*-face). Therefore, the mechanism of the nucleophilic attack of NHC on the *Si*-face of R1 is ignored.

**Scheme 6:**
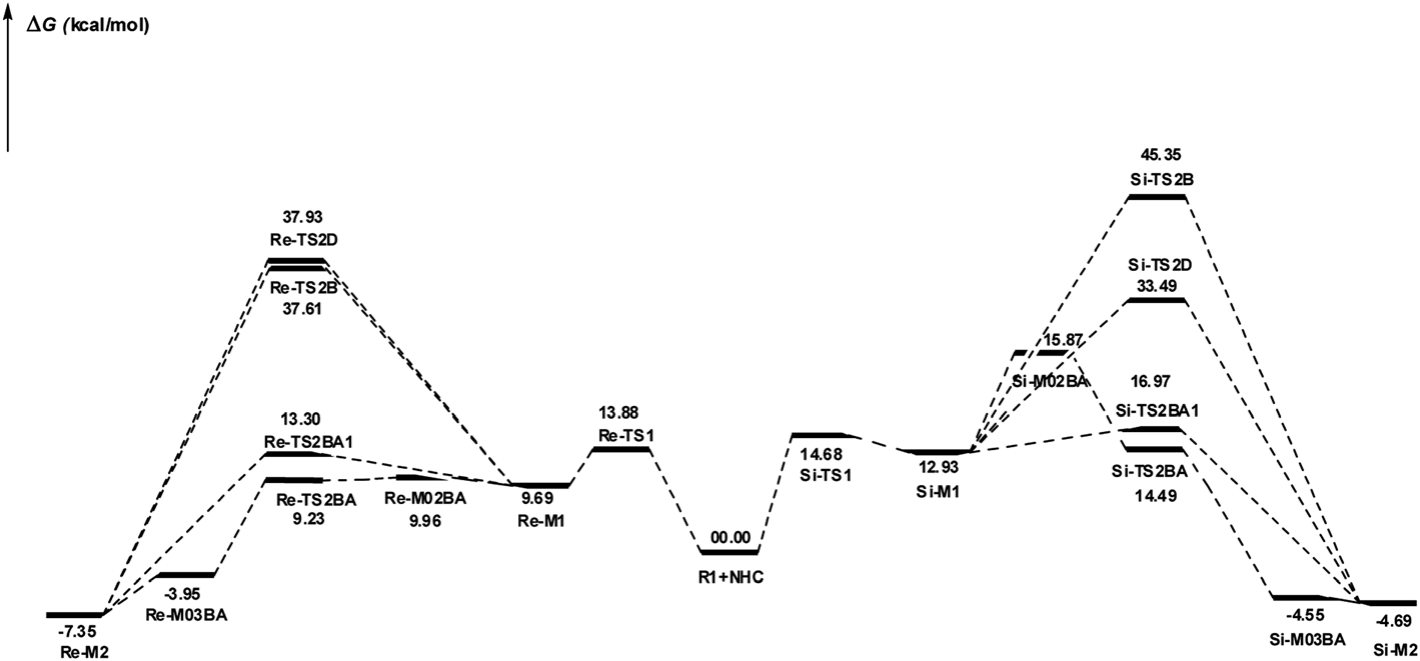
Gibbs free energy profiles of steps 1 and 2.

BAPT1 route: The bicarbonate anion (HCO_3_^−^) is also used for assisting proton transfer, which decreased the energy barrier by forming a seven-membered ring (C2–H4–O9–C10–O7–H8–O3). This route is more energetically favorable than that of the DPT and BPT routes. The calculated free energy barrier is 13.30/16.97 kcal mol^−1^ in *Re*/*Si*-TS2BA1, which has a high energy barrier in the BAPT mechanism and can be performed under experimental conditions. Notably, the strong interactions between the cesium and oxygen atoms lead to reduce energy barrier of the BAPT mechanism (BAPT *vs.* BAPT1) and were obtained simply under the experimental conditions. Scheme 6 illustrates the relative Gibbs free energy to construct the Breslow intermediate in both the first and second steps.

#### 3.1.3. Third step: formation enolate intermediate M3 by [1,4]-proton transfer

**Scheme 7:**
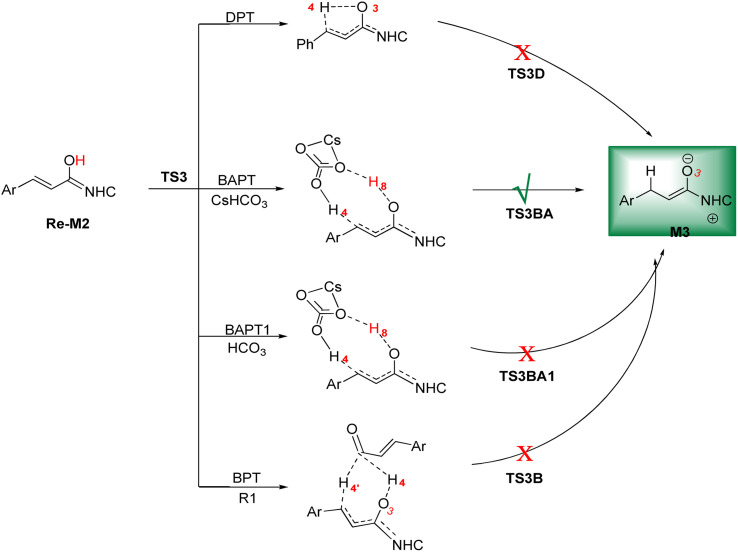
The possible routes for the formation of the enolate intermediate.

In this step, the Breslow intermediate *Re*-M2 will undergo β-protonation to produce the enolate intermediate M3. In fact, the [1,4]-proton transfer (intramolecular proton transfer) of H4 atom from the O3 to the C6 atom in the intermediate *Re*-M2 occurs (Scheme 6). Like step 2, direct proton transfer (DPT), bimolecular proton transfer (BPT), cesium bicarbonate-assisted proton transfer (BAPT) and the anion bicarbonate-assisted proton transfer (BAPT1) mechanisms were investigated (Scheme 7). The transition states TS3D, TS3B, TS3BA, and TS3BA1 are shown as the DPT, BPT, BAPT, and BAPT1 routes, respectively.

DPT route: This process forms a five-membered ring *via* the [1,4]-proton transfer. The optimized geometry of TS3D shows that a five-membered ring between C2, C5, C6, O3, and H4 atoms was formed. The bond length of O3–H4 was elongated from 0.97 Å in *Re*-M2 to 1.27 Å in TS3D, whereas the distance between C6 and H4 was reduced from 2.83 Å in *Re*-M2 to 1.43 Å in TS3D, respectively (ESI†). For this TS, the energy barrier was calculated to be 32.37 kcal mol^−1^.

BPT route: This mechanism explored a bimolecular proton transfer using another molecule of enals R1, which is done in two steps; firstly, the enal R1 approaches intermediate *Re*-M2, and the H4′ atom is transferred from the C2′ atom of R1 to the C6 atom of *Re*-M2. Following this, the H4 atom is shifted from the O3 atom to the C2′ atom of R1. For the transfer of the H4′ atom to the C6 atom in the first step, the energy barrier (61.12 kcal mol^−1^) is more than that of the DPT route, which leads to neglecting the transfer of H4 to C2′ calculation.

**Fig. 2:**
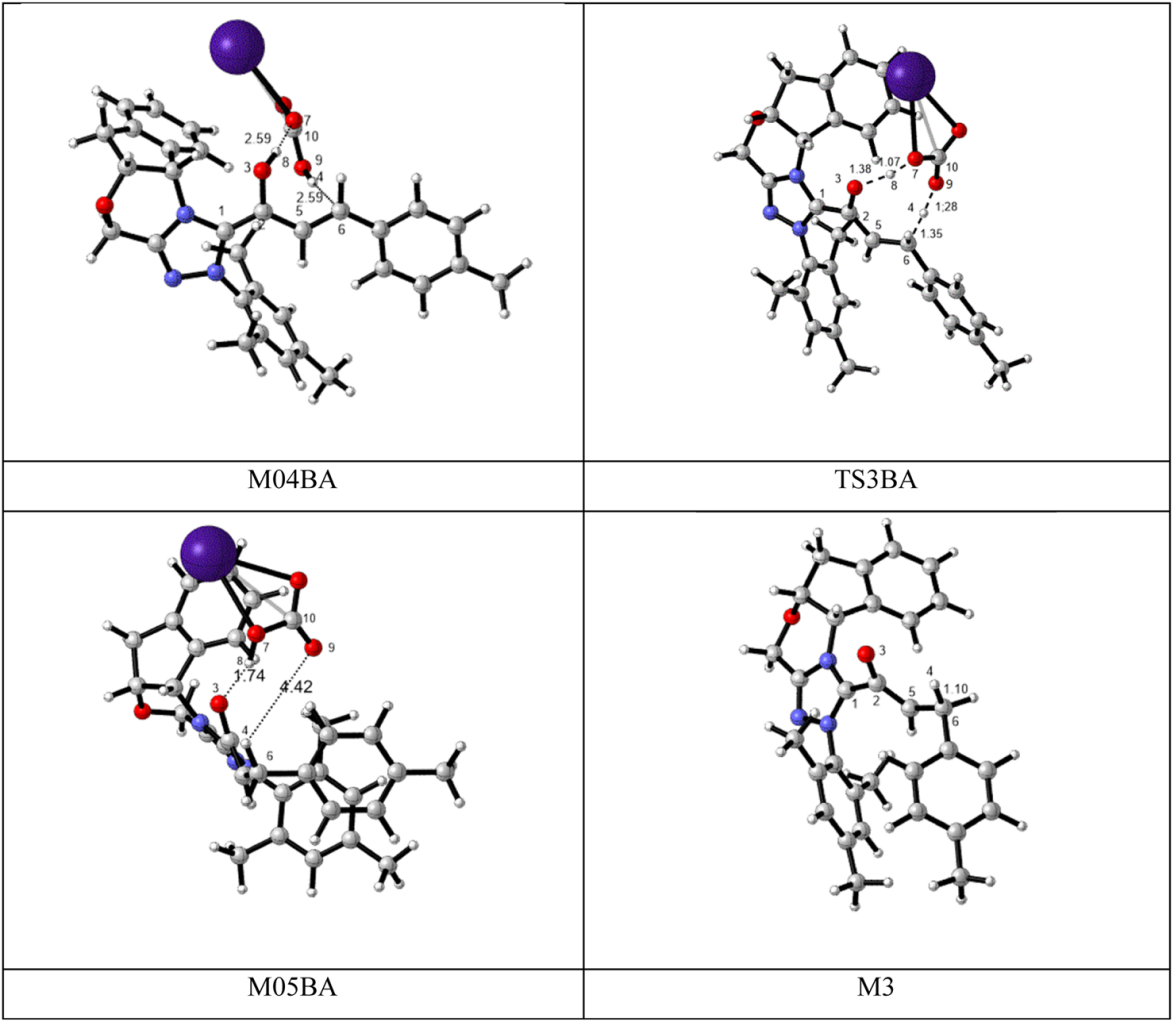
The optimized structures of the BAPT route for the formation of the enolate intermediate.

BAPT route: As depicted in Fig. 2, this route is described as an intermolecular proton transfer process in which the cesium bicarbonate base works as the medium for the proton-assisted transfer process. Cesium bicarbonate reduces the energy barrier through the formation of the nine-membered ring (C2, H8, O3, C5, C6, O7, H4, O9, and C10 atoms). This mechanism is more energetically favorable than that of other routes. As depicted in Fig. 2, CsHCO_3_ and *Re*-M2 formed intermediate M04BA. Then, M04BA undergoes the proton transfer through the transition state TS3BA and creates intermediate M05BA. In this T3BA, the H8 is shifted from the O3 atom to the O7 of carbonyl, following which the hydrogen H4 from the O9 atom in cesium bicarbonate is transferred to the C6 atom in *Re*-M2. The calculated free energy barrier is 19.48 kcal mol^−1^, the lowest barrier in this step and is easily performed under experimental conditions. In addition, the calculated results show that the energy barriers of M04BA and M05BA are −7.53 and −2.01 kcal mol^−1^, indicating that CsHCO_3_ is highly prone to react with *Re*-M2; in contrast, the energy barrier of M05BA shows that CsHCO_3_ is easily separated and formed enolate intermediate M3.

BAPT1 route: The bicarbonate anion applied for the aid of proton transfer diminished the energy barrier by making a nine-membered ring (C2–H4–O9–C10–O7–H8–O3). This pathway is more thermodynamically favorable than that of the DPT and BPT routes. The calculated free energy barrier is 22.37 kcal mol^−1^ in TS3BA1, which has a high energy barrier of the BAPT pathway and can be done under experimental conditions. Due to the presence of the Cs atom in the BAPT route, the dative interactions between the O and Cs atoms lead to reduced barrier energy (stability) *versus* the BAPT1 route.

#### 3.1.4. Fourth step: formal [4+2] annulation reaction

**Scheme 8:**
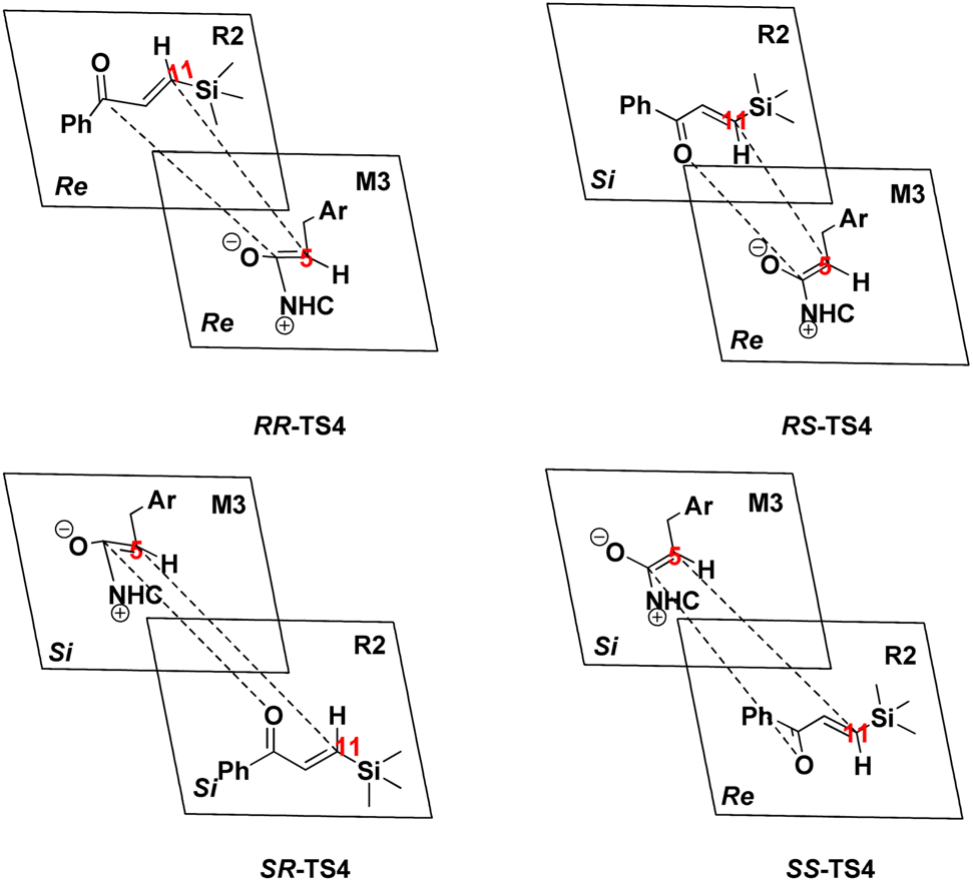
Stereochemistry for the [4+2] annulation reaction in the fourth step.

The fourth step is known as [4+2] annulation and also is the stereo-controlling step. This step determines that the *RS*-configuration is favorable to form the final product through the construction of two stereogenic centers (C5 and C11 atoms). Simultaneously, the C–O bond forms to produce a six-membered ring structure (M4). The six-membered ring *RR*, *RS*, *SR*, and *SS*-M4 were formed *via* transition state *RR*, *RS*, *SR*, and *SS*-TS4, respectively. The energy barriers were calculated for *RR*, *RS*, *SR*, and *SS*-TS4, 25.18, 12.47, 17.55, and 28.73 (kcal mol^−1^), respectively, implying that all four configurations can be generated at the experimental conditions. The calculated energy barrier *RS*-TS4 is 12.47 kcal mol^−1^, the lowest among other configurations. The energy barriers are 2.88, 2.98, −2.21, and 1.31 (kcal mol^−1^) for *RR*, *RS*, *SR*, and *SS*-M4, respectively. The four possible reaction models for this step are shown in Scheme 8. The addition from the *Re* face of M3 on the *Re* or *Si* face of R2 results in the formation of intermediates *RR*-M4 and *RS*-M4 through *RR*-TS4 and *RS*-TS4, respectively. The addition of the *Si* face from M3 to *Re* or the *Si* face from R2 results in intermediates *SR*-M4 and *SS*-M4 through *SR*-TS4 and *SS*-TS4, respectively. The geometrical parameters show that from *RR*, *RS*, SR, and *SS*-TS4 to *RR*, *RS*, *SR*, and *SS*-M4, the bond distance C5–C11 shortens from 2.30, 2.24, 2.21, and 2.20 Å to 1.50, 1.54, 1.54, and 1.55 Å, and also the bond distance C2–O14 is decreased from 2.73, 2.92, 2.98, and 2.94 Å to 1.53, 1.51, 1.50, and 1.50 Å, respectively (ESI†).

Generally, the addition of the *Re* face from M3 to the *Re* or *Si* face of R2 is more stable and prominent both kinetically and thermodynamically compared to the addition of the *Si* face from M3 to the *Re* or *Si* face of R2.

#### 3.1.5 Fifth step: catalyst NHC liberation

**Scheme 9:**
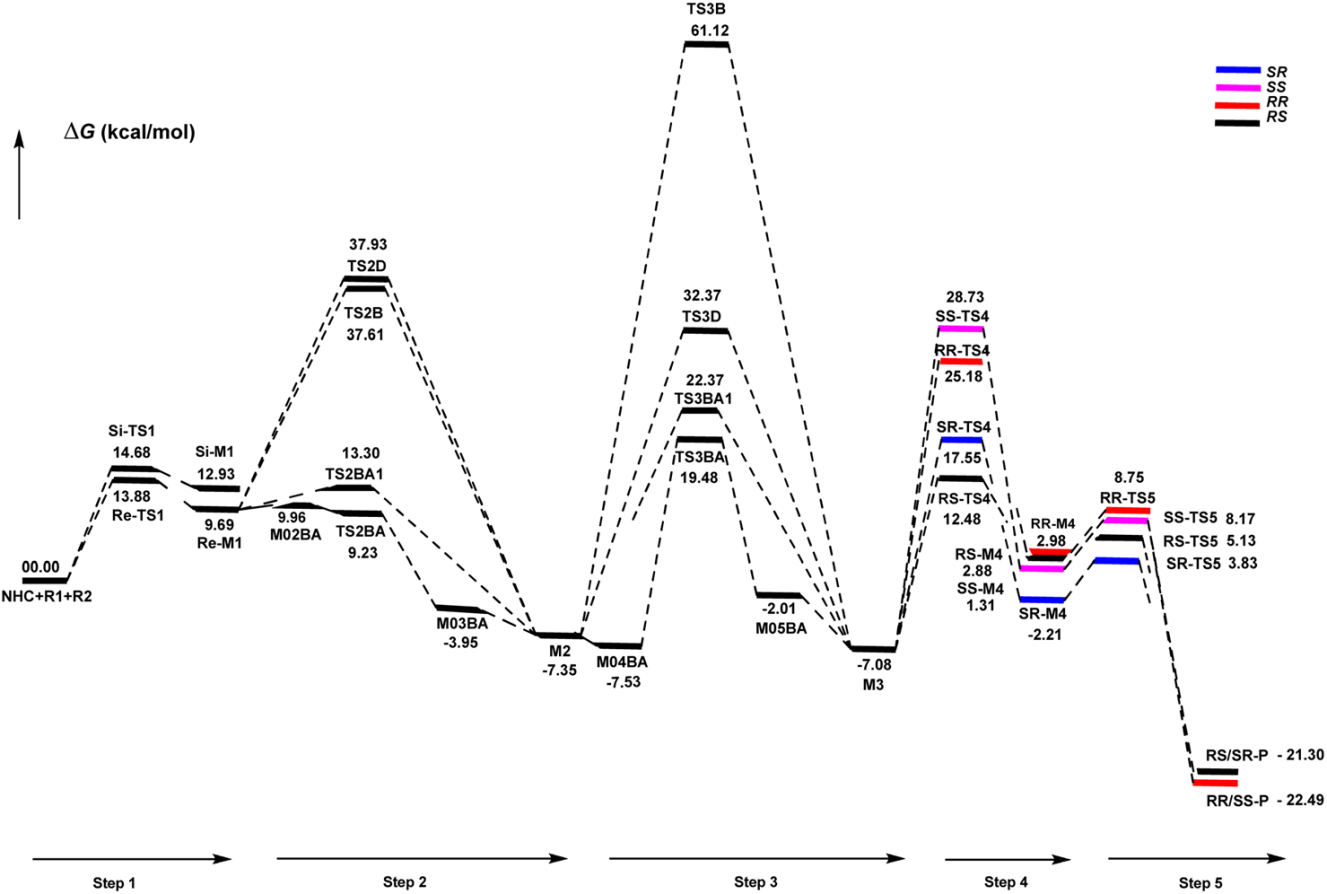
Gibbs free energy profiles of steps 1–5.

Finally, the NHC catalyst is released from intermediate M4 to synthesize the expected cycloaddition product *RS*-P *via RS*-TS5. The energy barriers for *RR*, *RS*, *SR*, and *SS*-TS5 were calculated at 8.75, 5.13, 3.83, and 8.18 kcal mol^−1^, respectively. Owing to the small energy barrier, the regeneration of the NHC catalyst is very facile. From *RR*-M4, *RS*. *SR*, and *SS* to *RR*, *RS*. *SR*, and *SS*-TS5, the bond distance of C1–C2 is elongated from 1.58, 1.58, 1.57, and 1.58 Å, to 2.03, 1.99, 2.04, and 2.01 Å, respectively (ESI†). Lastly, the relative Gibbs free energy profiles of steps 1–5 are shown in Scheme 9.

### 3.2. The second reaction route

**Scheme 10:**
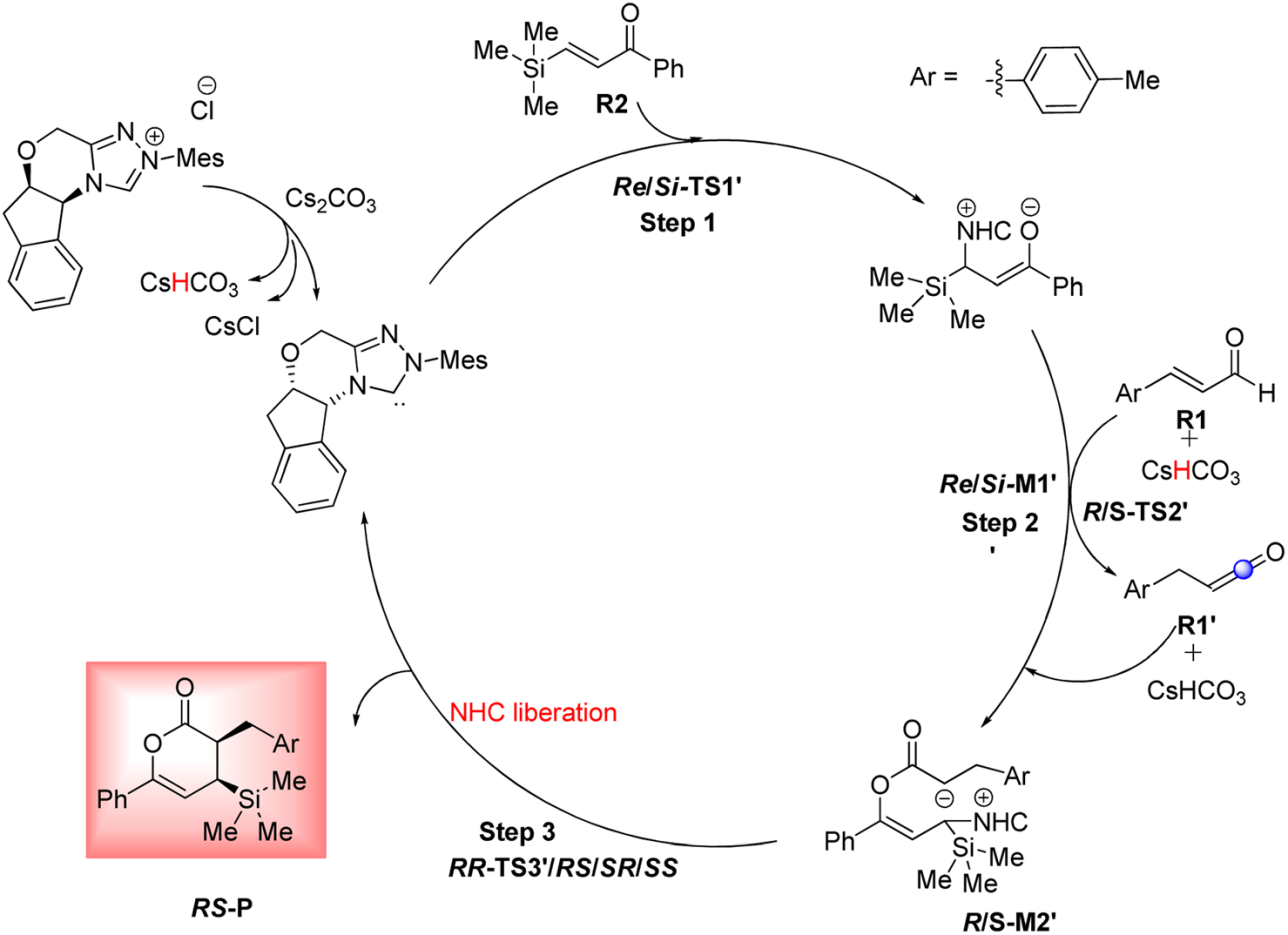
The second reaction mechanism for the NHC-catalyzed annulation reaction of (*E*)-3-(*p*-tolyl)acrylaldehyde (R1) and phenyl-3-(trimethylsilyl)prop-2-en-1-one (R2).

Based on Wei's research in 2014,^41^ this route is suggested to further investigate the reaction of R2 and NHC catalyst. As shown in Scheme 10, the catalytic cycle recognizes three steps: (1) the formation of zwitterionic intermediate *Re*/*Si*-M1′ by the Michael addition of NHC to *Re*/*Si*-R2; (2) the construction of the C–O bond through the addition of *Re*-M1′ to R1 (keten); (3) the formation of the chiral C–C bond *via* intermolecular annulation reaction and regeneration of NHC to afford the expected product. For performing this pathway, it essentially converted the (*E*)-3-(*p*-tolyl)acrylaldehyde (R1) to the keten molecule (R1′) *via* [1,3]-proton transfer, where the H4 from C2 atom is transferred to the C6 atom. The conversion of R1 to R1′ was studied through three mechanisms including the direct proton transfer (DPT) through the formation of the four-membered ring, the bimolecular proton transfer (BPT) through the help of another R1 by forming the six-membered ring, and the aid of CsHCO_3_ base (BAPT) by making the eight-membered ring. The calculated results indicated that the energy barrier for these TSs is 65.34, 75.17, and 51.56 kcal mol^−1^ for the DPT, BPT, and BAPT pathways, respectively. Owing to the instability of the formed four, six, and eight-membered ring structures in these TSs, the preparation of the keten molecule (R1′) structure from the enal (R1) is impossible under ambient temperatures. Additionally, we investigated the first step of this route that started with the Michael addition of the NHC on the *Re*/*Si*-face of C11 from R2 to afford the intermediate *Re*/*Si*-M1′, identified as the zwitterionic intermediate *via* the transition state *Re*/*Si*-TS1′. The optimized geometries demonstrate that the distance between C1 and C11 atoms is 2.06/2.01 Å in *Re*/*Si*-TS1′, respectively. The DFT outcomes indicated that the energy barrier *Re*/*Si*-TS1′ (17.22/18.24 kcal mol^−1^) is 3.34/3.56 kcal mol^−1^ higher than that of *Re*/*Si*-TS1 (13.88/14.68 kcal mol^−1^). These outcomes confirm that the nucleophilic addition of NHC to the *Re*-face of R1 is both thermodynamically and kinetically favorable than the Michael addition of the NHC catalyst on the *Re*/*Si*-face of R2. Therefore, we did not continue the other steps and ignored the second reaction route.

### 3.3. Origin of the stereoselectivity

As discussed above, approaching the intermediate M3 to R2, the two chiral centers (C5 and C11 atoms) formed in the stereoselectivity step. Therefore, the transition states *RR*-TS4, *RS*-TS4, *SR*-TS4, and *SS*-TS4 are crucial to deeply understanding this mechanism. The energy difference between *RS*-TS4 and *SR*-TS4 is 5.08 kcal mol^−1^, corresponding to an enantiomeric excess of >99% in favor of the *RS* diastereomer, and the free energy difference between *RS*-TS4 and *RR*-TS4 (12.71 kcal mol^−1^) can be associated with the diastereomeric excess. These results are in good agreement with the experimentally observed ee of 99%. Herein, the origin of the stereoselectivity is described for the NHC-catalyzed [4+2] annulation reaction. The distortion/interaction analysis was performed to comprehensively understand the stereoselectivity of the transition state TS4. Based on Houk's definition,^84^ each transition state is dissociated into two parts (*i.e.*, M3_part and R2_part). The distortion energy is defined as the energy level difference between the decomposed part and the corresponding optimized structure in the absence of the other part (*i.e.*, Δ*E*^≠^_dist_M3_ and Δ*E*^≠^_dist_R2_), and the total distortion energy (Δ*E*^≠^_dist_total_) is defined as Δ*E*^≠^_dist_M3_ + Δ*E*^≠^_dist_R2_. The interaction energy is obtained through the relationship Δ*E*^≠^_int_ = Δ*E*^≠^ − Δ*E*^≠^_dist_. The computed distortion and interaction energies are obtained for the reactants in the transition state geometries (Table 1). The distortion/interaction energies were compared for these four transition states. These results show that the distortion energy of *RS*-TS4 (17.92 kcal mol^−1^, Table 1) is substantially lower than that of *RR*-TS4 and *SR*-TS4 (29.55 and 21.79 kcal mol^−1^, Table 1). In addition, the most stable interaction energy (−1.68 kcal mol^−1^, Table 1) of *RS*-TS4 is presented. The most stable interaction energy and the distortion energy are observed for *RS*-TS4. These data determine that the *RS*-configuration product is preferentially formed.

**Table 1:** The distortion and interaction energies for the reactants of geometries TS4

SR	ΔΔ*G*	Δ*E*^≠^	Δ*E*^≠^_dist_ (M3)	Δ*E*^≠^_dist_ (R2)	Δ*E*^≠^_dist_ (total)	Δ*E*_int_
*RR*-TS4	12.71	30.01	15.33	14.22	29.55	0.46
*RS*-TS4	00.00	16.24	8.58	9.34	17.92	−1.68
*SR*-TS4	5.08	21.97	12.21	9.58	21.79	0.18
*SS*-TS4	16.26	33.41	16.77	10.32	27.08	6.33

The atom in molecules (AIM) analysis was also applied to further gain insight into the stereo-controlling step. and distinguish the nature of interactions of the chiral C5–C11 bond in TS4 using the properties of electron density by indicating the bond critical points (BCPs). These desirable topological parameters, such as electron density, *ρ*(*r*), Laplacian of electron density, ∇^2^*ρ*(*r*) and electronic energy density, *H*(*r*) interaction for the transition states *RR*, *RS*, *SR*, and *SS*-TS4, are shown in Table 2. According to this table, the C5–C11 bond should be considered as a partially electrostatic and covalent interaction (1 < |*V*(*r*)|/*G*(*r*) < 2).

**Table 2:** The AIM analysis for the transition states *RR*-TS4, *RS*, *SR*, and *S*

Compound	*ρ*(*r*)	∇^2^*ρ*(*r*)	*H*(*r*)	*V*(*r*)	*G*(*r*)	|*V*(*r*)|/*G*(*r*)
*RR*-TS4	0.049	0.048	−0.008	−0.028	0.020	1.40
*RS*-TS4	0.059	0.049	−0.011	−0.034	0.023	1.48
*SR*-TS4	0.057	0.047	−0.013	−0.037	0.025	1.48
*SS*-TS4	0.058	0.047	−0.013	−0.038	0.025	1.52

**Fig. 3:**
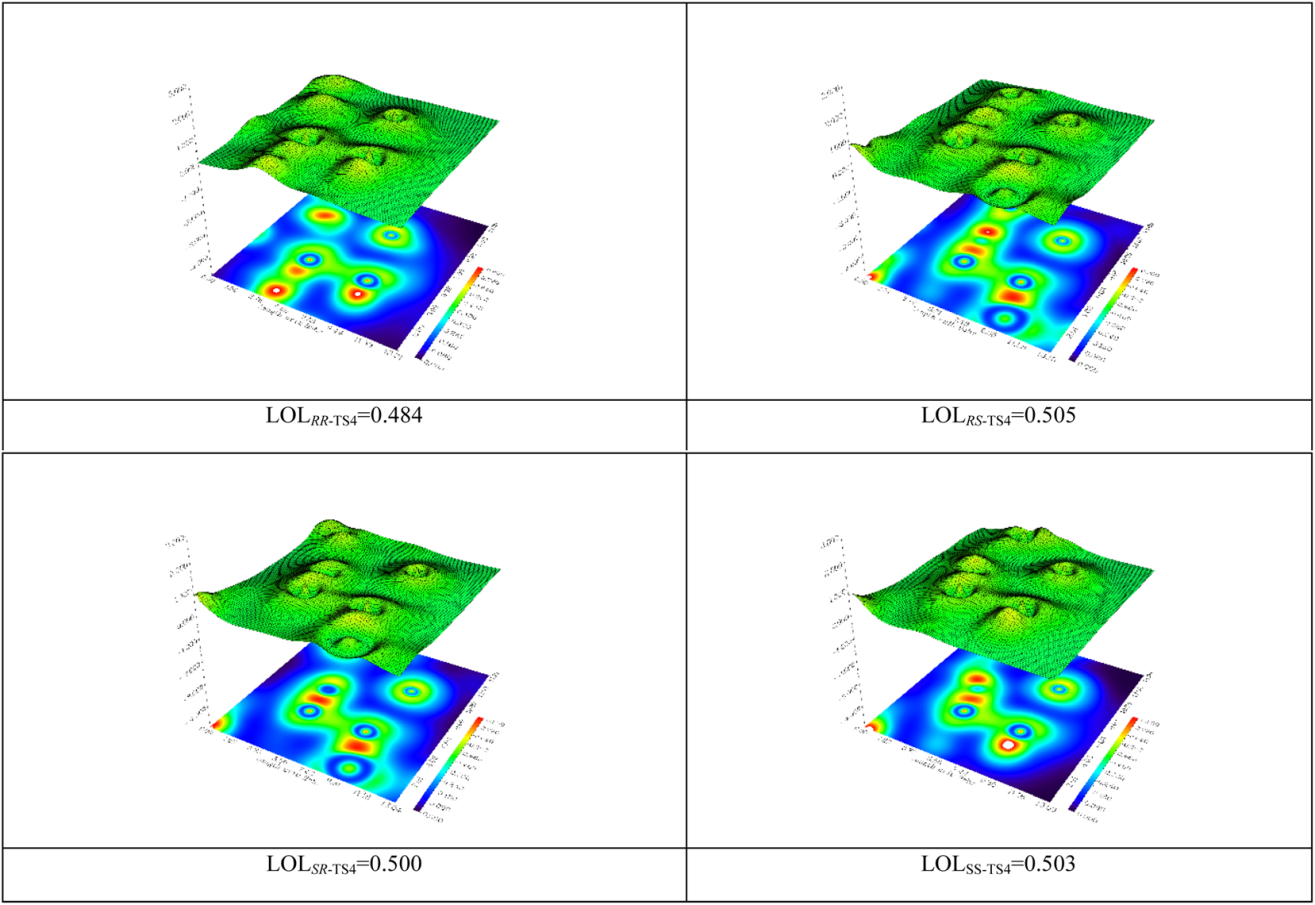
The LOL analysis for the transition states *RR*-TS4, *RS*, *SR*, and *SS*.

The localized orbital locator (LOL) map was calculated (Fig. 3).^58,59^ The obtained results illustrate that there is usually a strong bond along with a high electron density at the BCP. This parameter shows that the C5–C11 bond (*RS*-TS4) is stronger than *RR*, *SR*, and *SS*-TS4.

**Fig. 4:**
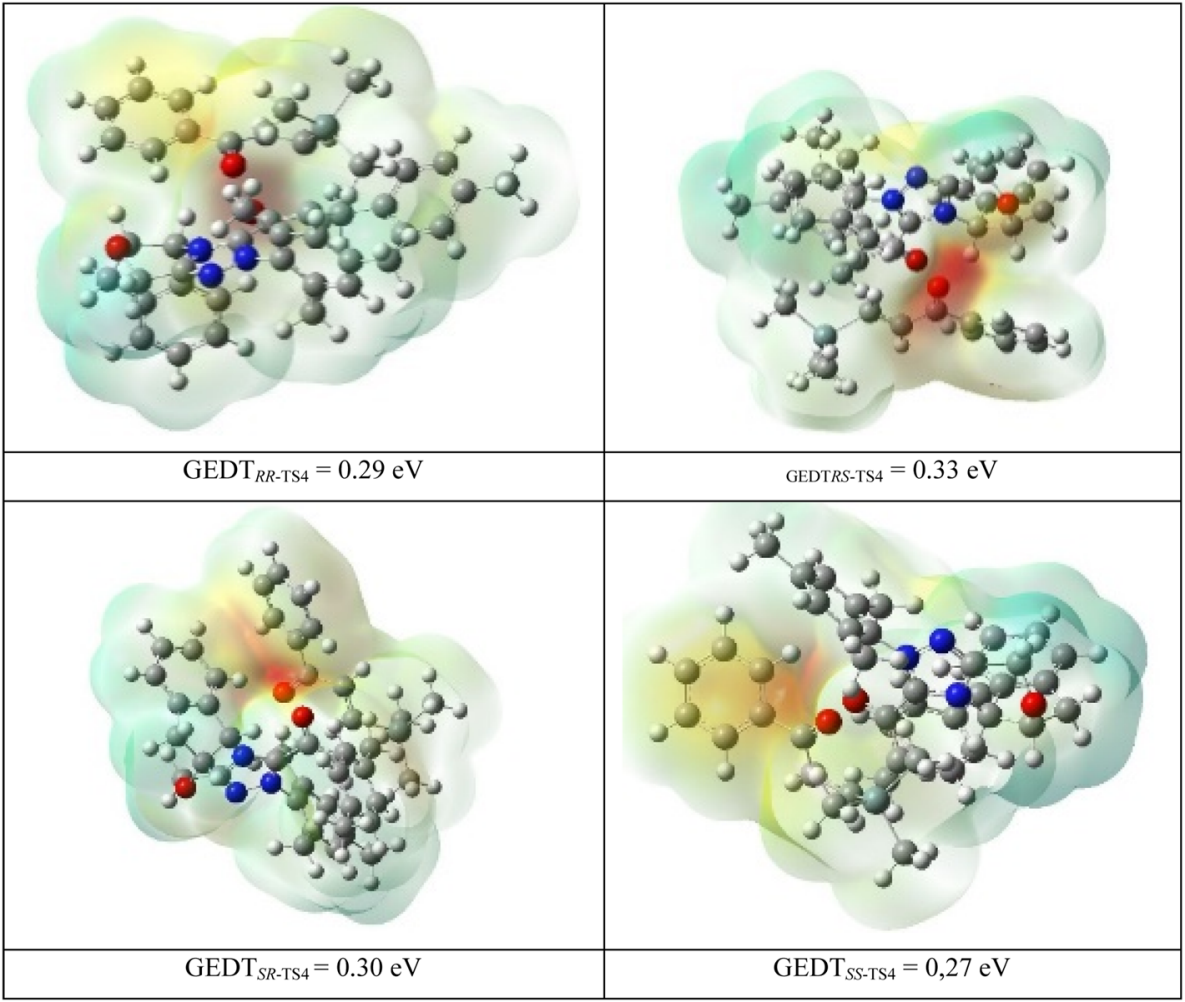
Molecular electrostatic potential (MEP) maps of TSs in the [4+2] annulation reaction step.

Also, global electron density transfer (GEDT)^85–87^ is used to disclose the kind of reaction and charge transfer in the transition states TS4. GEDT results are indicated along with the molecular electrostatic potential maps for *RR*, *RS*, *SR*, and *SS*-TS4 in Fig. 4. For the four transition states, the obtained GEDT indicates that R2 is the negative part and has a low electron density (LUMO), whereas M3 is the positive part with a high electron density (HOMO). The charge transfer of M3 to R2 confirms that the inverse Diels–Alder reaction occurred. It should be mentioned that the GEDT value for the four transition states is more than 0.15 eV. This GEDT value shows that the [4+2] reaction has a polar nature and absorption occurs in all of the transition states TS4.

### 3.4. Explication of the effect of substitution

As shown in Table 3, Parr Functions (*P*_k_^+^ and *P*_k_^−^) were studied to obtain the effect of the substitution for the R1 and M3 derivatives in the first and fourth steps. We chose chlorine (Cl) and hydrogen (H) atoms in contrast to the methyl group (Me) as the model to investigate the transition states of *Re*/*Si*-TS1 and *RR*, *RS*, *SR*, *SS*-TS4. Table 3 shows that the Cl atom in R1 reduces the barrier energy compared to the hydrogen and methyl derivatives of both the *Re* and *Si*-TS1. The local electrophilicity index *P*_k_^+^ increases the C2 atom of the Cl-R1 through the resonance effect with the phenyl ring. This phenomenon caused the treatment of Cl-R1 and NHC to proceed easily compared to the substituted H and Me-R1. It is observed that the barrier energy for the substituted *Re*-TS1 is lower than that of the substituted *Si*-TS1. Also, this table indicates that the substituted Cl atom in M3 decreases the barrier energy compared to the substituted H and Me derivatives in the *RR*, *RS*, *SR*, and *SS*-TS4. The local nucleophilicity index *P*_k_^−^ value of the C5 atom in Cl-M3 is 0.67 eV, while this value of *P*_k_^−^ for the C5 atom in H and Me-M3 is 0.66 and 0.65 eV, respectively. These results illustrate that the nucleophilicity of the C5 atom in the substituted Cl atom in M3 is increased compared to the substituted H and Me in M3 and leads a reduction in the barrier energy Cl-TS4s. The *RS* configuration has the lowest barrier energy of all the substituted TS4s.

**Table 3:** Parr functions (*P*_k_^+^ and *P*_k_^−^) to study the effect of substitution for the R1 and M3 derivatives in the first and fourth steps

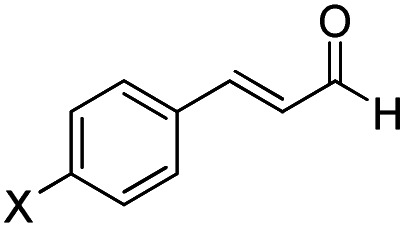			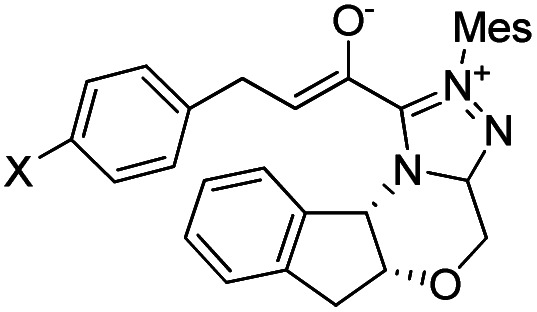			
R1			M3			
R1 (X)	*P*_k_^+^ (C2)	*P*_k_^−^ (C2)	*Re*-TS1	*Si*-TS1		
Cl	0.20	−0.10	11.88	12.83		
H	0.16	−0.11	12.91	13.88		
Me	0.16	−0.10	13.88	14.68		
M3 (X)	*P*_k_^+^ (C5)	*P*_k_^−^ (C5)	*RR*-TS4	*RS*-TS4	*SR*-TS4	*SS*-TS4
Cl	0.09	0.67	23.61	10.71	15.38	26.38
H	0.10	0.66	24.43	11.80	16.49	27.53
Me	0.10	0.65	25.18	12.47	17.55	28.73

Therefore, in our study, the observed substituent effects were primarily attributed to the electronic factors. The Parr functions employed provide a quantitative measure of local electron density, elucidating the impact of different substituents on the reaction centers. Though steric effects cannot be entirely discounted, especially for significantly bulky substituents, their influence in our system is considered secondary.

### 3.5. Explication of the nature of the NHC catalyst

To further understand the details of the NHC-catalyzed formation of chiral organosilanes through the [4+2] annulation of R1 and R2, it is essential that the role of the catalyst be revealed in the suggested catalytic cycle. The conceptual DFT (CDFT), FMO, LRI, and NBO analyses were applied to describe the role of NHC in the catalytic mechanism. Global reactivity indices derived from CDFT for the NHC, R1, *Re*-M1, M3, and R2 are listed in Table 4. Domingo's describes that the molecular electrophilicity feature is determined as the global electrophilicity index (*ω*), whereas the molecular nucleophilicity feature is quantified as the global nucleophilicity index (*N*). As depicted in Table 4, the catalyst NHC contains an electrophilicity value of 0.73 eV and a nucleophilicity value of 1.48 eV. On the other hand, the electrophilicity and nucleophilicity values of R1 are 1.508 and 1.56 eV, respectively. Thus, the NHC carbene coordinates successfully with R1. Moreover, it is observed that the interaction of NHC with R1 remarkably decreased the electrophilicity value to 0.79 eV; in contrast, the global nucleophilicity value increased to 2.73 eV in the *Re*-M1. In addition, the electrophilicity and nucleophilicity values are 0.76 and 3.40 eV for intermediate M3, respectively. Accordingly, the enolate intermediate M3 can now interact successfully with the electrophilic center of R2, which has a global electrophilicity value of 1.56 eV. These outcomes corroborated that the inversion of polarity occurs in R1.

**Table 4:** CDFT-based electronic chemical potential (*μ*, in a.u.), chemical hardness (*η*, in a.u.), global electrophilicity (*ω*, in eV), and global nucleophilicity (*N*, in eV) of NHC, R1, *Re*-M1, M3, and R2

SR	*μ*	*η*	*ω*	*N*
NHC	−0.12	0.29	0.73	1.48
R1	−0.16	0.23	1.51	1.56
*Re*-M1	−0.11	0.21	0.79	2.73
M3	−0.10	0.19	0.76	3.40
R2	−0.17	0.25	1.56	0.72

**Fig. 5:**
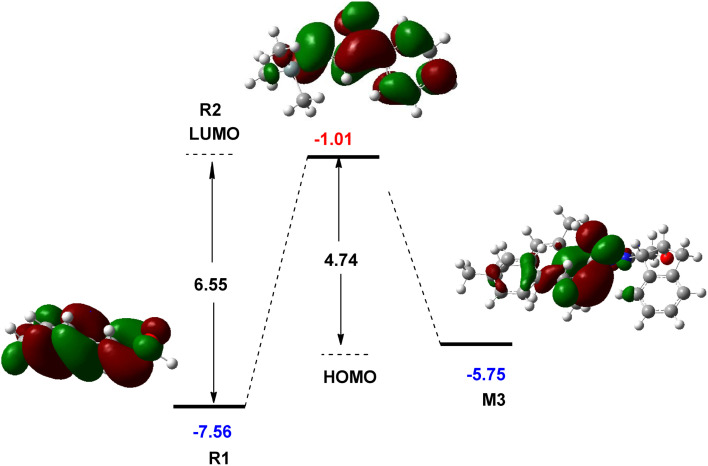
Calculated frontier molecular orbitals of R1, R2, and M3 at the M06-2X/6-31G(d,p) level (unit in eV).

The role of NHC catalyst in the catalytic cycle was evaluated by studying the FMO analysis. This theory is defined as the energies of the highest occupied and the lowest unoccupied molecular orbitals, known as the HOMO and LUMO energies, respectively. The FMO diagram for the interaction of R1 with R2 in the presence and absence of the NHC catalyst is shown in Fig. 5. This diagram illustrates that the reaction between R1 and R2 is not possible in the absence of an NHC catalyst at the experimental conditions owing to a higher energy gap (6.55 eV) between the HOMO and LUMO orbitals of R1 and R2, respectively. The incorporation of the NHC catalyst and R1 forms an intermediate M3 in which the HOMO energy level is 5.75 eV, reducing the LUMO–HOMO energy gap to 4.74 eV. This energy gap leads to the reaction with R2 under the experimental conditions. Owing to the existence of the NHC catalyst, the polarity of R1 changes by forming M3 and allows the annulation reaction to take place with R2. Thus, the description of FMO theory is well associated with the CDFT assessment, indicating the role of the NHC catalyst in the catalytic cycle of this reaction.

As shown in Table 5, the local reactivity index (LRI) was also assessed to obtain further insights into the nature of NHC at the M06-2X/6-31G(d,p) level of theory. This table shows more details about the Umpolung properties of the NHC catalyst. It is seen that the *ω*_k_ value of the C2 atom in R1 is 0.24 eV, while the *N*_k_ value of the C1 atom in NHC is 1.55 eV. The *ω*_k_ and *N*_k_ values of the O3 atom in R1 are 0.31 eV and 0.21 eV, changing to 0.004 eV and 2.33 eV in *Re*-M1, respectively. This shows that the polarity of the O3 atom in the generated *Re*-M1 is changed. The interaction of the C1 and C2 atoms in NHC and R1 changes the nature of the C5 atom in the generated M3. The *ω*_k_ and *N*_k_ values at the C5 atom of M3 were calculated to be 0.07 and 2.24 eV, respectively. The calculated LRI values show that reactant R2 has two electrophilic centers at C11 and C13 with *ω*_k_ values of 0.43 and 0.35 eV, respectively. Thus, the NHC organocatalyst promoted the annulation reaction between R1 and R2 under the experimental conditions.

**Table 5:** Parr functions (*P*_k_^+^ and *P*_k_^−^), local electrophilicity (*ω*_k_, in eV), and local nucleophilicity (*N*_k_, in eV) of NHC, R1, *Re*-M1, M3, and R2

SR	Atom	*P*_k_^+^	*P*_k_^−^	*ω*_k_	*N*_k_
NHC	C1	0.11	1.05	0.08	1.55
R1	C2	0.155	−0.10	0.24	−0.14
	O3	0.20	0.14	0.31	0.21
	C5	0.11	0.44	0.17	0.66
*Re*-M1	C2	−0.01	−0.08	−0.01	−0.21
	O3	−0.004	0.85	−0.004	2.33
	C5	0.02	0.08	0.02	0.23
M3	O3	0.02	0.45	0.01	1.52
	C5	0.10	0.66	0.07	2.24
R2	C13 (CO)	0.22	−0.08	0.35	−0.06
	C11 (C_β_)	0.27	−0.02	0.43	−0.01

As shown in Table 6, the NBO charge analysis illustrates that the charge on the C1 atom is enhanced from 0.14 e in NHC to 0.51 e in *Re*-M1, while the charge value on the C2 atom is reduced from 0.38 e in R1 to 0.10 e in *Re*-M1. Furthermore, the negative charge on the N atom in NHC reduces (−0.46 e in NHC *vs.* −0.39 e in *Re*-M1), whereas the negative charge on the oxygen atom increases (−0.57 e in R1 *vs.* −0.92 e in *Re*-M1). Also, the NBO charge of the H4 atom is enhanced slightly from 0.16 e in R1 to 0.17 e in *Re*-M1. The cooperation of NHC and R1 increases the acidity of the H4 atom, which makes the breakage of the C2–H4 bond easier. NBO results show that the electron is shifted from the NHC catalyst to R1 in the process of NHC + R1 → *Re*-M1. The increased NBO charge of the O3 and H4 atoms in the conversion of (R1 → *Re*-M1) would remarkably facilitate the proton transfer of H4 to the O3 atom. Therefore, NHC activates the C2–H4 bond of R1 by enhancing the acidity of the H4 atom and improving the feasibility of bond fracture. In addition, many investigations have indicated that NHC mainly serves as a Lewis base in the annulation reaction.

**Table 6:** The NBO charges of selected atoms NHC, R1, *Re*-M1, M3, and R2 (units of *e*)

SR									
NHC		R1			*Re*-M1				
N	C1	C2	O3	H4	N	C1	C2	O3	H4
−0.46	0.14	0.38	−0.57	0.16	−0.39	0.51	0.10	−0.92	0.17

To predict and assay the catalytic efficiency of the NHC catalyst compared to the achiral NHC (A), we carried out additional calculations on the stereoselectivity-determining step (Table 7). The energy barrier for *RS*-TS4_NHC (A) is 6.94 kcal mol^−1^, which is higher than that of the *RS*-TS4_NHC (B). These results are consistent with the experimental observations, *i.e.*, the NHC provides higher reaction yields than NHC (A).

**Table 7:** Comparison of the catalytic efficiency of the NHC catalyst with achiral NHC (A) in the stereo-controlling step

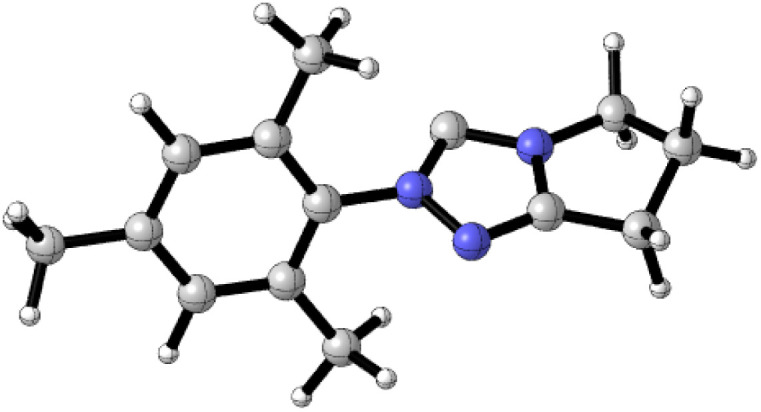		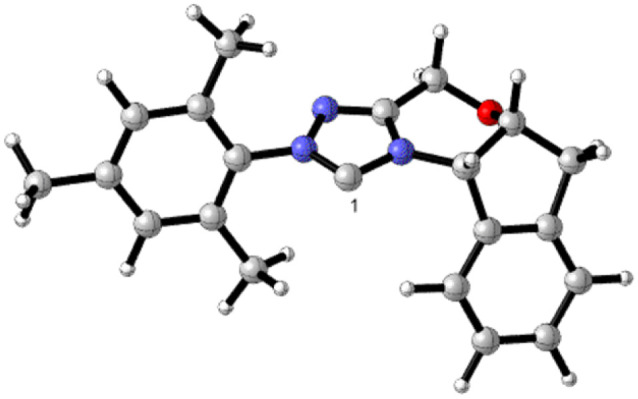	
Achiral NHC (A)		Chiral NHC (B)	
SR	Δ*G*^‡^	SR	Δ*G*^‡^
*RR*-TS4	17.31	*RR*-TS4	18.10
*RS*-TS4	6.94	*RS*-TS4	5.40
*SR*-TS4	6.42	*SR*-TS4	10.47
*SS*-TS4	20.83	*SS*-TS4	21.65

## 4. Conclusion

In this article, the detailed mechanisms and origin of stereoselectivity for the asymmetric [4+2] cycloaddition between an (*E*)-3-(*p*-tolyl)acrylaldehyde (R1) and phenyl-3-(trimethylsilyl)prop-2-en-1-one (R2) in the presence of NHC catalyst have been theoretically investigated. The most favorable mechanism proceeds through the following steps: the coupling reaction of the NHC catalyst with the (*E*)-3-(*p*-tolyl)acrylaldehyde (R1), [1,2]-proton transfer to form the Breslow intermediate, [1,4]-proton transfer to generate the enolate intermediate, [4+2] cycloaddition to form the stereoselective C^__^C bond, and regeneration of the NHC catalyst to give the final product. The computed results show that the additive CsHCO_3_ mainly works in proton-assisted transfer to form the Breslow and enolate intermediates *via* TS2BA and TS3BA routes and plays a key role in the [1,2] and [1,4]-proton transfer process. The [1,2]-proton transfer is a rate-determining step, while the [4+2] cycloaddition reaction is characterized as a stereoselectivity-determining step.

Also, the *Re*/*Si* pattern leads to the formation of the *RS*-configurational product, which is consistent with the experimental results.

Distortion energy is the dominant parameter determining stereoselectivity, and the *RS*-configuration product is preferentially formed. The first and fourth steps investigate the effects of electron-donating (Me) and electron-withdrawing (Cl) groups on the cinnamaldehyde. The electron-withdrawing (Cl) group on R1 and M3 has the lowest barrier energy in the first and fourth steps.

CDFT and FMO analyses have been utilized to deeply understand the role of NHC in the proposed catalytic cycle. NHC serves as a Lewis base and plays a crucial role in the polarity inversion of (*E*)-3-(*p*-tolyl)acrylaldehyde (R1).

## Data availability

The data that support the findings of this study are available within the ESI† files of this article.

## Conflicts of interest

The authors declare that they have no conflict of interest.

## Supplementary Material

RA-014-D4RA03676J-s001
