# Supplementary material for: DFT investigation of the mechanism and role of N-heterocyclic carbene (NHC) in constructing asymmetric organosilanes using NHC-catalyzed [4+2] cycloaddition reaction
Source: RSC Adv. 2024 Nov 6;14(48):35475–89. doi: 10.1039/d4ra03676j (PMC11538972; doi:10.1039/d4ra03676j)
Supplement: RA-014-D4RA03676J-s001 [file RA-014-D4RA03676J-s001.pdf]

Supplementary Information (SI) for RSC Advances.
This journal is © The Royal Society of Chemistry 2024

Supporting Information for

Mechanism and role of N-heterocyclic carbene (NHC) for constructing asymmetric organosilanes using NHC-catalyzed [4+2] cycloaddition at density functional theory

Batoul Alipour*

Department of Chemistry, Tarbiat Modares University, Tehran, Iran

*Correspondence: Batoul Alipour

Department of Chemistry, Tarbiat Modares University, P.O. Box 14115 175, Tehran, Iran.

Email: a.batoul@modares.ac.ir

Table of Contents

1. Optimized geometries of the stationary points ………………………………………….............. S1
2. Absolute single-point energies and NIMAG of the optimized structures by the M06-2X...........S6
3. The Cartesian coordinates of all the stationary points. S8

1. Optimized geometries of the stationary points

Fig S1. Optimized and geometrical parameters of all the structures of the minima- and transition states in the IEF-PCM(1,4-DO)M06-2X in conjunction with 6-31G(d,p) level.

Step 1	
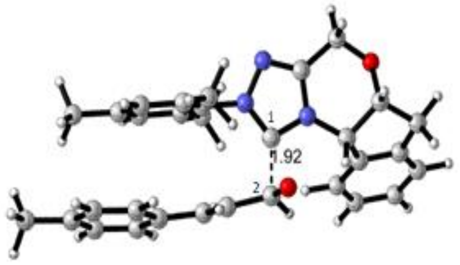	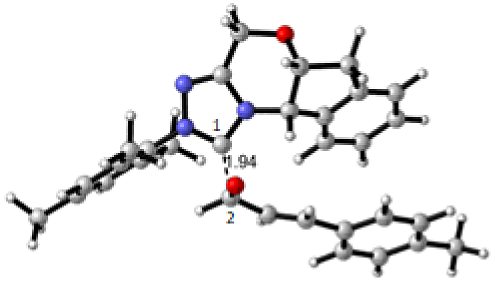
***Re*-TS1**	***Si*-TS1**
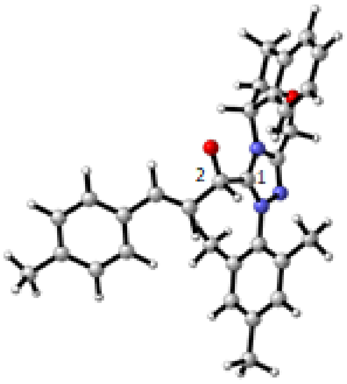	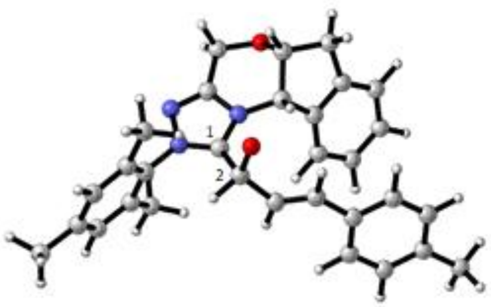
***Re*-M1**	***Si*-M1**

**Step 2**	
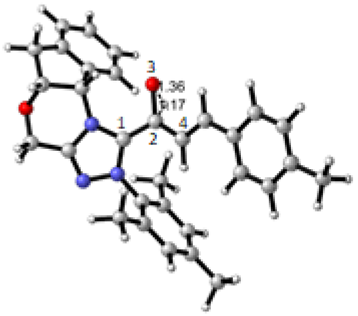	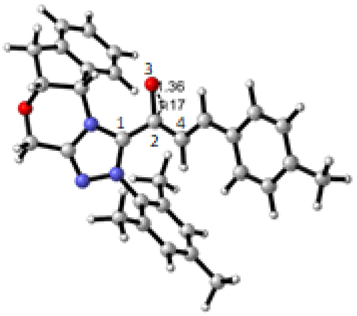
***Re*-TS2D**	***Si*-TS2D**
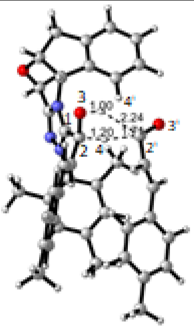	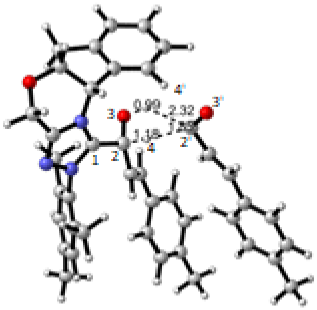
***Re*-TS2B**	***Si*-TS2B**
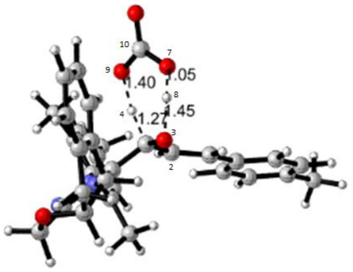	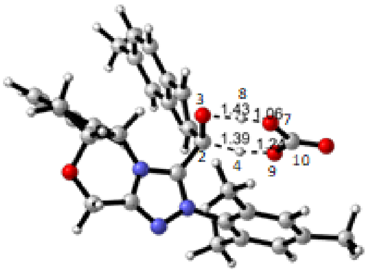
***Re*-TS2BA1**	***Si*-TS2BA1**
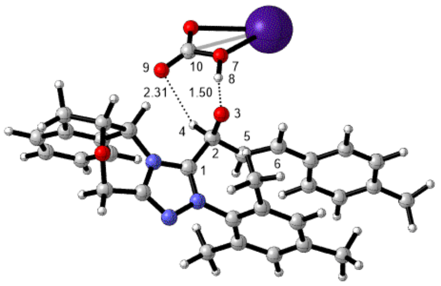	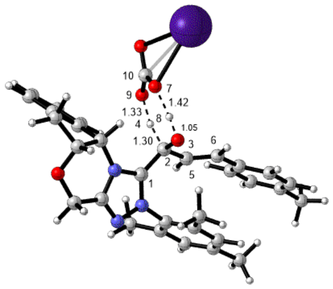
***Re*-M02BA**	***Re*-TS2BA**
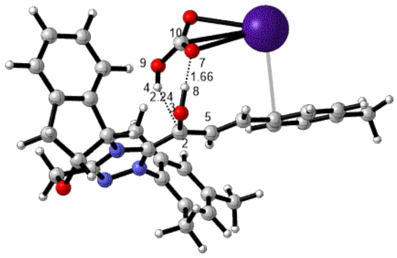	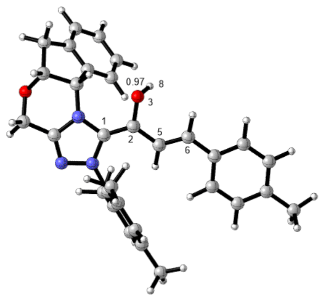

***Re*-M03BA**	***Re*-M2**
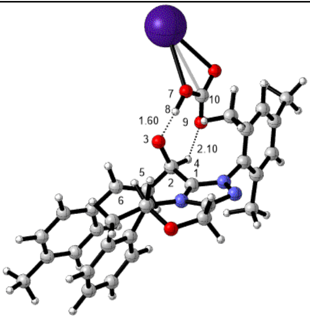	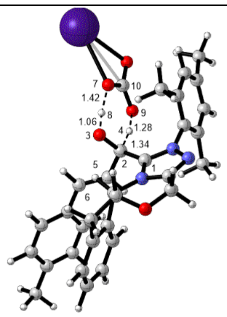
***Si*-M02BA**	***Si*-TS2BA**
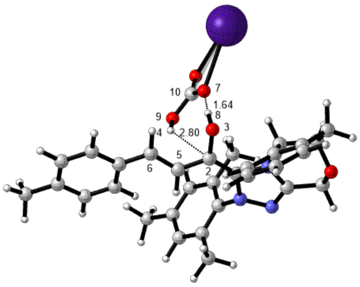	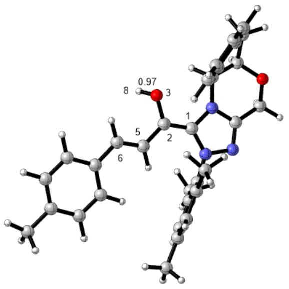
***Si*-M03BA**	***Si*-M2**
**Step 3**	
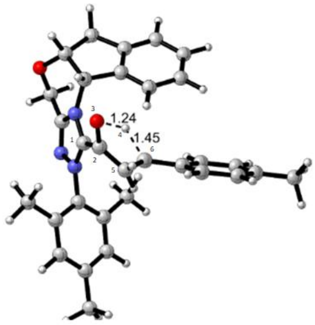	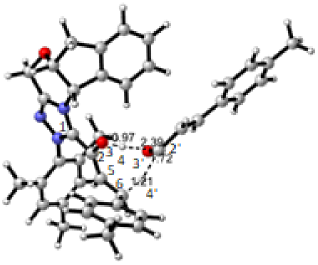
**TS3D**	**TS3B**
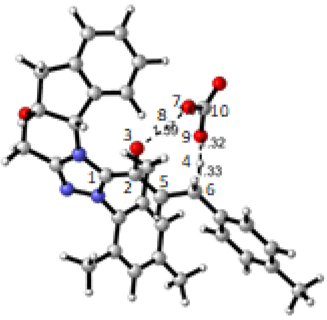	
**TS3BA1**	

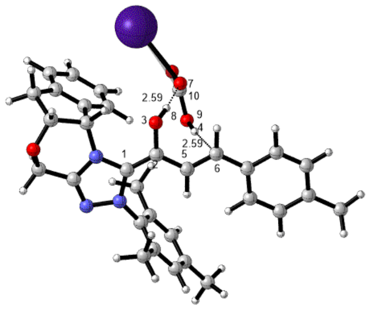	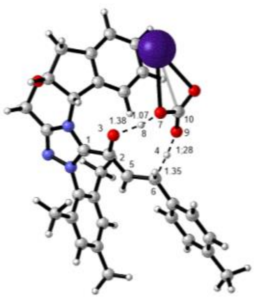
**M04BA**	**TS3BA**
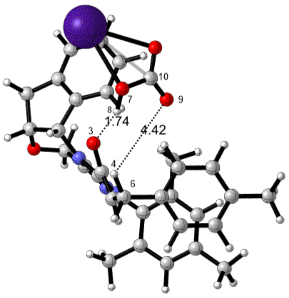	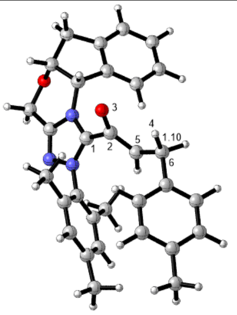
**M05BA**	**M3**
**Step 4**	
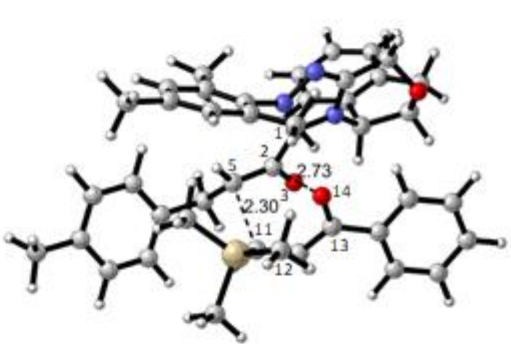	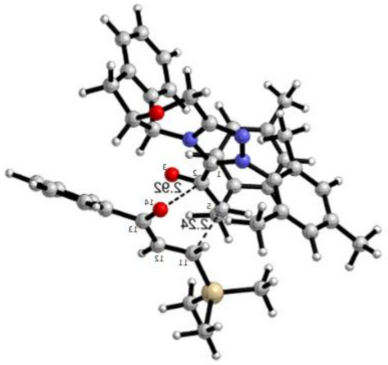
***RR*-TS4**	***RS*-TS4**
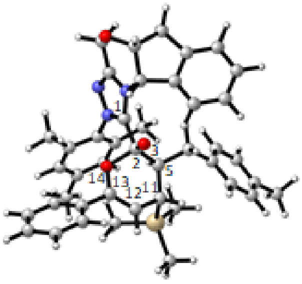	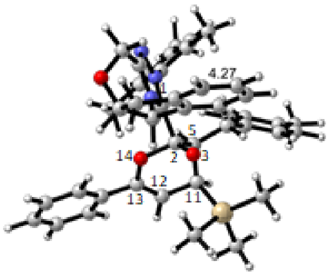
***RR*-M4**	***RS*-M4**

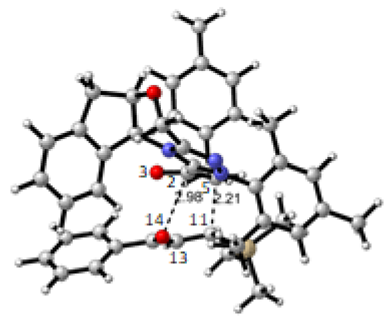	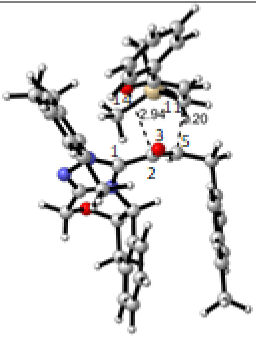
***SR*-TS4**	***SS*-TS4**
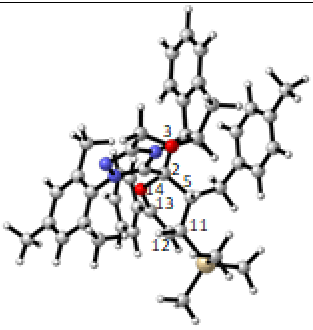	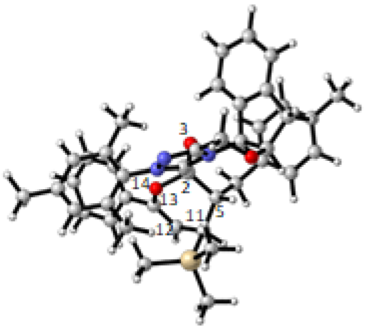
***SR*-M4**	***SS*-M4**
**Step 5**	
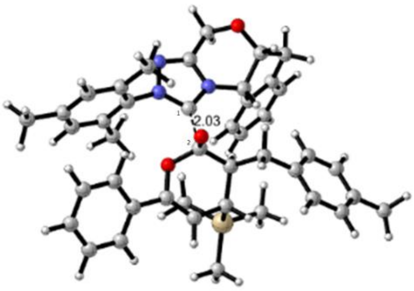	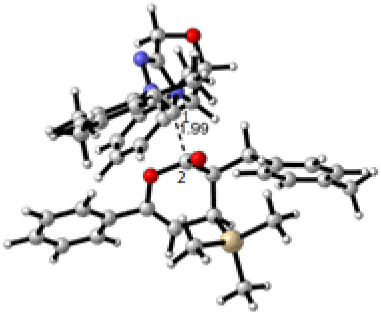
***RR*-TS5**	***RS*-TS5**
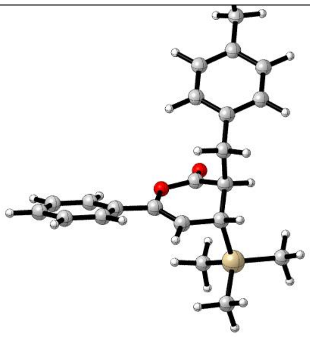	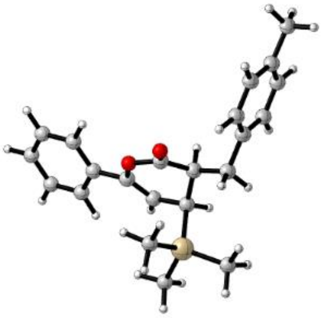
***RR*-P**	***RS*-P**

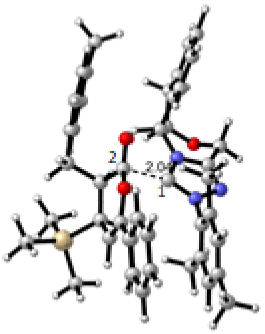	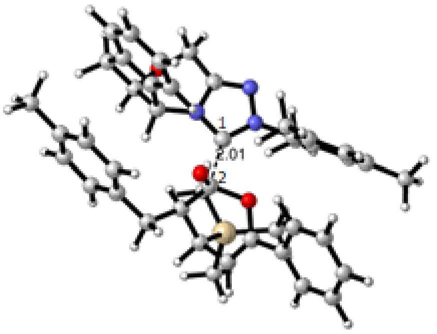
***SR*-TS5**	***SS*-TS5**
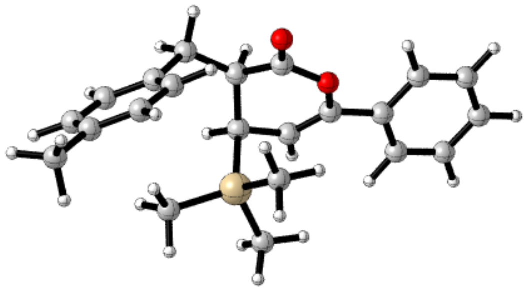	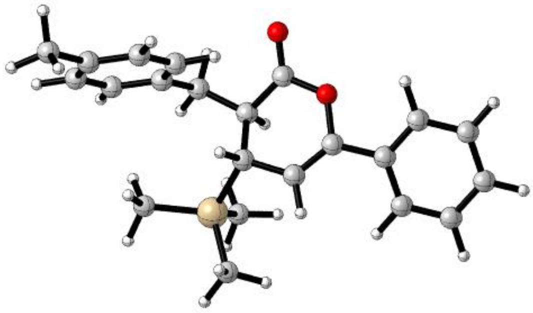
***SR*-P**	***SS*-P**

2. Absolute single-point energies and NIMAG of the optimized structures by the M06-2X

Table S1. The single point energies and NIMAGof all the structures of the minima- and transition states at IEF-PCM(1,4-DO)M06-2X in conjunction with 6-311++G(2df,2pd) (L2), level.

SR	E_{sp} (L2)	NIMAG
$CsHCO_3$	-284.323	
HCO^-_3	-264.51921	
NHC	-1052.2318	
R1	-462.24097	
R2	-831.5817554	
Si-TS1	-1514.476469	-196.92
Re-TS1	-1514.479146	-211.57
Si-M1	-1514.481116	
Re-M1	-1514.483704	
Si-TS2D	-1514.4209	-1706.99
Si-TS2B	-1976.670612	-218.76
Si-M02BA	-1798.823068	
Si-TS2BA	-1798.801701	-1282.66
Si-M03BA	-1798.852126	
Si-TS2BA1	-1778.991139	-1283.50
Re-TS2D	-1514.42	-1759.41
Re-TS2B	-1976.687883	-421.97
Re-M02BA	-1798.828328	
Re-TS2BA	-1798.811058	-1236.31
Re-M03BA	-1798.853957	
Re-TS2BA1	-1779.00565	-1022.55
Si-M2 0.494637	-1514.507885	
Re-M2 0.	-1514.509305	

TS3D	-1514.455392	-1608.73
TS3B	-1976.675029	-275.54
M04BA	-1798.857545	
TS3BA	-1798.823137	-1423.48
M05BA	-1798.854004	
TS3BA1	-1779.01542	-1370.72
M3	-1514.512784	
RR-TS4	-2346.086485	-289.65
RS-TS4	-2346.10225	-216.21
SR-TS4	-2346.095664	-230.50
SS-TS4	-2346.082434	-240.79
RR-M4	-2346.114471	
RS-M4	-2346.111115	
SR-M4	-2346.120825	
SS-M4	-2346.120796	
RR-TS5	-2346.105568	-140.07
RS-TS5	-2346.111115	-149.14
SR-TS5	-2346.114855	-148.79
SS-TS5	-2346.110119	-171.61
RR-P	-1293.890146	
RS-P	-1293.88954	
SR-P	-1293.885204	
SS-P	-1293.885896	
R1H	-422.93076	
R1Cl	-882.536	
Si-TS1H	-1475.167153	-206.05
Re-TS1H	-1475.168983	-211.57
Si-TS1Cl	-1934.773475	-204.82
Re-TS1Cl	-1934.776315	-208.68
RR-TS4H	-2306.777248	-289.34
RS-TS4H	-2306.793194	-221.08
SR-TS4H	-2306.786649	-233.22
SS-TS4H	-2306.771944	-245.25
RR-TS4Cl	-2766.383335	-295.33
RS-TS4Cl	-2766.399661	-226.79
SR-TS4Cl	-2766.39292	-244.45
SS-TS4Cl	-2766.38003	-265.02
NHC1	-1052.2276	
Re-TS1'	-1883.814368	-299.31
Si-TS1'	-1883.812557	-335.83

3. The Cartesian coordinates of all the stationary points.

$CsHCO_3$

Zero-point correction= 0.028768 (Hartree/Particle)

C	-1.99826700	0.04290800	-0.00009400
O	-1.42852100	1.15178100	-0.00022600
O	-3.37501600	0.07419800	0.00025600
H	-3.62938100	-0.85627000	0.00071000
O	-1.47425100	-1.10416700	-0.00029100
Cs	1.19711400	-0.00683100	0.00003500

HCO_3

Zero-point correction= 0.027102 (Hartree/Particle)

C	-1.99826700	0.04290800	-0.00009400
O	-1.42852100	1.15178100	-0.00022600
O	-3.37501600	0.07419800	0.00025600
H	-3.62938100	-0.85627000	0.00071000
O	-1.47425100	-1.10416700	-0.00029100

CsCl

R1

Zero-point correction= 0.172398 (Hartree/Particle)

C	-0.67169200	1.29215000	-0.00145500
C	-1.93597900	-1.17317100	-0.00394600
C	-2.06082300	1.22375700	-0.00316800
C	0.10693500	0.12955100	-0.00071000
C	-0.55273800	-1.11031900	-0.00214300
C	-2.71551200	-0.00779400	-0.00345000
C	1.56368700	0.25612500	-0.00006000
C	2.47285700	-0.73253200	0.00100100

C	3.91221100	-0.41085900	0.00178600
O	4.36320900	0.71683400	0.00159000
H	4.59469900	-1.28314300	0.00259200
C	-4.21846400	-0.09786100	0.00637100
H	-0.17812200	2.26011500	-0.00200100
H	-2.42962900	-2.14140400	-0.00659500
H	-2.64499500	2.13953100	-0.00488900
H	0.02197100	-2.03114400	-0.00341600
H	1.95715000	1.27267800	-0.00023600
H	2.19936700	-1.78339300	0.00136700
H	-4.57583100	-0.52356800	0.94919500
H	-4.67579000	0.88593000	-0.11483600
H	-4.57737800	-0.74455400	-0.79926100

R2

Zero-point correction= 0.247353 (Hartree/Particle)

C	-1.53807300	-0.72886900	0.15207300
H	-1.52128600	-1.78427300	0.43552100
C	-0.33762200	-0.16980500	-0.04676300
H	-0.24685800	0.87185300	-0.34365700
C	0.91638200	-0.97362900	0.10151800
O	0.86793600	-2.18158300	0.25802400
C	2.24116100	-0.27593900	0.03702500
C	3.38181700	-1.06344900	-0.15385600
C	2.37856000	1.10737100	0.18520300
C	4.63847400	-0.47670000	-0.21355900
H	3.25501900	-2.13609300	-0.25420100
C	3.64049100	1.69362200	0.13774100
H	1.51079100	1.73348000	0.36226100

C	4.76889000	0.90461300	-0.06796300
H	5.51805500	-1.09220300	-0.37095700
H	3.74183800	2.76658900	0.26237600
H	5.75089500	1.36471300	-0.11164800
Si	-3.17547900	0.17896800	-0.02943200
C	-4.19497500	-0.67960000	-1.35652500
H	-5.16819000	-0.19184900	-1.46882200
H	-3.68744800	-0.64969600	-2.32464700
H	-4.37209500	-1.72813100	-1.10032100
C	-2.83381400	1.96246500	-0.51697600
H	-3.77364300	2.51154000	-0.62858600
H	-2.23638900	2.47695800	0.24180500
H	-2.29902800	2.02141600	-1.46973000
C	-4.08358600	0.11345900	1.61653000
H	-3.51145900	0.61316400	2.40318400
H	-5.05641800	0.60860900	1.53890400
H	-4.25679800	-0.92018500	1.92968100

NHC

Zero-point correction= 0.383831 (Hartree/Particle)

C	-2.23701500	1.94392100	0.29257000
C	-4.85547000	1.53799700	-0.65623700
C	-2.93340000	2.97897100	-0.33283200
C	-2.85888800	0.70880900	0.42160400
C	-4.15993400	0.50520300	-0.04096800
C	-4.23023100	2.77687200	-0.80348400
C	-4.60834800	-0.90983900	0.24715700
C	-3.28780000	-1.65010600	0.48012900
C	-2.34759900	-0.56422000	1.06863000

N	-0.96146100	-0.91068900	0.81779900
C	-0.56780400	-1.75219000	-0.19107200
C	-1.57415300	-2.70264300	-0.75304400
O	-2.83612200	-2.06829900	-0.79770400
N	0.66878100	-1.63329000	-0.54135600
N	1.19070800	-0.60899300	0.25207000
C	0.18036800	-0.14810500	1.10421700
C	2.49761900	-0.13764300	0.09446600
C	5.12978000	0.82338400	-0.17569600
C	3.55782100	-1.04854800	-0.10138600
C	2.75110000	1.24824100	0.16944600
C	4.06438600	1.69402900	0.04406000
C	4.84525800	-0.53982400	-0.25117700
C	3.34561500	-2.54036500	-0.10124000
C	1.63612000	2.25021900	0.32407500
C	6.54274500	1.33443600	-0.29224700
H	-1.22798900	2.09856000	0.66248100
H	-5.86454000	1.38307700	-1.02695300
H	-2.46125400	3.94850500	-0.45464200
H	-4.75722800	3.59038200	-1.29186000
H	-5.23053900	-0.94767900	1.14872300
H	-5.16376500	-1.37450000	-0.57091600
H	-3.37364700	-2.51897000	1.14720400
H	-2.49621400	-0.51790500	2.15637200
H	-1.62126400	-3.61099100	-0.13347200
H	-1.29898100	-2.97884700	-1.77124400
H	4.25416500	2.76442700	0.09503300
H	5.66077400	-1.24363300	-0.40572000

H	4.30468000	-3.05030700	0.01384800
H	2.86936800	-2.88623500	-1.02122700
H	2.68945300	-2.83946000	0.72149600
H	2.02641100	3.26440500	0.21543200
H	0.86016700	2.09538200	-0.43246200
H	1.14947200	2.18230300	1.30528000
H	7.02696900	1.38936500	0.68899100
H	6.56537700	2.33828700	-0.72450800
H	7.15034800	0.67942400	-0.92164700

Si-TS1 -210.01

Zero-point correction= 0.558357 (Hartree/Particle)

C	4.03690000	-3.34670000	-0.43797200
C	2.82914200	-2.97888500	0.14024600
C	2.76976600	-1.93751500	1.07536500
C	3.96563200	-1.30175000	1.42623300
C	5.17177500	-1.66950400	0.84166000
C	5.22891300	-2.69633600	-0.10243100
H	4.06021700	-4.15700800	-1.16287900
H	1.92071500	-3.50814600	-0.13352800
H	3.93863600	-0.48312700	2.14120400
H	6.08389800	-1.14529000	1.11557000
C	1.50422000	-1.42995200	1.62348300
H	1.56288100	-0.80496900	2.51467200
C	0.29848500	-1.56857700	1.06741800
C	-0.90572400	-0.86891400	1.63215000
H	0.13879800	-2.14740800	0.15813500
O	-0.79997200	-0.09929300	2.61887500
H	-1.83871000	-1.45652300	1.49316600

C	2.03194400	0.28760900	-1.12749100
C	4.06803300	2.21014400	-0.84823200
C	3.24932000	0.13153900	-1.78938000
C	1.85308000	1.40236500	-0.32164000
C	2.85288500	2.36855700	-0.19484400
C	4.26197000	1.07892400	-1.64122600
C	2.38825300	3.49766800	0.69817600
C	0.86928100	3.30910500	0.72013800
C	0.68803400	1.78302300	0.56715100
N	-0.64603000	1.49740200	0.05095800
C	-1.49227100	2.45345400	-0.44437300
C	-1.04723700	3.88292800	-0.53617500
O	0.35491400	3.96436400	-0.43355200
N	-2.66444800	1.95254100	-0.69789700
N	-2.52250700	0.63253000	-0.32880500
C	-1.31320700	0.32043700	0.14364200
C	-3.65810700	-0.24265900	-0.34559800
C	-5.84913000	-1.93252800	-0.34206500
C	-4.45627600	-0.29230400	0.80022800
C	-3.91351600	-1.00092500	-1.48811200
C	-5.02361000	-1.84365500	-1.46435100
C	-5.55393300	-1.15132000	0.77655000
C	-4.13217200	0.56055300	1.99861300
C	-3.01243200	-0.89720400	-2.68923700
C	-7.02395200	-2.87648000	-0.32915000
H	1.24667300	-0.45625900	-1.22524600
H	4.84986800	2.95790500	-0.75136600
H	3.41640400	-0.74497600	-2.40721400

H	5.20807800	0.93778000	-2.15455600
H	2.79343500	3.39801300	1.71161300
H	2.64677600	4.49081500	0.32279900
H	0.38126300	3.69985500	1.62301000
H	0.74272700	1.28185600	1.54250100
H	-1.53571400	4.45623100	0.26567500
H	-1.33584900	4.31369500	-1.49635700
H	-5.25018800	-2.44294300	-2.34288500
H	-6.19450200	-1.20867600	1.65317100
H	-4.86367300	0.39649200	2.79176000
H	-4.15203200	1.62096800	1.72638200
H	-3.13106200	0.34659100	2.39401100
H	-3.37092100	-1.53418700	-3.49930800
H	-2.96381500	0.13404600	-3.05135300
H	-1.99073200	-1.19924100	-2.43701700
H	-7.44878100	-2.99361200	-1.32876300
H	-7.80977000	-2.52004500	0.34051500
H	-6.71723400	-3.86837000	0.01848200
C	6.53953500	-3.11432600	-0.71738300
H	7.23763700	-2.27494400	-0.76697400
H	7.01593600	-3.90435400	-0.12699900
H	6.39678700	-3.50266500	-1.72895900

Re-TS1 -213.28

Zero-point correction= 0.558625 (Hartree/Particle)

C	-3.61071400	-1.70114800	1.22344800
C	-6.38925100	-1.60391000	0.77901900
C	-4.48705900	-2.39429700	2.05874000
C	-4.14190100	-0.96747800	0.17223300

C	-5.51809600	-0.90857600	-0.04925700
C	-5.86285000	-2.34934100	1.83451000
C	-5.82385700	-0.02215900	-1.23562400
C	-4.53813400	0.79558900	-1.39551400
C	-3.42711400	-0.15170000	-0.88244000
N	-2.29925100	0.62676700	-0.38732300
C	-2.29681000	1.98722700	-0.25195200
C	-3.53306800	2.76909600	-0.57753700
O	-4.65519400	1.91681800	-0.52898500
N	-1.12724800	2.41368500	0.12031900
N	-0.38510400	1.25562500	0.20989900
C	-1.06618100	0.14713800	-0.09783300
C	1.00658200	1.34232300	0.55344500
C	3.69450600	1.50268500	1.19515400
C	1.91973500	1.59916600	-0.46936100
C	1.38434100	1.15306800	1.88325400
C	2.74294100	1.23645100	2.18246900
C	3.26733200	1.67942900	-0.12033200
C	1.44856800	1.73630200	-1.89179400
C	0.35191900	0.85639700	2.93820900
C	5.15470300	1.61279500	1.55071600
H	-2.53827700	-1.72213800	1.40108700
H	-7.46228500	-1.55853300	0.61800200
H	-4.09593000	-2.97014300	2.89120500
H	-6.53149600	-2.89301800	2.49431700
H	-6.00652300	-0.61729500	-2.13736200
H	-6.67998300	0.63941000	-1.08325200
H	-4.35134100	1.13564200	-2.42265000

H	-3.03173100	-0.77643000	-1.69460400
H	-3.42551700	3.22291200	-1.57361500
H	-3.68578300	3.56348300	0.15421900
H	3.06610700	1.08853800	3.21049100
H	4.00400100	1.85315600	-0.90084300
H	2.30065000	1.79275000	-2.57255100
H	0.84558600	2.64201500	-2.01618700
H	0.82647600	0.87919800	-2.17870800
H	-0.42589300	1.62588100	2.95226200
H	-0.14448100	-0.10057700	2.74346500
H	0.81157300	0.80895100	3.92688000
H	5.44201000	0.84209100	2.27199800
H	5.37289200	2.58717800	2.00069300
H	5.78215700	1.49731500	0.66344100
C	-0.56697400	-1.44763100	-1.05117300
H	-1.28192400	-2.10956400	-0.51346800
C	0.83476900	-1.60715000	-0.53566000
H	0.95089700	-1.68666400	0.54471700
C	1.87748600	-1.57905700	-1.36736000
H	1.65386500	-1.45945500	-2.42693700
C	3.29256100	-1.55985800	-0.97512400
C	4.24844300	-1.12573600	-1.89767000
C	3.72686200	-1.87351800	0.32145200
C	5.58383500	-0.97271800	-1.53266500
H	3.93377300	-0.88127700	-2.90933400
C	5.05721300	-1.72639600	0.67942900
H	3.01282700	-2.23264400	1.05673200
C	6.01034100	-1.26467900	-0.23795900

H	6.30510400	-0.62063700	-2.26587900
H	5.37107200	-1.97232600	1.69162700
C	7.44791600	-1.09050300	0.17738800
H	7.53780900	-0.33990400	0.97029000
H	7.86245500	-2.02505800	0.56716900
H	8.06747400	-0.76928300	-0.66285100
O	-0.78638300	-1.16018900	-2.25624900

Si-M1

Zero-point correction= 0.560008 (Hartree/Particle)

C	-4.00087500	-3.43341700	0.58097500
C	-2.77313400	-3.00907300	0.08738900
C	-2.70036500	-2.04478400	-0.92566100
C	-3.90292800	-1.54376600	-1.43892000
C	-5.12871600	-1.96730900	-0.94051700
C	-5.20023900	-2.91893400	0.07961400
H	-4.03305900	-4.18278000	1.36834900
H	-1.85828500	-3.43600600	0.48891600
H	-3.86746900	-0.78963700	-2.22113400
H	-6.04759100	-1.55097600	-1.34606600
C	-1.42872800	-1.48221400	-1.40356100
H	-1.43631900	-0.92708200	-2.34157400
C	-0.26950300	-1.49340800	-0.74497800
C	-6.53161800	-3.39917800	0.59772600
H	-6.95047200	-4.17243100	-0.05498500
H	-6.43650700	-3.82785500	1.59824500
H	-7.25624100	-2.58179400	0.64213700
C	0.94089100	-0.74640000	-1.28708900
H	-0.15449000	-1.99993500	0.21568800

O	0.79007300	-0.09242300	-2.43167100
H	1.80435000	-1.45614200	-1.23186500
C	-2.18724800	0.46757200	1.08661600
C	-4.10123800	2.47755800	0.63510300
C	-3.44165300	0.40114900	1.69315200
C	-1.91046000	1.53209800	0.24138200
C	-2.85162200	2.54465000	0.03514700
C	-4.39459500	1.39063900	1.45995600
C	-2.27922900	3.62467400	-0.85645900
C	-0.77559100	3.36589300	-0.75354900
C	-0.68905300	1.83298100	-0.60541500
N	0.61782300	1.48812200	-0.04562100
C	1.45014000	2.39006500	0.55771100
C	1.02630700	3.82034600	0.72713300
O	-0.34604200	3.97276600	0.46222200
N	2.62151800	1.87451400	0.78957000
N	2.51722300	0.60078500	0.28648600
C	1.32465700	0.35530100	-0.25273200
C	3.65756900	-0.27220700	0.28384900
C	5.85182800	-1.94967100	0.26431200
C	4.42211600	-0.34332300	-0.88390600
C	3.94236800	-1.00107500	1.43796800
C	5.05532700	-1.83975200	1.40487600
C	5.52057500	-1.20065200	-0.86710600
C	4.05914000	0.47539900	-2.09441300
C	3.06760800	-0.88012000	2.65686200
C	7.06594200	-2.84235700	0.25951400
H	-1.45156200	-0.30926700	1.26497600

H	-4.83401300	3.26329400	0.47630800
H	-3.67961700	-0.43915600	2.33748000
H	-5.36880500	1.32110100	1.93354600
H	-2.60836300	3.50641500	-1.89510000
H	-2.52290000	4.63858200	-0.53065900
H	-0.18705200	3.74363300	-1.59975900
H	-0.71270600	1.32224200	-1.57818800
H	1.63501100	4.43492200	0.04787900
H	1.20450300	4.15628600	1.75029300
H	5.30221500	-2.42309800	2.28833400
H	6.13261600	-1.28431000	-1.76176100
H	4.76414000	0.28427600	-2.90522200
H	4.09932300	1.54322100	-1.85214400
H	3.04036900	0.25964300	-2.44656400
H	3.42891800	-1.52673700	3.45774200
H	3.04850700	0.15061800	3.02299100
H	2.03455200	-1.16245000	2.42725500
H	6.97193300	-3.64174800	0.99766000
H	7.96610100	-2.26872800	0.50285400
H	7.21866700	-3.29489900	-0.72295400

Re-M1

Zero-point correction= 0.559256 (Hartree/Particle)

C	3.73441200	-0.25639400	-1.78699100
C	6.16579100	-1.38923100	-0.92033100
C	4.88832500	-0.14499300	-2.56202800
C	3.82373800	-0.91342000	-0.56785200
C	5.02104100	-1.48690000	-0.14000100
C	6.09266700	-0.70406000	-2.13278500

C	4.82629900	-2.18453300	1.18680600
C	3.56023500	-1.51876100	1.72825700
C	2.74328000	-1.19902400	0.45287100
N	1.82485500	-0.07165400	0.72368100
C	1.93463100	0.73011900	1.83098800
C	2.94107800	0.43026800	2.89758500
O	3.99751000	-0.31863100	2.35822600
N	1.03602800	1.66856200	1.83356700
N	0.33056600	1.43940500	0.68482100
C	0.78097600	0.38479700	0.00029500
C	-0.76850200	2.29417400	0.33540900
C	-2.90364900	3.90578800	-0.36514900
C	-1.98975500	2.09631900	0.98657900
C	-0.56395200	3.27947700	-0.63080700
C	-1.65758600	4.07713200	-0.96824800
C	-3.04984900	2.91894200	0.61407200
C	-2.14306400	1.01558700	2.02238600
C	0.78792800	3.47856200	-1.26399200
H	2.77766200	0.11768400	-2.13453600
H	7.10133500	-1.83117600	-0.58996100
H	4.84589500	0.37064600	-3.51612500
H	6.97973400	-0.61080600	-2.75160800
H	4.65883100	-3.25803100	1.04428300
H	5.65393700	-2.05477100	1.88804900
H	2.99760900	-2.13151300	2.44545500
H	2.11363200	-2.02447200	0.11701800
H	2.44303000	-0.11424500	3.71420000
H	3.34903100	1.35994000	3.29696800

H	-1.52902200	4.85304800	-1.71842100
H	-4.01389900	2.78567500	1.09903100
H	-3.16685200	0.98454300	2.39882300
H	-1.46479900	1.18286800	2.86407900
H	-1.90539200	0.03561700	1.59122400
H	0.78755700	4.36937000	-1.89362000
H	1.56299500	3.59458400	-0.50053600
H	1.06560900	2.62240400	-1.88747100
C	0.22807600	-0.31441100	-1.29986000
H	0.25361600	0.52365900	-2.05480700
C	-1.26583700	-0.53753700	-1.00512500
H	-1.91666400	0.33466900	-0.96501200
C	-1.73617300	-1.77554300	-0.85107300
H	-1.00989300	-2.57808400	-0.97422900
C	-3.12978000	-2.14819300	-0.56093500
C	-3.50858400	-3.49409000	-0.61982100
C	-4.11079400	-1.21095000	-0.20370900
C	-4.81399800	-3.88928000	-0.34742100
H	-2.76287300	-4.23904700	-0.88519800
C	-5.41237900	-1.60894400	0.06850000
H	-3.84977000	-0.15749800	-0.13021300
C	-5.79034300	-2.95414500	-0.00202700
H	-5.07917600	-4.94227300	-0.40093100
H	-6.15389400	-0.86381000	0.34734400
C	-7.21261900	-3.37418100	0.26522600
H	-7.25895300	-4.39866000	0.64250300
H	-7.68836900	-2.71726900	0.99783900
H	-7.81149500	-3.33326100	-0.65085900

O	0.93665300	-1.38286400	-1.57348800
C	-4.07799200	4.75486700	-0.77695700
H	-3.75098800	5.73086500	-1.14197000
H	-4.63671200	4.26900400	-1.58330100
H	-4.76687800	4.90947600	0.05642900

Si-TS2D -1706.99

Zero-point correction= 0.555256 (Hartree/Particle)

C	3.92943048	-3.57573988	-0.64507720
C	2.72409248	-3.06886788	-0.17593020
C	2.69288348	-2.13656788	0.87038880
C	3.91461548	-1.75127288	1.43568980
C	5.11884948	-2.25870888	0.96202180
C	5.14897148	-3.17999388	-0.08682920
H	3.92714648	-4.29793788	-1.45812120
H	1.79286048	-3.40934788	-0.62018820
H	3.91425948	-1.02516088	2.24443680
H	6.05340348	-1.93204288	1.41155780
C	1.45353548	-1.50503488	1.34207480
H	1.48078048	-0.96505288	2.28816180
C	0.30304148	-1.43287788	0.66470080
C	6.45193248	-3.75655688	-0.57796020
H	6.72044048	-4.65591888	-0.01348920
H	6.38942948	-4.03748488	-1.63235420
H	7.26943448	-3.04052388	-0.46187020
C	-0.82448052	-0.61673688	1.19903480
H	0.17505848	-1.90277788	-0.31178120
O	-0.68779452	0.01571012	2.39998180
H	-0.98424396	-1.34812419	2.18719775

C	2.27300048	0.68501412	-0.94496420
C	4.02214948	2.83905212	-0.48965720
C	3.56373048	0.67481512	-1.47474120
C	1.87702548	1.76758712	-0.17232020
C	2.73900348	2.84785412	0.03869580
C	4.43391848	1.73791612	-1.24208020
C	2.05596448	3.91914012	0.85988380
C	0.57925348	3.55457212	0.70775580
C	0.59689948	2.01586312	0.59972080
N	-0.65993152	1.58636212	-0.01602320
C	-1.53380752	2.41563312	-0.66068220
C	-1.19228752	3.86451512	-0.85081820
O	0.15912948	4.10065612	-0.54190020
N	-2.66280552	1.81577512	-0.90731320
N	-2.48937852	0.56499412	-0.36973420
C	-1.29710252	0.41719512	0.20331680
C	-3.58041752	-0.36537588	-0.30686420
C	-5.67961552	-2.15233688	-0.15120020
C	-4.35575852	-0.37190688	0.85675280
C	-3.81167852	-1.20661588	-1.39265820
C	-4.87761152	-2.10003888	-1.29153420
C	-5.40500552	-1.28588588	0.91015280
C	-2.93390152	-1.14209488	-2.61377620
C	-6.84074652	-3.10917888	-0.06930120
H	1.60218248	-0.14621088	-1.12861320
H	4.69206748	3.67861412	-0.32802020
H	3.89092148	-0.17748888	-2.06198020
H	5.43641848	1.71408112	-1.65743820

H	2.34839548	3.86410912	1.91451480
H	2.24339548	4.93420112	0.50180480
H	-0.06731552	3.91537212	1.51818280
H	0.59019048	1.51109712	1.58314380
H	-1.85896752	4.45744012	-0.20772720
H	-1.35221352	4.16696912	-1.88741420
H	-5.08248452	-2.77160388	-2.12137220
H	-6.02448452	-1.32140788	1.80291380
H	-3.27330552	-1.84990688	-3.37130420
H	-2.94218452	-0.13760388	-3.04727420
H	-1.89482752	-1.37983188	-2.36331220
H	-6.74556452	-3.91132188	-0.80394720
H	-7.78397252	-2.58820888	-0.26256220
H	-6.91150452	-3.55763788	0.92466580
C	-4.05608045	0.60336107	2.01028887
H	-3.26056190	0.21286704	2.60992597
H	-3.76755072	1.55241406	1.60910435
H	-4.93191097	0.72242307	2.61331883

Re-TS2D -1759.41

Zero-point correction= 0.555486 (Hartree/Particle)

C	-3.61663600	-0.85126900	1.79468600
C	-6.15575900	-1.74921300	0.97118700
C	-4.63139400	-1.06544000	2.72725600
C	-3.89298000	-1.07821100	0.45523400
C	-5.15020900	-1.52022100	0.04159500
C	-5.88711500	-1.51603500	2.32023700
C	-5.18283200	-1.67191400	-1.46298200
C	-4.00832100	-0.79501600	-1.90307400

C	-2.98993700	-0.96168300	-0.75357200
N	-2.06401600	0.18699700	-0.74134500
C	-2.33066500	1.36144100	-1.39706800
C	-3.53654300	1.48760300	-2.27815700
O	-4.50740400	0.53836400	-1.92290200
N	-1.37689800	2.22683100	-1.24589500
N	-0.45766000	1.57101500	-0.46722100
C	-0.84311300	0.31861800	-0.15850200
C	0.73221700	2.25231100	-0.05066500
C	3.04769200	3.52578500	0.76925600
C	1.79525900	2.34336900	-0.95284900
C	0.78103400	2.77122300	1.24339400
C	1.95825100	3.40929400	1.63274700
C	2.94878700	2.99114100	-0.51832500
C	1.70535900	1.69398700	-2.30649100
C	-0.38970700	2.61601600	2.17597700
H	-2.63598600	-0.51156200	2.10817100
H	-7.13686700	-2.08987800	0.65369100
H	-4.44005100	-0.88517800	3.78009900
H	-6.66426600	-1.67975100	3.06003600
H	-5.01151800	-2.71220800	-1.76216900
H	-6.11198000	-1.32815700	-1.92367300
H	-3.58916900	-1.05522700	-2.88453000
H	-2.37432200	-1.85590200	-0.85096000
H	-3.21789500	1.35638000	-3.32357500
H	-3.97725200	2.47992900	-2.17181000
H	2.02540300	3.82267400	2.63588100
H	3.79370500	3.07272400	-1.19785600

H	2.59026800	1.91978000	-2.90341300
H	0.81844800	2.03006800	-2.85048800
H	1.63472900	0.60485300	-2.19446600
H	-0.21180300	3.14546900	3.11289700
H	-1.30738800	3.00489400	1.72407800
H	-0.55485300	1.55659200	2.40235100
C	-0.16115100	-0.70878700	0.64069800
H	-0.07181192	-0.95703877	1.85397776
C	1.31295100	-0.77793200	0.44233300
H	1.88420300	0.14595200	0.42940200
C	1.92104900	-1.96471500	0.32373900
H	1.28794500	-2.84650900	0.39414600
C	3.36215600	-2.17106600	0.12061000
C	3.89136300	-3.46358500	0.20949500
C	4.25173800	-1.12663900	-0.18087000
C	5.24923500	-3.70493000	0.02286700
H	3.22221300	-4.29094200	0.43095900
C	5.60474900	-1.37084300	-0.36426700
H	3.87762300	-0.11092600	-0.28615500
C	6.13189800	-2.66387100	-0.26279900
H	5.62968200	-4.72046600	0.09948600
H	6.27084100	-0.54403300	-0.60058700
O	-0.86190800	-1.91407600	0.74630500
C	4.32230800	4.18687000	1.22503800
H	4.12842100	4.92670600	2.00457500
H	5.01324400	3.44409600	1.63644900
H	4.82785200	4.68424300	0.39426100
C	7.63968390	-2.91658740	-0.44804506

H	7.96930036	-2.45745903	-1.35659012
H	8.17563200	-2.49791934	0.37801589
H	7.82173637	-3.96997245	-0.49427081

Si-TS2B -218.76

Zero-point correction= 0.730760 (Hartree/Particle)

C	4.12876200	2.35174400	0.69662400
C	2.79233700	1.97261900	0.64357000
C	2.29969300	0.96040100	1.47934500
C	3.18923100	0.37487700	2.39180900
C	4.52446300	0.75747300	2.44003800
C	5.02093500	1.74881700	1.58835200
H	4.48895800	3.13455800	0.03341100
H	2.11934800	2.47158900	-0.04953800
H	2.82760100	-0.40868900	3.05272500
H	5.19592200	0.27637700	3.14716100
C	0.92351800	0.45446200	1.38842800
H	0.55085000	-0.13704600	2.22280300
C	0.14325300	0.56227100	0.30550700
C	-1.13308900	-0.19127400	0.18488500
H	0.50821100	1.07756100	-0.57960100
O	-1.43459400	-1.04078600	1.17564800
H	-0.71670000	-1.20341000	-0.67155500
C	-4.12302100	0.98421000	-1.51439800
N	-3.41534900	2.06109900	-1.59341500
N	-3.48698200	-0.01898500	-0.81489200
N	-2.26113800	1.74129200	-0.93016100
C	-2.25659800	0.46829600	-0.46582100
C	-1.33373500	2.80048300	-0.64641100

C	-0.56692900	3.30806300	-1.69667200
C	-1.23830900	3.26771700	0.66725700
C	0.35418300	4.30909700	-1.39198400
C	-0.29387300	4.26011500	0.92202200
C	0.51568300	4.78216900	-0.08810600
C	-5.52495500	0.76585500	-1.97033100
C	-4.24363600	-1.30675200	-0.72809000
C	-5.71851300	-0.94527200	-0.34616400
O	-6.23989900	0.26354300	-0.86288400
C	-5.70460700	-0.92419600	1.18814300
H	-5.32171100	0.04604100	1.52819100
H	-6.69957200	-1.06711200	1.61488000
C	-4.74602600	-2.04814100	1.48070100
C	-4.66360300	-2.87296200	2.59428500
C	-3.92016900	-2.27681800	0.37784900
C	-3.76362700	-3.93863000	2.57195500
H	-5.30278800	-2.70661300	3.45603000
C	-3.06527800	-3.36783200	0.32264400
C	-2.98586600	-4.19414900	1.44201400
H	-3.68844200	-4.59391800	3.43413600
H	-2.45772600	-3.55890100	-0.55796600
H	-2.31304500	-5.04538000	1.43062600
H	-5.55997600	0.07544600	-2.82476300
H	-5.96951800	1.71679400	-2.26236900
H	-0.18256600	4.62836100	1.93870400
H	0.97240700	4.71419200	-2.18911800
C	-2.09158500	2.69399600	1.76602300
H	-3.13724000	2.61250100	1.45254100

H	-1.74561500	1.69188900	2.04554000
H	-2.04712700	3.32882800	2.65218500
C	-0.71045800	2.76024400	-3.09098500
H	-0.57011400	1.67390000	-3.09935300
H	-1.70839500	2.96102200	-3.49034600
H	0.02911900	3.20647600	-3.75718000
C	1.58088700	5.79529300	0.23842200
H	1.23494900	6.50608500	0.99256100
H	2.46489600	5.29003300	0.64334600
H	1.88794100	6.35221900	-0.64921800
C	5.67252500	-1.59919900	-0.36988300
C	4.29360600	-1.72188100	-0.45827900
C	3.71309900	-2.79438100	-1.15331500
C	4.56566200	-3.71469700	-1.77430600
C	5.94737600	-3.58765900	-1.68020300
C	6.52473300	-2.52859000	-0.97658300
H	6.09379700	-0.75886700	0.17739200
H	3.66352000	-0.96508600	0.00128800
H	4.13562700	-4.54746000	-2.32464200
H	6.58925900	-4.32127100	-2.16109800
C	2.25686500	-2.96660500	-1.24472300
H	1.88012400	-3.61539700	-2.03582300
C	1.35721700	-2.39312200	-0.43445700
H	1.67470100	-1.75989800	0.38772600
C	-0.12360300	-2.53759300	-0.65208200
H	-0.92940700	-2.05610600	0.60242100
O	-0.53529300	-3.37972200	-1.45130300
H	-6.32886100	-1.78690800	-0.69943900

H	-4.16342500	-1.76462400	-1.71794600
C	6.47332800	2.14960900	1.63775700
H	6.67125900	3.00079500	0.98272400
H	6.77287300	2.42459400	2.65324900
H	7.12240200	1.32612100	1.32112100
C	8.02096200	-2.36615700	-0.90405400
H	8.38302600	-1.71859700	-1.70974700
H	8.32337800	-1.91139900	0.04272900
H	8.52959700	-3.32823200	-1.00091300

Re-TS2B -421.97

Zero-point correction= 0.730323 (Hartree/Particle)

C	4.57315100	2.69869500	-0.11646700
C	3.26243300	2.24593900	-0.13798300
C	2.40571100	2.56112900	-1.21377100
C	2.91930800	3.36259100	-2.23813700
C	4.23593500	3.81421800	-2.21193200
C	5.08771900	3.48317300	-1.15952100
H	5.21504500	2.43586100	0.72161900
H	2.90238300	1.63208800	0.68490200
H	2.27473500	3.62257900	-3.07040900
H	4.60702000	4.42318200	-3.02439200
C	1.00915100	2.10292400	-1.29261400
H	0.41438500	2.49880500	-2.11269500
C	0.43217600	1.24088000	-0.44779900
C	-0.95733400	0.72304700	-0.62989800
H	1.01522900	0.78496200	0.35557200
O	-1.61248400	1.25015200	-1.74646000
H	-0.84277700	-0.41054400	-1.01325500

C	-3.56219900	0.63292900	1.89980200
N	-2.56036900	0.24590400	2.63857800
N	-3.16156200	0.94182600	0.62406600
N	-1.49645700	0.28578300	1.78952100
C	-1.83056200	0.70495200	0.55593200
C	-0.22144200	-0.25022800	2.17202600
C	0.06138000	-1.56866000	1.80627100
C	0.71556600	0.58684500	2.78371400
C	1.36199200	-2.03102700	2.02657200
C	1.98526300	0.08078800	2.99726700
C	2.33584000	-1.21425300	2.60159900
C	-4.98797900	0.85192200	2.24282800
C	-4.15248200	1.24902800	-0.41991700
C	-5.40377000	1.98097900	0.19412400
O	-5.38830200	2.05365700	1.61158900
C	-6.63891600	1.21840000	-0.34781200
H	-7.16049800	1.82504100	-1.09478100
H	-7.34846700	1.03669000	0.46618400
C	-6.09423400	-0.04762400	-0.95987800
C	-6.78928800	-1.14892800	-1.44390000
C	-4.70327200	-0.02864400	-1.00974900
C	-6.06946800	-2.21711900	-1.97719300
H	-7.87442900	-1.17757100	-1.41026900
C	-3.97547600	-1.08995900	-1.53770800
C	-4.67294500	-2.18964200	-2.02897100
H	-6.60151800	-3.08153600	-2.36180800
H	-2.88978300	-1.08855700	-1.58292700
H	-4.10734900	-3.01643000	-2.44479600

H	-5.60606900	-0.00131500	1.90953500
H	-5.10501400	0.97564300	3.31918200
H	2.74480100	0.72123500	3.45651900
H	1.61792100	-3.03921200	1.71842200
C	0.36237600	2.02610000	3.08248300
H	-0.59364900	2.09736100	3.61749600
H	0.27578000	2.59207000	2.15257200
H	1.13538800	2.48027800	3.69668500
C	-0.98948900	-2.45585600	1.19143700
H	-1.16849400	-2.22023400	0.13635900
H	-1.94164100	-2.35990100	1.72161900
H	-0.68412200	-3.49966700	1.23929300
C	3.72522700	-1.73766700	2.82023300
H	4.47062700	-0.95733500	2.64660800
H	3.94233800	-2.56901400	2.13326500
H	3.85527800	-2.09831400	3.84605500
C	4.83538600	-1.12898500	-0.46987100
C	3.51212900	-1.19602800	-0.88311400
C	2.86925600	-2.43121600	-1.02727100
C	3.58612600	-3.58807800	-0.69541200
C	4.91551700	-3.51617300	-0.28779400
C	5.55858700	-2.27697000	-0.16238500
H	5.31178700	-0.15505100	-0.37537200
H	2.96621400	-0.27935700	-1.10198000
H	3.10910200	-4.55626500	-0.79237200
H	5.46491900	-4.43024700	-0.06161300
C	1.47532500	-2.52721100	-1.48313800
H	0.89963600	-3.39495300	-1.15463800

C	0.84706500	-1.61725100	-2.24906000
H	1.37874700	-0.73215600	-2.60891000
C	-0.65585500	-1.56045400	-2.26987800
H	-1.51736200	0.50096400	-2.39530600
O	-1.32051800	-2.57737200	-2.45545800
C	6.51984000	3.94969200	-1.12760400
H	6.76439400	4.40841900	-0.16525600
H	6.71472700	4.68219400	-1.91368700
H	7.20796600	3.11073600	-1.27300700
C	6.97822900	-2.20148700	0.36513200
H	7.37675200	-1.03986900	0.28377100
H	7.67447000	-2.95891400	-0.30018700
H	6.97278500	-2.57248300	1.55094100
H	-3.65116900	1.86640200	-1.16195600
H	-5.40677400	3.02370100	-0.12335500

Si-M02BA

Zero-point correction= 0.590026 (Hartree/Particle)

C	4.56438200	3.05558400	2.47775500
C	3.33533600	2.51164600	2.12405600
C	2.96482900	2.39176900	0.77891000
C	3.85613000	2.86428900	-0.19150200
C	5.08725100	3.40084100	0.16583100
C	5.46450900	3.50420500	1.50662100
H	4.83186900	3.13802700	3.52861000
H	2.64915200	2.17896700	2.89807900
H	3.58989400	2.77277000	-1.24181800
H	5.77199300	3.74019400	-0.60771200
C	1.73983400	1.70052600	0.34963900

H	1.33571700	1.93228800	-0.63573400
C	1.14108000	0.71137500	1.01421000
C	6.78645000	4.11401200	1.89594900
H	6.69643600	5.19789500	2.02649400
H	7.15227000	3.70026900	2.83917300
H	7.54435100	3.93843000	1.12812000
C	-0.02991400	-0.04981000	0.42739600
H	1.49061300	0.39861900	1.99987100
O	-0.45638300	0.36836700	-0.79308900
H	-0.83240600	-0.07965000	1.19059800
C	4.06876400	-0.61693400	0.54787400
C	5.95849900	-0.51328600	-1.53185600
C	5.39805200	-0.29027600	0.81401900
C	3.69329200	-0.86781300	-0.76362700
C	4.63641000	-0.84589300	-1.79414700
C	6.33098900	-0.22184000	-0.21904900
C	3.99571600	-1.23968100	-3.10691900
C	2.70784400	-1.92435400	-2.64819900
C	2.33147900	-1.14841300	-1.36784300
N	1.38553500	-1.95289300	-0.59452100
C	1.15284300	-3.28143400	-0.83514800
C	1.97344400	-4.03226400	-1.84290500
O	3.05800900	-3.25606800	-2.29077500
N	0.09359900	-3.69810100	-0.20714000
N	-0.36459400	-2.58139300	0.43955200
C	0.38362200	-1.50815900	0.19753500
C	-1.67148700	-2.53870500	1.04006300
C	-4.16265000	-2.12766600	2.13996300

C	-2.75066400	-2.33826400	0.17431000
C	-1.78950400	-2.56926700	2.42618100
C	-3.06135100	-2.36988500	2.95825200
C	-3.99941900	-2.15714500	0.75423400
C	-2.53630300	-2.25637600	-1.31286900
C	-0.57768500	-2.73717200	3.30290900
C	-5.48794800	-1.71476500	2.71322800
H	3.34357300	-0.66044700	1.35263200
H	6.69268800	-0.49273700	-2.33211300
H	5.70003700	-0.06808300	1.83237400
H	7.35953400	0.04533200	0.00266900
H	3.75461700	-0.36265100	-3.71846700
H	4.60588600	-1.91991400	-3.70579200
H	1.90095500	-1.92617000	-3.39331800
H	1.78914700	-0.22451600	-1.60490400
H	1.31274700	-4.31154800	-2.67657300
H	2.38122000	-4.94562200	-1.40546500
H	-3.18245900	-2.34898900	4.03838100
H	-4.84938200	-1.93281500	0.11592700
H	-3.47988800	-2.03953500	-1.81573800
H	-2.13205800	-3.19434100	-1.70774000
H	-1.83567500	-1.44232300	-1.53455100
H	-0.87437300	-2.93071400	4.33522800
H	0.05486700	-3.56235100	2.96290000
H	0.03291500	-1.82688000	3.29729400
H	-5.54117100	-1.89531500	3.78925500
H	-6.31663900	-2.23836200	2.22741200
H	-5.59830300	-0.64221900	2.51368700

C	-3.52429300	0.75472900	0.77340200
O	-4.77703500	0.89412500	0.81983800
O	-2.71656700	0.67299500	1.71485900
H	-2.01585300	0.61255000	-0.54435500
O	-3.02423500	0.71680900	-0.52351600
Cs	-5.01239200	2.68889600	-1.48814200

Re-M02BA

Zero-point correction= 0.589299 (Hartree/Particle)

C	5.08481100	-1.49937100	-2.15684300
C	3.81257200	-1.19716900	-1.68796500
C	3.62023300	-0.70449400	-0.38739600
C	4.74983400	-0.54505200	0.42603600
C	6.02757900	-0.84538100	-0.04158400
C	6.20200500	-1.31915000	-1.33891200
H	5.20884600	-1.87832600	-3.16673600
H	2.95482200	-1.35214700	-2.33572200
H	4.61657300	-0.17901100	1.44091300
H	6.88590300	-0.70993000	0.60934100
C	2.29584500	-0.36304200	0.15666500
H	2.25772600	-0.00409800	1.18500700
C	1.11866400	-0.47226200	-0.46082600
C	-0.17291300	-0.07716900	0.23788200
H	1.03719500	-0.82266600	-1.49110500
O	-0.06390000	0.37680700	1.50805400
H	-0.68716200	0.64764200	-0.43428600
C	-0.88859200	3.33393500	0.22700300
O	-0.69851700	4.50010700	-0.19307400
O	-1.86641100	2.58234100	-0.00639000

H	-0.04876200	1.85715600	1.28318900
O	0.12472200	2.83627000	1.01655600
Cs	2.18847900	4.76189700	0.17996800
C	-2.97058800	-2.36365900	0.77914100
N	-2.04670900	-3.27865900	0.73003500
N	-2.45586800	-1.12243600	0.54384500
N	-0.91107900	-2.56961400	0.43571100
C	-1.13894500	-1.25752800	0.32631800
C	0.37522500	-3.20382700	0.36521500
C	0.80090600	-3.70217100	-0.86498700
C	1.18042300	-3.16722900	1.50470200
C	2.11732400	-4.15231000	-0.95005100
C	2.48582100	-3.63152900	1.36947000
C	2.97613100	-4.09904400	0.14828200
C	-4.40996200	-2.41820500	1.17273400
C	-3.29040200	0.07705900	0.53508100
C	-4.42639100	-0.05387700	1.60894700
O	-4.60042700	-1.37763300	2.11086700
H	-4.17654300	0.53405200	2.49185700
H	-2.64041900	0.95096000	0.70487400
C	-5.70240800	0.46653100	0.90832500
H	-5.90343700	1.49823000	1.21506900
H	-6.56515200	-0.13842700	1.20319500
C	-5.39165800	0.40826600	-0.56621500
C	-6.25103200	0.55008200	-1.65206700
C	-4.03088900	0.20434100	-0.77581700
C	-5.72507500	0.48240600	-2.94067800
H	-7.31451600	0.70809800	-1.49877000

C	-3.49715400	0.14570000	-2.05736700
C	-4.35728800	0.28342000	-3.14374300
H	-6.38410800	0.58801100	-3.79651300
H	-2.43013300	-0.00032100	-2.20600800
H	-3.96373800	0.24182200	-4.15403700
H	-5.05852500	-2.30364600	0.29304600
H	-4.63564200	-3.36343600	1.66611700
H	3.14979100	-3.59095100	2.22975600
H	2.48817400	-4.52460700	-1.90188600
C	0.67221500	-2.55716600	2.78118600
H	-0.26969000	-3.02138600	3.09034500
H	0.48551300	-1.48442900	2.62794300
H	1.40082900	-2.68615300	3.58354500
C	-0.12113500	-3.70393700	-2.05486400
H	-0.40977800	-2.68336200	-2.33225600
H	-1.04308200	-4.25058700	-1.83721500
H	0.36170500	-4.16598500	-2.91734400
C	4.42786600	-4.46950900	0.01116000
H	4.60887600	-5.05247400	-0.89451400
H	4.77443800	-5.04683700	0.87226300
H	5.03296100	-3.55736500	-0.04780200
C	7.61081844	-1.65289372	-1.86375466
H	8.09213335	-0.75452228	-2.18958391
H	8.18589030	-2.10194298	-1.08110076
H	7.53328214	-2.33410272	-2.68524260

Si-TS2BA -1282.66

Zero-point correction= 0.584946 (Hartree/Particle)

C	4.80771753	3.20156701	2.25842846

C	3.62303487	2.57905990	1.88377174
C	3.27669447	2.43473818	0.53190734
C	4.15524298	2.97165607	-0.41922819
C	5.33935761	3.59314455	-0.04075544
C	5.69057191	3.71792516	1.30512908
H	5.05114466	3.29769269	3.31428295
H	2.94904631	2.20568757	2.64968167
H	3.91408863	2.86750374	-1.47424908
H	6.00906336	3.98393548	-0.80340294
C	2.09877330	1.68086680	0.08554909
H	1.74381552	1.84102597	-0.93150893
C	1.49920714	0.70929215	0.78741554
C	6.96368260	4.42538929	1.71312448
H	6.79580668	5.49148439	1.86819055
H	7.32630122	4.00287339	2.66002816
H	7.74211568	4.30987439	0.96212155
C	0.37229930	-0.09976488	0.26775375
H	1.83721289	0.47691749	1.80102408
O	0.01480038	0.11617341	-1.05076577
H	-1.05318641	0.27132883	1.10030641
C	4.42270379	-0.64053477	0.34321162
C	6.21689817	-0.99566710	-1.79257209
C	5.76523569	-0.28684438	0.47854579
C	3.98596145	-1.15287343	-0.86993062
C	4.88116172	-1.34875971	-1.92541008
C	6.65244443	-0.45219333	-0.58327599
C	4.17615664	-1.96945338	-3.11094815
C	2.90507106	-2.53111857	-2.47377720

C	2.59510507	-1.52625342	-1.34507607
N	1.68361211	-2.16826560	-0.39721857
C	1.44086313	-3.51311257	-0.36565476
C	2.20947196	-4.44880808	-1.25243510
O	3.26735677	-3.78048537	-1.89385325
N	0.40940943	-3.79202411	0.37667988
N	-0.02045776	-2.56504085	0.80845631
C	0.71045781	-1.55933660	0.32659657
C	-1.30062871	-2.39754107	1.43741112
C	-3.74430886	-1.75091450	2.53390659
C	-2.40784552	-2.32533113	0.58702325
C	-1.36981683	-2.19148995	2.81010833
C	-2.61813033	-1.87672317	3.34350318
C	-3.63084914	-2.01633723	1.16755854
C	-2.24106043	-2.50161341	-0.89857141
C	-0.12671011	-2.21996141	3.65720386
C	-5.04171757	-1.22956019	3.08520981
H	3.73463768	-0.50044943	1.16839296
H	6.91368007	-1.14795430	-2.61180983
H	6.11302584	0.13748127	1.41508855
H	7.69281598	-0.16602538	-0.46487265
H	3.91649025	-1.21609772	-3.86356181
H	4.74917697	-2.76148476	-3.59885941
H	2.06356230	-2.66022418	-3.16771750
H	2.03877324	-0.65234507	-1.71251909
H	1.50616893	-4.87554125	-1.98261623
H	2.64188835	-5.26653698	-0.67237470
H	-2.69941643	-1.67390482	4.40847489

H	-4.50594380	-1.91214120	0.53060559
H	-3.20300679	-2.39164186	-1.40312300
H	-1.83341692	-3.48967349	-1.13570876
H	-1.55600285	-1.73669633	-1.28548798
H	-0.38214411	-2.24181278	4.71806198
H	0.49614243	-3.08930377	3.42865549
H	0.47441415	-1.32202459	3.47403722
H	-5.09013379	-1.32523649	4.17231469
H	-5.90166380	-1.74610536	2.64950114
H	-5.10795702	-0.16906017	2.81860073
C	-3.00748038	0.90873870	0.69847514
O	-4.18351933	1.22422889	0.97178256
O	-2.05911441	0.74298920	1.52660087
H	-1.42279537	0.48001592	-0.85684005
O	-3.01416535	0.79273278	-0.57222034
Cs	-5.07875328	2.17185717	-1.69066526

Re-TS2BA -1236.31

Zero-point correction= 0.584512 (Hartree/Particle)

C	-1.24406011	-3.27053607	2.35542181
C	-0.50395217	-4.22047827	4.90257326
C	-2.20934720	-3.74616213	3.24427618
C	0.08063438	-3.27316519	2.76045930
C	0.45701603	-3.75681206	4.01422226
C	-1.84402704	-4.20740946	4.50870187
C	1.96143610	-3.70289961	4.16405209
C	2.43553598	-3.59779656	2.71119419
C	1.31132147	-2.79049845	2.02290385
N	1.32899950	-3.02250131	0.57929727

C	2.02064399	-4.06371995	0.00886976
C	2.87409951	-4.96268857	0.85256044
O	2.46682067	-4.92085457	2.19756877
N	1.92646878	-4.07857838	-1.27663437
N	1.13660940	-2.98413280	-1.57231180
C	0.74223040	-2.30107965	-0.44883052
C	0.80409165	-2.70108419	-2.92954471
C	0.08100161	-2.03127088	-5.52278136
C	1.58318112	-1.77167971	-3.62682520
C	-0.30884879	-3.32779794	-3.49573890
C	-0.65146541	-2.97450258	-4.80175291
C	1.20138847	-1.44654300	-4.92568205
C	2.74881025	-1.10563014	-2.94887145
C	-1.11649744	-4.32280496	-2.70721139
C	-0.34343673	-1.62502481	-6.91057027
H	-1.53265942	-2.92907165	1.36921971
H	-0.21877458	-4.60583604	5.87741881
H	-3.25004510	-3.75876267	2.93486866
H	-2.60570583	-4.57835143	5.18777124
H	2.27798262	-2.81762485	4.72783279
H	2.38927081	-4.58652988	4.64390169
H	3.42211069	-3.12721691	2.59459274
H	1.45439437	-1.72105238	2.18931489
H	3.92445450	-4.64734398	0.75485190
H	2.78799338	-5.99315809	0.50466624
H	-1.51755295	-3.44417746	-5.26182327
H	1.78461120	-0.71610427	-5.48152988
H	3.31885174	-0.50300359	-3.65807858

H	3.41614434	-1.84564745	-2.49809807
H	2.39435252	-0.44764757	-2.14634232
H	-1.79329877	-4.87054148	-3.36583710
H	-0.46339718	-5.03943093	-2.20204995
H	-1.72317552	-3.82147793	-1.94457232
H	-1.01261305	-0.75879243	-6.86915563
H	-0.88032817	-2.43251185	-7.41333928
H	0.51783320	-1.34751005	-7.52276920
C	0.35424378	-0.64127852	-0.60752588
H	0.24639115	0.00051456	0.60262096
C	-1.06821507	-0.90028996	-0.81154191
H	-1.48220307	-1.70373596	-0.21279591
C	-1.90779307	-0.05319696	-1.44317391
H	-1.47920907	0.74951904	-2.03769191
C	-3.36802607	-0.13782196	-1.41449891
C	-4.12232007	0.71753904	-2.23079891
C	-4.08194907	-1.04615196	-0.60933091
C	-5.51307307	0.66859904	-2.25277491
H	-3.59998307	1.42625704	-2.86944991
C	-5.46721407	-1.09168596	-0.63730891
H	-3.54649007	-1.72353096	0.05152009
C	-6.21417707	-0.23750796	-1.45882591
H	-6.06404707	1.34435104	-2.90302691
H	-5.98959507	-1.80577396	-0.00383891
C	-7.71948607	-0.30644696	-1.47531391
H	-8.14115107	0.43445604	-2.15870991
H	-8.06770507	-1.29386396	-1.79509691
H	-8.13712207	-0.12360096	-0.48006591

O	0.90645293	0.28155804	-1.57842291
C	0.03133000	2.06690400	1.40148200
O	0.15249200	2.32162500	0.15536500
O	-0.18340500	2.91048700	2.29110400
H	0.65878207	1.32428096	-0.90198809
O	0.14736300	0.77322900	1.79638500
Cs	-1.12551300	4.98787900	0.40893600

Si-M03BA

Zero-point correction= 0.589971 (Hartree/Particle)

C	5.64435700	0.81199600	2.48373300
C	4.42022400	0.63313700	1.86740700
C	3.28837800	0.21585200	2.58877500
C	3.46078700	0.00052600	3.95846300
C	4.68554900	0.18884800	4.57188700
C	5.80367100	0.59980500	3.84836800
H	6.49451500	1.15156800	1.89552600
H	4.32414000	0.86732700	0.81350700
H	2.60616700	-0.31265400	4.55161900
H	4.77692700	0.02338500	5.64154300
C	1.96500800	0.08176700	1.99212100
H	1.11158500	0.12337200	2.66357800
C	1.73109900	-0.00893900	0.66528600
C	7.13590800	0.80796800	4.51043500
H	7.74432700	-0.10223300	4.47992100
H	7.70238600	1.59878600	4.01147100
H	7.01561500	1.08767800	5.55932300
C	0.46110500	0.15208500	0.03149300
H	2.57199100	-0.12073400	-0.01420000

O	-0.60846400	0.55246000	0.79331600
C	0.14596300	3.47299500	0.33440000
C	-1.62751800	5.62026000	-0.09110000
C	0.32551500	4.68875500	0.98984900
C	-0.93048100	3.34750500	-0.53685300
C	-1.80245300	4.41368800	-0.75347300
C	-0.55463600	5.75028300	0.78564200
C	-2.87646300	4.03510500	-1.74629100
C	-2.28919000	2.79922600	-2.42854600
C	-1.41289800	2.15157700	-1.33016600
N	-0.40137300	1.31639500	-1.94674000
C	-0.06296800	1.36198500	-3.26888400
C	-0.86059900	2.21990000	-4.20146000
O	-1.47650300	3.26828300	-3.49209000
N	0.90383800	0.57109800	-3.56601800
N	1.25607100	-0.02905700	-2.34411800
C	0.45893900	0.43938000	-1.32119800
C	1.69116300	-1.39800800	-2.44708700
C	2.44906800	-4.04108000	-2.88051200
C	0.69988200	-2.37314700	-2.63405100
C	3.04965600	-1.71354200	-2.46914800
C	3.40400500	-3.04787300	-2.68954300
C	1.10304400	-3.68432800	-2.83820800
C	-0.75628300	-1.99542100	-2.69769200
C	4.11467000	-0.66961800	-2.27758500
C	2.83721900	-5.46575900	-3.17503600
H	0.82468400	2.64455400	0.51484800
H	-2.31010800	6.44795200	-0.25857500

H	1.15708800	4.80633700	1.67568200
H	-0.40020500	6.68487600	1.31420700
H	-3.81607700	3.78051900	-1.24452300
H	-3.08363000	4.81478100	-2.48339500
H	-3.04132000	2.09995400	-2.81796100
H	-2.02401300	1.50370400	-0.69382400
H	-1.61430900	1.59358300	-4.70212100
H	-0.21352300	2.66112700	-4.95963900
H	4.45799800	-3.30934300	-2.73288900
H	0.34417300	-4.44883400	-2.98373700
H	-1.37058200	-2.88578000	-2.83496000
H	-0.92556800	-1.31940600	-3.54208200
H	-1.09506400	-1.49945100	-1.78271500
H	5.02316000	-0.95117300	-2.81423700
H	3.78718100	0.31091900	-2.62579500
H	4.37761900	-0.58524400	-1.21764700
H	3.91612500	-5.60865500	-3.10171200
H	2.52816900	-5.74700800	-4.18674900
H	2.34730900	-6.15448200	-2.48343100
C	-2.07048200	-2.50344200	0.67982500
O	-2.81400100	-3.42547500	1.00356800
O	-0.79933700	-2.81775700	0.30805100
O	-2.36594600	-1.27227100	0.68008300
Cs	-4.74831800	-1.66911700	2.48856100
H	-1.30595100	-0.17171500	0.74837800
H	-0.33651300	-1.98214700	0.26931500

Re-M03BA

Zero-point correction= 0.590514 (Hartree/Particle)

C	-5.02915100	-1.05119000	1.86906100
C	-3.72479400	-0.82369900	1.45583000
C	-3.44594800	-0.36135700	0.15927400
C	-4.53203000	-0.14798600	-0.69705700
C	-5.84208400	-0.37491400	-0.27888200
C	-6.11474200	-0.83063400	1.01063600
H	-5.21632300	-1.41206900	2.87770900
H	-2.90773300	-1.01919200	2.14427300
H	-4.34230400	0.19893000	-1.70986300
H	-6.66431200	-0.19944700	-0.96807900
C	-2.08174100	-0.11670600	-0.33521100
H	-1.97713300	0.20406900	-1.37180100
C	-0.93938800	-0.28158700	0.33371100
C	0.40121100	-0.00170300	-0.32685100
H	-0.92148800	-0.60139900	1.37696600
O	0.37233000	0.39572000	-1.61986700
C	1.26507500	3.34170800	-0.37581500
O	1.05891500	4.49078100	0.08178500
O	2.23149100	2.57838600	-0.13406300
O	0.28014800	2.87696000	-1.22021200
Cs	-1.85831100	4.60241500	-0.17142100
C	3.06660800	-2.47347900	-0.66491500
N	2.08552700	-3.32767700	-0.62916500
N	2.62168600	-1.19617600	-0.48702600
N	0.98535100	-2.54074000	-0.40501100
C	1.29106700	-1.24256800	-0.32508200
C	-0.34041600	-3.09139000	-0.36741700
C	-0.84523700	-3.51713800	0.85996500

C	-1.09571100	-3.04874500	-1.54071000
C	-2.19029800	-3.88115300	0.90545000
C	-2.43250900	-3.42477600	-1.44378300
C	-3.00002400	-3.81407300	-0.22848100
C	4.51422400	-2.62614900	-0.99851600
C	3.52916500	-0.05151900	-0.48121500
C	4.68323500	-0.28124200	-1.51762400
O	4.80257600	-1.63180500	-1.96183800
H	4.48711200	0.28284200	-2.42926200
H	2.94228700	0.85728500	-0.69508000
C	5.96406100	0.20126900	-0.80026000
H	6.21460700	1.21645600	-1.12535200
H	6.80671800	-0.44621200	-1.06034600
C	5.61634300	0.19124400	0.66723800
C	6.45592900	0.31002700	1.77089300
C	4.24193700	0.06228700	0.84682700
C	5.89704300	0.29487400	3.04748600
H	7.52953000	0.40934400	1.64074500
C	3.67624700	0.05540900	2.11595400
C	4.51663000	0.17009400	3.22060800
H	6.54042700	0.38279000	3.91709400
H	2.60005200	-0.03521800	2.24134400
H	4.09782800	0.16719900	4.22157300
H	5.13442500	-2.51981800	-0.09781700
H	4.70116500	-3.59925900	-1.45234400
H	-3.05803100	-3.37458300	-2.33196000
H	-2.62122700	-4.19388900	1.85340100
C	-0.49861100	-2.52476300	-2.81694500

H	0.42237000	-3.06057100	-3.06791800
H	-0.24823500	-1.46082800	-2.69753000
H	-1.20179100	-2.64032000	-3.64366500
C	0.02625000	-3.53158000	2.08732300
H	0.35040100	-2.51862800	2.35354000
H	0.92982100	-4.12490700	1.92132600
H	-0.51274400	-3.94946000	2.93900200
C	-4.47712300	-4.08863600	-0.14202500
H	-4.74121200	-4.56509000	0.80466700
H	-4.81091000	-4.73192700	-0.96064700
H	-5.02832600	-3.14349500	-0.21082700
C	-7.52275200	-1.12043600	1.46162200
H	-7.69891200	-0.75154600	2.47572200
H	-8.25546100	-0.65767700	0.79675000
H	-7.71493500	-2.19878900	1.46942600
H	2.07069500	1.67611816	0.15169459
H	0.30031921	1.31031564	-1.33714968

Si-TS2BA1 -1283.50

Zero-point correction= 0.583962 (Hartree/Particle)

C	-4.10929900	-3.44357900	1.11305400
C	-2.88349800	-2.92232700	0.71199600
C	-2.72919300	-2.30740000	-0.54009600
C	-3.85671900	-2.27089000	-1.37746300
C	-5.07676700	-2.79602100	-0.97482400
C	-5.23070700	-3.38839900	0.28264900
H	-4.19411200	-3.91785100	2.08830600
H	-2.02640600	-3.01233500	1.37317400
H	-3.76604800	-1.80462400	-2.35499500

H	-5.93062300	-2.74541200	-1.64718700
C	-1.48901900	-1.65847700	-0.97013700
H	-1.38404600	-1.41140300	-2.02420400
C	-0.51747600	-1.23858700	-0.14066000
C	-6.56086900	-3.95204800	0.71411100
H	-6.48728400	-4.43087700	1.69356300
H	-7.32282900	-3.16828200	0.78060700
H	-6.92636800	-4.69851300	0.00188000
C	0.68838200	-0.51572400	-0.56234900
H	-0.59802300	-1.43080600	0.93241800
O	0.71555300	-0.10846600	-1.89105500
H	1.88749600	-1.46256500	-0.43340300
C	-2.91607100	0.70870000	0.84675700
C	-4.75641500	2.55381300	-0.20823100
C	-4.27244600	0.61701200	1.16114600
C	-2.49485700	1.71488500	-0.01053800
C	-3.40778700	2.64135400	-0.52392900
C	-5.18613600	1.52733500	0.63398200
C	-2.70332300	3.65333400	-1.40006700
C	-1.24716600	3.49934900	-0.96375600
C	-1.13124900	1.99942700	-0.61637600
N	0.05844600	1.81593700	0.21669400
C	0.70680200	2.83243500	0.85738800
C	0.18022500	4.23556600	0.79242900
O	-1.11080500	4.26183400	0.23326200
N	1.84818600	2.43475000	1.34058700
N	1.92713500	1.12272400	0.96158400
C	0.86926800	0.72578600	0.25473800

C	3.16942900	0.39914600	1.03511500
C	5.39763900	-1.20418100	0.87240800
C	4.08335400	0.64314700	0.00501800
C	3.34409600	-0.57581100	2.00927900
C	4.49226200	-1.36067100	1.91880600
C	5.20734600	-0.16833300	-0.04426300
C	3.79721900	1.67758000	-1.04962500
C	2.28121700	-0.83879400	3.04187300
C	6.48549900	-2.20619300	0.61431600
H	-2.21434000	-0.00775500	1.25480500
H	-5.46420900	3.27533600	-0.60620400
H	-4.61372100	-0.18475300	1.80851500
H	-6.23889300	1.44047700	0.88396600
H	-2.80480400	3.40224700	-2.46193900
H	-3.04753400	4.67978800	-1.25266200
H	-0.50726000	3.81873500	-1.70899500
H	-0.95303800	1.38196900	-1.50724200
H	0.88434800	4.83322100	0.19521000
H	0.11504100	4.67337200	1.79024400
H	4.63541200	-2.16607800	2.63535400
H	5.87907100	-0.08299300	-0.89267400
H	4.59055400	1.67418100	-1.79774900
H	3.71295300	2.67980800	-0.61581400
H	2.86669600	1.42154000	-1.56717100
H	2.67540200	-1.45345800	3.85350200
H	1.88754000	0.08901500	3.46709800
H	1.44345900	-1.38162700	2.58869900
H	6.59416300	-2.91494600	1.43886800

H	7.45022400	-1.72195500	0.43469100
H	6.19286800	-2.73746500	-0.30002100
C	3.59904700	-1.82622100	-1.84397400
O	4.74153500	-2.21677100	-2.17297600
O	2.98689800	-2.34898100	-0.80585100
H	1.75713100	-0.50761400	-2.23917600
O	3.00107700	-0.87180600	-2.52290200

Re-TS2BA1 -1022.55

Zero-point correction= 0.583718 (Hartree/Particle)

C	-3.40766600	-1.34942200	0.91080200
C	-6.05815600	-1.78127100	0.05170800
C	-4.29908000	-2.08175100	1.69629000
C	-3.85876500	-0.85301000	-0.30038700
C	-5.17170000	-1.05217200	-0.72898900
C	-5.60827900	-2.30029600	1.26848000
C	-5.39293400	-0.37451200	-2.06405900
C	-4.23583300	0.62755700	-2.12055100
C	-3.09756100	-0.09330400	-1.36480500
N	-2.11269500	0.88054700	-0.88445000
C	-2.38486500	2.22224000	-0.80692200
C	-3.67989100	2.77147600	-1.32549900
O	-4.66337300	1.77039100	-1.38800600
N	-1.37811000	2.90901200	-0.38022100
N	-0.39149800	1.96434100	-0.17653100
C	-0.80527800	0.69711600	-0.48165000
C	0.85738300	2.38117200	0.37572800
C	3.30933700	3.12366400	1.43816300
C	1.89324000	2.72054900	-0.49866400

C	0.99887900	2.41595300	1.76687800
C	2.24237100	2.79192000	2.27431000
C	3.11791200	3.08954300	0.05553000
C	1.69823600	2.60741200	-1.98588700
C	-0.14126000	2.03216100	2.67007100
C	4.63867400	3.52332100	2.02580900
H	-2.39560300	-1.16729000	1.25265300
H	-7.08401600	-1.93755800	-0.27068300
H	-3.95161500	-2.48786900	2.64090100
H	-6.28847100	-2.87405100	1.89076700
H	-5.31325200	-1.08688600	-2.89355700
H	-6.35205600	0.14314000	-2.14716500
H	-3.94617700	0.92535400	-3.13842200
H	-2.56763900	-0.79348900	-2.01154200
H	-3.50149100	3.20489000	-2.32210200
H	-4.04208800	3.56111200	-0.66540000
H	2.38229700	2.81487500	3.35251300
H	3.94249700	3.34300600	-0.60676500
H	2.54862600	3.03208500	-2.52249600
H	0.78659200	3.12079500	-2.30384000
H	1.60202000	1.55306700	-2.27293500
H	-1.07576900	2.48786700	2.33070800
H	-0.28142100	0.94405600	2.68790000
H	0.05705400	2.36433800	3.69111500
H	4.57578200	4.50852400	2.49883700
H	5.41403500	3.56746200	1.25791300
H	4.95557500	2.81138600	2.79243400
C	-0.13526700	-0.55162100	-0.36521100

H	-0.25390300	-0.77087100	1.05832600
C	1.33278200	-0.59245900	-0.43569200
H	1.88399600	0.24766600	-0.02565300
C	2.00064400	-1.66770800	-0.88923800
H	1.41409700	-2.51549100	-1.23276300
C	3.45929000	-1.79388200	-0.94527100
C	4.03354400	-3.02021600	-1.30923500
C	4.34016100	-0.73137700	-0.67274400
C	5.41270900	-3.18491900	-1.38451100
H	3.37824000	-3.85834400	-1.53108400
C	5.71488100	-0.90150500	-0.74705100
H	3.94337200	0.24554100	-0.40698300
C	6.28172200	-2.13140100	-1.10031500
H	5.82211200	-4.15212900	-1.66726100
H	6.36869200	-0.05912300	-0.52997800
C	7.77771000	-2.31154800	-1.14543600
H	8.05387700	-3.17364300	-1.75794200
H	8.27258100	-1.42805900	-1.55913700
H	8.18786500	-2.47485400	-0.14266800
O	-0.81952300	-1.62992400	-0.86852200
C	-0.65690100	-2.51619900	2.33666600
O	-0.75947500	-3.17364500	1.21361300
O	-0.77327900	-3.05144100	3.44121000
H	-0.81787100	-2.44151000	0.12103000
O	-0.43410600	-1.19342600	2.26817800

Si-M2

Zero-point correction= 0.559851 (Hartree/Particle)

C	4.49160400	3.22106000	2.28351900

C	3.29245200	2.63511200	1.89492400
C	2.93630200	2.54563100	0.54100300
C	3.81943600	3.09948500	-0.39643200
C	5.01665400	3.68504800	-0.00382500
C	5.37826000	3.75454300	1.34371500
H	4.74205300	3.27607600	3.34059000
H	2.61417200	2.25219700	2.65228300
H	3.56967900	3.03893700	-1.45283400
H	5.68864100	4.09125300	-0.75629700
C	1.74253700	1.83094500	0.07389900
H	1.38248700	2.05009500	-0.92938100
C	1.14076600	0.83819900	0.74690500
C	6.66181600	4.41880900	1.77149600
H	7.03004900	4.00274700	2.71290700
H	7.44183300	4.29566300	1.01549000
H	6.51894800	5.49445400	1.92249800
C	-0.01297500	0.05376100	0.26333300
H	1.50309300	0.56503100	1.74093100
O	-0.36715800	0.32536700	-1.07719400
C	4.05017900	-0.53178000	0.33458700
C	5.86540100	-0.94271000	-1.77277300
C	5.39398900	-0.18653900	0.47887100
C	3.62258700	-1.06103200	-0.87419800
C	4.52808100	-1.28521100	-1.91544300
C	6.29163100	-0.37962400	-0.56916200
C	3.83290500	-1.92161400	-3.09843100
C	2.54935300	-2.45936900	-2.46653300
C	2.23278700	-1.42392400	-1.36580600

N	1.29110300	-2.03204100	-0.42303800
C	1.06513400	-3.37645300	-0.33702700
C	1.83375100	-4.33912200	-1.19405900
O	2.89747000	-3.69349200	-1.84865600
N	0.03924000	-3.63444400	0.42286500
N	-0.40371500	-2.40090700	0.80508100
C	0.31700600	-1.40775900	0.28702100
C	-1.67081100	-2.22046300	1.46099800
C	-4.07485000	-1.50629900	2.59166000
C	-2.79486300	-2.15727300	0.63290500
C	-1.70562200	-1.98867800	2.83046400
C	-2.93731800	-1.64475200	3.38290800
C	-3.99800400	-1.81192400	1.23154400
C	-2.66592400	-2.37466600	-0.85021200
C	-0.44440000	-2.01352500	3.65064100
C	-5.33852600	-0.91063700	3.14229300
H	3.35734600	-0.37353000	1.15181400
H	6.57011700	-1.11731200	-2.58060400
H	5.73282700	0.25339800	1.41149900
H	7.33300500	-0.10025100	-0.44416000
H	3.59071200	-1.18141000	-3.86960800
H	4.40466400	-2.72884500	-3.56210900
H	1.71599700	-2.60071800	-3.16771100
H	1.71389200	-0.54622600	-1.77081400
H	1.13149300	-4.78228100	-1.91521000
H	2.25892800	-5.14210700	-0.58887000
H	-2.99144300	-1.41396100	4.44389200
H	-4.88199200	-1.69545300	0.61001500

H	-3.64655600	-2.32161500	-1.32673700
H	-2.22033700	-3.34961300	-1.07292100
H	-2.03900100	-1.58662200	-1.28132900
H	-0.67734300	-2.01735200	4.71680700
H	0.16872100	-2.89049200	3.42414800
H	0.15759200	-1.12163000	3.44236100
H	-5.35219700	-0.91711700	4.23448600
H	-6.22714500	-1.43272500	2.77568900
H	-5.37247700	0.12469100	2.78364600
H	-1.39636400	0.55724300	-0.98738800

Re-M2

Zero-point correction= 0.559542 (Hartree/Particle)

C	2.71713500	1.98784800	0.98807500
C	5.44824200	2.66467400	1.07918000
C	3.18278600	2.94589800	1.89052800
C	3.63115100	1.37896600	0.13711600
C	4.98809400	1.70437900	0.18813600
C	4.53393300	3.28699500	1.92980900
C	5.76665500	0.87340300	-0.80688600
C	4.81297400	-0.28833400	-1.09925300
C	3.40910200	0.34626700	-0.95119200
N	2.41299900	-0.67697500	-0.66459700
C	2.74463000	-1.92108100	-0.17882700
C	4.17098400	-2.37028100	-0.22536000
O	5.02966000	-1.25958200	-0.08758400
N	1.72231900	-2.59572000	0.21701900
N	0.64439600	-1.75374000	-0.01303800
C	1.03494300	-0.53330200	-0.53224300

C	-0.68267600	-2.22638200	0.18514500
C	-3.32753400	-2.98799200	0.58163500
C	-1.47516600	-2.52315700	-0.93131000
C	-1.16839700	-2.32559700	1.49190400
C	-2.49696000	-2.71353800	1.66835600
C	-2.79851200	-2.89418700	-0.70824200
C	-0.91933300	-2.39760000	-2.32276500
C	-0.27971100	-1.97937000	2.65572700
C	-4.77235700	-3.36759500	0.78122500
H	1.66874100	1.70135300	0.98041100
H	6.50379800	2.91658900	1.12512000
H	2.48636400	3.42421500	2.57164700
H	4.88044900	4.03279900	2.63808700
H	5.96785200	1.43739300	-1.72488600
H	6.71735100	0.49919200	-0.41979400
H	4.95490700	-0.74392100	-2.08898100
H	3.12945900	0.84126400	-1.88469200
H	4.36074500	-2.88656900	-1.17900700
H	4.37166200	-3.06065900	0.59437600
H	-2.89565500	-2.78618100	2.67732700
H	-3.43472600	-3.11425800	-1.56264900
H	-1.56568900	-2.90192300	-3.04333200
H	0.08280300	-2.83176600	-2.38387300
H	-0.83921300	-1.34268100	-2.60808800
H	0.57286500	-2.66216300	2.71281700
H	0.12797800	-0.96924500	2.54056200
H	-0.83285900	-2.02696300	3.59527800
H	-4.98565600	-4.34482200	0.33807000

H	-5.43334800	-2.64001900	0.29990500
H	-5.02898300	-3.41114000	1.84163300
C	0.27308500	0.55950100	-0.87617400
C	-1.10742100	0.74017700	-0.54219200
H	-1.53615300	0.01432700	0.14071200
C	-1.88233100	1.76489300	-0.96590100
H	-1.45898000	2.50656000	-1.64027800
C	-3.28671800	1.95173900	-0.59730100
C	-3.93868500	3.14908700	-0.92279500
C	-4.04382400	0.96915000	0.06831500
C	-5.27230300	3.36474300	-0.59000900
H	-3.38406200	3.92621500	-1.44297700
C	-5.37103300	1.19192400	0.40079500
H	-3.59405200	0.01014800	0.31485700
C	-6.01504100	2.39335200	0.07988700
H	-5.74466700	4.30761400	-0.85516800
H	-5.92815100	0.41275200	0.91699800
C	-7.46450900	2.60929600	0.43088000
H	-7.76435700	3.64422900	0.24998100
H	-8.11670300	1.96388600	-0.16690300
H	-7.65699500	2.37949000	1.48309500
O	0.91679200	1.55835400	-1.60577500
H	0.87845700	2.37588200	-1.08666000

TS3D -1608.73

Zero-point correction= 0.555303 (Hartree/Particle)

C	-3.14938100	-1.31269800	-1.66649000
C	-5.73504800	-2.23292600	-1.02485100
C	-4.01847700	-1.74989000	-2.66627200

C	-3.59753100	-1.32968100	-0.35299700
C	-4.87638600	-1.78554600	-0.02965600
C	-5.29710000	-2.20747800	-2.34890900
C	-5.10709600	-1.69920200	1.46189600
C	-4.05431300	-0.67890800	1.89834800
C	-2.87970500	-0.93408900	0.92304700
N	-2.04934100	0.27389900	0.82497500
C	-2.46785200	1.51177200	1.23937400
C	-3.76219200	1.66031800	1.97703700
O	-4.62564900	0.60002800	1.65339200
N	-1.59098500	2.43155100	0.98745300
N	-0.55555600	1.75428400	0.39196500
C	-0.79733800	0.42798800	0.30334000
C	0.58973100	2.47135800	-0.07939400
C	2.81297900	3.84667300	-0.98712700
C	1.54730200	2.87507200	0.85118200
C	0.69720000	2.73133100	-1.44864800
C	1.82573600	3.42228700	-1.88009400
C	2.65720300	3.56870000	0.37103200
C	1.38726000	2.53238800	2.30771900
C	-0.35217200	2.24433400	-2.41001300
C	4.02610300	4.58212700	-1.49406600
H	-2.14024600	-0.99222000	-1.90191400
H	-6.73304900	-2.58372900	-0.77919300
H	-3.69267400	-1.74034200	-3.70135100
H	-5.95882500	-2.54487200	-3.14042200
H	-4.92455400	-2.66417600	1.94815800
H	-6.10742300	-1.35836400	1.73852200

H	-3.55425200	1.68452300	3.05754300
H	-4.24947300	2.59443100	1.69440100
H	1.93851900	3.63526900	-2.94048800
H	3.42040100	3.89205700	1.07419900
H	2.24510300	2.88202500	2.88389300
H	0.48017900	2.98309100	2.72078400
H	1.30385100	1.44750400	2.43868400
H	-1.35945700	2.47833500	-2.05286700
H	-0.28263200	1.15626900	-2.52334600
H	-0.21707300	2.69961000	-3.39219100
H	3.73524600	5.45647000	-2.08297200
H	4.65853700	4.91897500	-0.67049900
H	4.62734000	3.93652100	-2.14099100
C	0.03451200	-0.61166100	-0.20255300
C	1.40395600	-0.54092200	-0.37191600
H	2.00614700	0.32590100	-0.11767900
C	1.93762100	-1.85523500	-0.66464000
H	1.67874000	-2.23381100	-1.66032100
C	3.30340100	-2.19714100	-0.23988800
C	4.08420100	-3.11695900	-0.95807600
C	3.87717300	-1.64581700	0.91728000
C	5.36508100	-3.46098900	-0.54573000
H	3.67389000	-3.56063300	-1.86207500
C	5.16399900	-1.98400800	1.32071400
H	3.29447900	-0.94816800	1.51446300
C	5.93350300	-2.90117500	0.60197200
H	5.94231500	-4.17400000	-1.13066000
H	5.57848600	-1.53374200	2.22034200

C	7.31255800	-3.29900400	1.06347000
H	7.27714400	-4.18171300	1.71162700
H	7.78801000	-2.49485700	1.63141900
H	7.95842500	-3.54349000	0.21575700
O	-0.48497100	-1.83491700	-0.32348100
H	0.66911000	-2.35618700	-0.23678200
H	-4.33369029	-0.52244652	0.87738368
H	-3.41721819	-0.09068738	0.54271388

TS3B -275.54

Zero-point correction= 0.729533 (Hartree/Particle)

C	-1.51875700	0.37244600	-0.28071200
C	-1.86320600	1.23784000	0.72125000
H	-2.33519300	0.84974000	1.61404400
C	-1.42952700	2.62817600	0.69794400
C	-1.74923100	3.49753700	-0.49263000
C	-1.09139600	4.72814200	-0.60561400
C	-2.67556700	3.15632100	-1.47892400
C	-1.36078700	5.58638700	-1.66386800
H	-0.35859500	5.00975200	0.14684700
C	-2.94037700	4.01544800	-2.54306500
H	-3.19945300	2.20691000	-1.40935400
C	-2.29052300	5.24375300	-2.65083900
H	-0.83880300	6.53813400	-1.73072500
H	-3.66031100	3.72627200	-3.29554700
O	-0.78467100	0.79659000	-1.37111000
C	-2.05696400	-3.10699300	-0.99975500
N	-2.74015300	-3.04984700	0.09787900
N	-1.47526700	-1.90304800	-1.30695800

N	-2.60052300	-1.75258500	0.51781800
C	-1.83486300	-1.02116100	-0.32305900
C	-3.20685900	-1.35521900	1.75321500
C	-4.52412300	-0.89231400	1.72168400
C	-2.44749300	-1.43837300	2.92208000
C	-5.10342700	-0.49912700	2.92960800
C	-3.07337800	-1.03383800	4.10632900
C	-4.38840400	-0.56650100	4.12838200
C	-1.94084600	-4.27308600	-1.94323800
C	-0.56430600	-1.74554700	-2.45076700
C	-0.72492800	-2.93163800	-3.43028700
O	-0.78019100	-4.15722300	-2.71617700
C	0.60186700	-2.93332100	-4.19366600
H	0.81751700	-3.93126800	-4.58204500
H	0.56082100	-2.23787100	-5.03915100
C	1.58261800	-2.45913500	-3.14465800
C	2.96042900	-2.61747900	-3.08829200
C	0.91187200	-1.79943300	-2.11200500
C	3.65420900	-2.13142200	-1.98347100
H	3.48602800	-3.13326000	-3.88647800
C	1.59228200	-1.30505500	-0.99778200
C	2.97764200	-1.49063700	-0.94661400
H	4.73095400	-2.25521000	-1.92248300
H	1.07703000	-0.80732300	-0.17891800
H	3.52750000	-1.11770900	-0.08881100
H	-2.84058600	-4.31178700	-2.57579500
H	-1.88606700	-5.19903100	-1.37091800
H	-2.50668300	-1.07900600	5.03271900

H	-6.12454500	-0.12636400	2.93525500
C	-1.01211900	-1.88538600	2.87984900
H	-0.89632500	-2.78469700	2.26798100
H	-0.37826100	-1.09548100	2.45723800
H	-0.66186600	-2.10435200	3.88641800
C	-5.26218800	-0.78908400	0.41458200
H	-4.76012900	-0.07804900	-0.25156200
H	-5.29406700	-1.75584500	-0.09626100
H	-6.28491900	-0.44448700	0.57297300
H	-1.68922700	3.13899400	1.62775500
C	1.16988500	1.54271700	0.98943700
C	2.64747200	1.36988300	0.73218200
O	0.66621000	0.80961500	1.85491900
H	-0.22579400	2.49635100	0.68725400
H	-0.09686000	1.39400500	-1.02671300
C	-5.03655800	-0.16260500	5.42705800
H	-5.75727700	0.64388400	5.27460900
H	-5.57445900	-1.00865200	5.86685100
H	-4.29214700	0.17097800	6.15296000
C	3.48440600	0.98823700	1.71000100
C	4.94571700	0.85960200	1.63178400
C	5.64723200	0.90026700	0.41602000
C	5.69380100	0.66070200	2.80065400
C	7.02618800	0.76450000	0.37322400
H	5.09237500	1.03081300	-0.51331600
C	7.07694400	0.52636400	2.76363900
H	5.17040100	0.62466200	3.75152200
C	7.76308800	0.56995500	1.55621200

H	7.54693400	0.79373400	-0.58045100
H	7.62527300	0.37930600	3.68707600
H	3.03048500	0.74920500	2.67975100
H	3.01855700	1.67234100	-0.24295100
C	9.26308300	0.40656400	1.47759700
H	9.72715300	1.28988100	0.73366500
H	9.71471200	0.51860700	2.61199500
H	9.51537300	-0.70924000	1.01024200
C	-2.55971400	6.16750500	-3.81160700
H	-3.45960500	5.87952900	-4.35349800
H	-2.67486400	7.20200200	-3.47614800
H	-1.72395700	6.15893900	-4.53059100
H	-1.61284800	-2.82827200	-4.06881300
H	-0.81663400	-0.80957900	-2.95014300

M04BA

Zero-point correction= 0.589942 (Hartree/Particle)

C	-5.70719200	-2.66490100	0.13103100
C	-4.51911100	-1.96553400	0.29811600
C	-3.47188900	-2.49295400	1.07648300
C	-3.66501700	-3.75839400	1.65389200
C	-4.85350500	-4.45948300	1.48242800
C	-5.88645500	-3.91547500	0.72285500
H	-6.49815600	-2.23390500	-0.47623500
H	-4.39717200	-1.00589500	-0.19810900
H	-2.86508700	-4.18776800	2.25155000
H	-4.97337300	-5.43467000	1.94488900
C	-2.20811600	-1.78823700	1.29881100
H	-1.40613800	-2.35972600	1.76160800

C	-1.99438600	-0.48124800	0.99751300
C	-0.75345800	0.20635000	1.19316100
H	-2.81453800	0.08970100	0.57724900
O	0.29482600	-0.47612400	1.78205800
H	-0.92409500	-1.93633800	-0.61624400
C	0.86593900	-2.52041800	-0.92980700
O	1.68811400	-2.86935400	-1.78594600
O	-0.34853900	-2.08502400	-1.38647300
H	0.52472200	-1.27088300	1.21332600
O	1.04392300	-2.48904200	0.33072500
Cs	4.03785000	-2.49844200	0.05467300
C	0.49056300	3.46254200	0.43181900
N	-0.70329300	3.65958900	-0.00118300
N	0.66180800	2.22938800	1.01442300
N	-1.37328900	2.47324100	0.28548400
C	-0.52823000	1.51900800	0.84410500
C	-2.60862400	2.21656200	-0.37850800
C	-2.60070100	1.59462800	-1.63160400
C	-3.79661700	2.54329000	0.28441600
C	-3.83160700	1.27977700	-2.20917400
C	-5.00266700	2.21260500	-0.32902600
C	-5.03742700	1.57512400	-1.57203400
C	1.67741200	4.36841400	0.39707900
C	2.00625200	1.73374600	1.26344600
C	2.95146100	2.88637200	1.75563800
O	2.40517000	4.18436800	1.59582400
H	3.11548400	2.79810100	2.83038900
H	1.93057200	0.94970000	2.01469000

C	4.27627400	2.69684600	0.96933900
H	5.05146100	2.28318200	1.62343600
H	4.64066300	3.66705100	0.61692800
C	3.94152100	1.74928400	-0.15772800
C	4.70339700	1.39796000	-1.27053300
C	2.66210400	1.21995800	0.00206600
C	4.15396600	0.53364100	-2.22089200
H	5.69908000	1.80896800	-1.41269400
C	2.10506400	0.36262500	-0.94202700
C	2.86024300	0.02532800	-2.06626200
H	4.73194400	0.26843400	-3.10114100
H	1.09842500	-0.02497200	-0.81271700
H	2.44030100	-0.66159900	-2.79353600
H	2.29814000	4.14604300	-0.48435200
H	1.36180500	5.41062400	0.34991800
H	-5.93572400	2.44334500	0.17945500
H	-3.84614100	0.78086400	-3.17545600
C	-3.74516700	3.18275200	1.64530500
H	-3.16996300	4.11218900	1.61802600
H	-3.25020500	2.51781700	2.36080700
H	-4.75031600	3.39963500	2.01115500
C	-1.30912600	1.20949400	-2.30065700
H	-0.97078800	0.22744200	-1.94788900
H	-0.51807300	1.93473600	-2.09038800
H	-1.44253400	1.14375600	-3.38257000
C	-6.35361000	1.22595600	-2.21790400
H	-6.25347500	0.35528000	-2.87017000
H	-6.72051500	2.05758200	-2.82844100

H	-7.11538000	1.00795400	-1.46542300
C	-7.20421120	-4.68847063	0.52895717
H	-7.37524851	-4.84546057	-0.51555247
H	-8.01341148	-4.12313665	0.94186441
H	-7.13955705	-5.63389585	1.02583843

TS3BA -1423.48

Zero-point correction= 0.583993 (Hartree/Particle)

C	2.04086500	-0.13880800	1.16167600
C	4.74772300	-0.77686800	1.63117400
C	2.76729200	0.53064900	2.14987200
C	2.68093800	-1.12856500	0.42628900
C	4.02394200	-1.44394000	0.64738600
C	4.10564800	0.20940600	2.38669300
C	4.46646700	-2.54652300	-0.28946100
C	3.13919900	-3.17082600	-0.73307000
C	2.14371500	-1.98466300	-0.70264300
N	0.77656100	-2.47693300	-0.51380200
C	0.49047100	-3.75324200	-0.10367100
C	1.57999400	-4.77431100	-0.01572200
O	2.80545900	-4.13474200	0.25289700
N	-0.77145700	-3.91923600	0.12633300
N	-1.33004900	-2.69581900	-0.15435900
C	-0.40210600	-1.77541200	-0.52823300
C	-2.74247300	-2.53049300	-0.01389600
C	-5.46376000	-2.10633000	0.27503000
C	-3.53762600	-2.50324700	-1.16050400
C	-3.25886800	-2.35652400	1.27406700
C	-4.63935300	-2.15497000	1.39526300

C	-4.90411700	-2.29141700	-0.99948700
C	-2.92698600	-2.66250500	-2.52511200
C	-2.34955900	-2.35292100	2.46534400
H	1.00430700	0.12907700	0.98556100
H	5.78525800	-1.03424200	1.82732300
H	2.28021300	1.32388300	2.70719400
H	4.65219500	0.72848500	3.16851300
H	5.00210900	-2.14576500	-1.15847000
H	5.10150500	-3.30010000	0.18238200
H	3.18257700	-3.64900500	-1.72158600
H	2.15173400	-1.42229800	-1.63805400
H	1.63321300	-5.33794200	-0.95966800
H	1.37300600	-5.47101400	0.79716700
H	-5.06500200	-2.01949000	2.38515600
H	-5.54416800	-2.25979600	-1.87753800
H	-3.69156900	-2.78217300	-3.28251800
H	-2.25651400	-3.52615600	-2.55939800
H	-2.32987000	-1.77324800	-2.77973200
H	-1.82996200	-3.31033000	2.56449800
H	-1.57560000	-1.57473000	2.35572300
H	-2.90615700	-2.15784900	3.38306300
C	-0.52069800	-0.39519700	-0.90944900
C	-1.63660200	0.31596800	-0.51765500
H	-2.39531800	-0.19411200	0.07389600
C	-1.71444700	1.73619800	-0.68099100
H	-1.17588700	2.09828800	-1.55214500
C	-2.98355400	2.45594900	-0.41129800
C	-2.97618800	3.86107600	-0.41557500

C	-4.20483200	1.83240600	-0.10824200
C	-4.11341000	4.60034800	-0.14184200
H	-2.03835400	4.37799700	-0.64574100
C	-5.34354900	2.57919300	0.18785700
H	-4.27513100	0.74735300	-0.11859800
C	-5.32855200	3.97231300	0.17344300
H	-4.06873800	5.68744700	-0.16096800
H	-6.26741700	2.05624500	0.41430600
C	-6.55567800	4.77139200	0.49640200
H	-6.73317000	5.56139200	-0.24210600
H	-7.44367600	4.14451700	0.53614200
H	-6.46167500	5.26587000	1.47516800
O	0.52858000	0.13354500	-1.52185600
H	0.93120800	1.37273700	-1.07642900
C	1.26046200	2.75430900	0.41509800
O	2.14901100	3.45645600	0.91996900
O	0.16681600	2.42588800	1.01860000
H	-0.78557000	2.10350300	0.22187000
O	1.46016400	2.27828900	-0.82522800
Cs	4.41600500	2.18028800	-0.56368800
C	-6.93486500	-1.79479600	0.41625100
H	-7.12171200	-0.63922300	0.02402800
H	-7.56292300	-2.57717600	-0.29180200
H	-7.25178000	-1.90545600	1.59336100

M05BA

Zero-point correction= 0.591343 (Hartree/Particle)

C	-2.86297200	-3.46454000	-0.75316600
C	-2.24418900	-2.77796200	0.28570400

C	-2.98016100	-2.35509800	1.39789100
C	-4.34726600	-2.63022300	1.44577800
C	-4.96958400	-3.32540900	0.40806900
C	-4.22952700	-3.74511700	-0.69460800
H	-2.26536100	-3.76985000	-1.60726400
H	-1.18239600	-2.55179000	0.20941500
H	-4.93048800	-2.29660200	2.30127600
H	-6.03360100	-3.53862900	0.46277000
C	-2.29490800	-1.51580100	2.46088100
H	-2.89262400	-1.51548300	3.37974500
C	-2.12092400	-0.13064200	1.91008600
C	-0.92469400	0.30024300	1.42810800
H	-3.01934000	0.45069500	1.74171900
O	0.24859700	-0.22946700	1.51840200
C	1.49154300	-2.58325900	-0.80612000
O	2.48880000	-3.16485400	-1.28720200
O	0.46418000	-2.16516100	-1.37885200
O	1.59010000	-2.33535000	0.56252200
Cs	4.54315200	-1.99970500	0.48147000
C	-0.23855900	3.39499600	-0.35763200
N	-1.47142700	3.26636300	-0.74647000
N	0.12248000	2.39445900	0.49929500
N	-1.89376200	2.12489300	-0.12050000
C	-0.94207600	1.58063500	0.65321600
C	-3.15747800	1.52427900	-0.45221400
C	-3.13478600	0.39108600	-1.26583000
C	-4.31793600	2.04043800	0.12834100
C	-4.34848200	-0.27117900	-1.45390200

C	-5.50276500	1.34731300	-0.09836500
C	-5.52815900	0.17650200	-0.86398800
C	0.78281200	4.45053400	-0.62930900
C	1.51294600	2.24231300	0.92618000
C	2.17598900	3.64482200	1.15126700
O	1.43207600	4.72275600	0.59849900
H	2.22450300	3.87495500	2.21628900
H	1.50486400	1.62538900	1.82423100
C	3.58899500	3.52273000	0.52823500
H	4.33664200	3.37664300	1.31554600
H	3.84579500	4.45052600	0.00810000
C	3.51248100	2.32374600	-0.38674600
C	4.41203600	1.89473800	-1.36113500
C	2.33557300	1.61004200	-0.17401300
C	4.09619800	0.76386700	-2.11879800
H	5.33086500	2.44365200	-1.54741500
C	2.00382100	0.49046600	-0.92800000
C	2.89862500	0.06945900	-1.91263100
H	4.78104000	0.43324100	-2.89450200
H	1.08131300	-0.05687700	-0.75673900
H	2.65200600	-0.81514100	-2.49311500
H	1.48901700	4.10969300	-1.39937400
H	0.30270500	5.36892300	-0.96636100
H	-6.42219200	1.71312100	0.35283600
H	-4.35819900	-1.17974300	-2.04918900
C	-4.25730100	3.25948400	1.00949600
H	-3.84101100	4.11622500	0.47229800
H	-3.61612400	3.07682800	1.87932500

H -5.25251600 3.52406900 1.36992200

C -1.85799700 -0.12126700 -1.86934600

H -1.24318400 -0.66777400 -1.14443900

H -1.25371200 0.69591900 -2.27836000

H -2.07264600 -0.82574400 -2.67469700

C -6.81280100 -0.59182000 -1.03213800

H -6.66318400 -1.47251500 -1.65996800

H -7.59101100 0.03047200 -1.48410900

H -7.18404500 -0.93244200 -0.06053500

H -1.30895000 -1.93138200 2.69086900

H 0.90045800 -1.68256100 0.82850300

C -4.91608388 -4.51158518 -1.84036809

H -5.97710712 -4.47858586 -1.70605227

H -4.58745204 -5.52985694 -1.83549598

H -4.66071557 -4.05885857 -2.77563577

TS3BA1 -1370.72

Zero-point correction= 0.583552 (Hartree/Particle)

C -2.07158800 0.16160400 1.14765600

C -4.79256600 0.77326300 1.57125800

C -2.79929100 -0.49154200 2.14598200

C -2.71775300 1.12220800 0.37973600

C -4.06685000 1.42562700 0.57902900

C -4.14519300 -0.18430800 2.35845500

C -4.51272700 2.49956300 -0.38900200

C -3.18828500 3.13096200 -0.83042300

C -2.17972600 1.95740300 -0.76429200

N -0.81915500 2.46891600 -0.57368900

C -0.55406900 3.75678000 -0.18517100

C	-1.65642800	4.76613800	-0.12889200
O	-2.87695200	4.11791400	0.14002300
N	0.70328900	3.94401200	0.05370700
N	1.27979600	2.72380300	-0.19851700
C	0.36951900	1.78417100	-0.56708400
C	2.68582900	2.55015800	-0.00579400
C	5.37864600	2.02367000	0.36408600
C	3.52165600	2.54123200	-1.12332700
C	3.14871900	2.32500800	1.29333000
C	4.50830100	2.06928900	1.45597800
C	4.87334600	2.27588200	-0.91200400
C	2.95757300	2.74705400	-2.50184400
C	2.18385800	2.29707300	2.44743200
H	-1.03013700	-0.09746200	0.98690200
H	-5.83570700	1.02066400	1.74946600
H	-2.30816700	-1.26270400	2.73052400
H	-4.69320400	-0.69074600	3.14762600
H	-5.03165900	2.07086000	-1.25466300
H	-5.16382100	3.25509200	0.05710100
H	-3.22575000	3.58895400	-1.82869000
H	-2.17343900	1.37368800	-1.68615100
H	-1.70622200	5.30956300	-1.08485200
H	-1.46594300	5.48197900	0.67128900
H	4.89357300	1.87927400	2.45494700
H	5.54364300	2.24882400	-1.76736400
H	3.75749800	2.86054000	-3.23506800
H	2.31867800	3.63408800	-2.53959100
H	2.34773100	1.88256700	-2.78750700

H	1.64297600	3.24391900	2.53290300
H	1.43976400	1.50518900	2.30128500
H	2.70647700	2.10480400	3.38556200
C	0.50606300	0.39518800	-0.92071400
C	1.64595900	-0.28875500	-0.55388300
H	2.44099900	0.23839900	-0.04072800
C	1.74976500	-1.71396600	-0.70226600
H	1.22762000	-2.09819000	-1.58382000
C	3.04343200	-2.37721400	-0.42522500
C	3.39854900	-3.55537800	-1.09918700
C	3.95187200	-1.89901900	0.53317900
C	4.59301900	-4.21526400	-0.83650000
H	2.71942200	-3.95287500	-1.84951400
C	5.14955000	-2.55858800	0.78868700
H	3.71716500	-1.00115400	1.10012400
C	5.49594500	-3.73038300	0.11294800
H	4.83432000	-5.12337200	-1.38505700
H	5.82911500	-2.15788000	1.53859000
C	6.77771100	-4.46267700	0.41964300
H	7.53563800	-3.78340600	0.81920800
H	6.61901100	-5.25062900	1.16430600
H	7.18759100	-4.93854100	-0.47565400
O	-0.54816100	-0.16100400	-1.49694300
H	-0.92064800	-1.41216300	-1.04364100
C	-1.19789800	-2.73246700	0.48844600
O	-2.06144600	-3.43027600	1.03994700
O	-0.10379500	-2.34591500	1.05498800
H	0.83316400	-2.07323100	0.22268600

O	-1.42528400	-2.32079900	-0.77063900
C	6.82726000	1.66031600	0.55721200
H	6.95116600	0.57366600	0.50143400
H	7.45583000	2.10581400	-0.21707900
H	7.19287900	1.98833600	1.533031

M3

Zero-point correction= 0.560087 (Hartree/Particle)

C	-4.00087500	-3.43341700	0.58097500
C	-2.77313400	-3.00907300	0.08738900
C	-2.70036500	-2.04478400	-0.92566100
C	-3.90292800	-1.54376600	-1.43892000
C	-5.12871600	-1.96730900	-0.94051700
C	-5.20023900	-2.91893400	0.07961400
H	-4.03305900	-4.18278000	1.36834900
H	-1.85828500	-3.43600600	0.48891600
H	-3.86746900	-0.78963700	-2.22113400
H	-6.04759100	-1.55097600	-1.34606600
C	-1.42872800	-1.48221400	-1.40356100
H	-1.62960379	-0.45369642	-1.61966399
C	-0.26950300	-1.49340800	-0.74497800
C	-6.53161800	-3.39917800	0.59772600
H	-6.95047200	-4.17243100	-0.05498500
H	-6.43650700	-3.82785500	1.59824500
H	-7.25624100	-2.58179400	0.64213700
C	0.94089100	-0.74640000	-1.28708900
H	-0.15449000	-1.99993500	0.21568800
O	0.79007300	-0.09242300	-2.43167100
C	-2.18724800	0.46757200	1.08661600

C	-4.10123800	2.47755800	0.63510300
C	-3.44165300	0.40114900	1.69315200
C	-1.91046000	1.53209800	0.24138200
C	-2.85162200	2.54465000	0.03514700
C	-4.39459500	1.39063900	1.45995600
C	-2.27922900	3.62467400	-0.85645900
C	-0.77559100	3.36589300	-0.75354900
C	-0.68905300	1.83298100	-0.60541500
N	0.61782300	1.48812200	-0.04562100
C	1.45014000	2.39006500	0.55771100
C	1.02630700	3.82034600	0.72713300
O	-0.34604200	3.97276600	0.46222200
N	2.62151800	1.87451400	0.78957000
N	2.51722300	0.60078500	0.28648600
C	1.32465700	0.35530100	-0.25273200
C	3.65756900	-0.27220700	0.28384900
C	5.85182800	-1.94967100	0.26431200
C	4.42211600	-0.34332300	-0.88390600
C	3.94236800	-1.00107500	1.43796800
C	5.05532700	-1.83975200	1.40487600
C	5.52057500	-1.20065200	-0.86710600
C	4.05914000	0.47539900	-2.09441300
C	3.06760800	-0.88012000	2.65686200
C	7.06594200	-2.84235700	0.25951400
H	-1.45156200	-0.30926700	1.26497600
H	-4.83401300	3.26329400	0.47630800
H	-3.67961700	-0.43915600	2.33748000
H	-5.36880500	1.32110100	1.93354600

H	-2.60836300	3.50641500	-1.89510000
H	-2.52290000	4.63858200	-0.53065900
H	-0.18705200	3.74363300	-1.59975900
H	-0.71270600	1.32224200	-1.57818800
H	1.63501100	4.43492200	0.04787900
H	1.20450300	4.15628600	1.75029300
H	5.30221500	-2.42309800	2.28833400
H	6.13261600	-1.28431000	-1.76176100
H	4.76414000	0.28427600	-2.90522200
H	4.09932300	1.54322100	-1.85214400
H	3.04036900	0.25964300	-2.44656400
H	3.42891800	-1.52673700	3.45774200
H	3.04850700	0.15061800	3.02299100
H	2.03455200	-1.16245000	2.42725500
H	6.97193300	-3.64174800	0.99766000
H	7.96610100	-2.26872800	0.50285400
H	7.21866700	-3.29489900	-0.72295400
H	-1.23432506	-1.95998208	-2.34102842

RR-TS4 -289.65

Zero-point correction= 0.810814 (Hartree/Particle)

C	-0.30074500	-0.15721200	-0.68863900
C	-0.55307000	2.40352400	-0.57811900
C	1.61213000	1.71188100	-1.43782300
C	0.37782900	2.45453600	-1.59132900
C	1.15594100	-0.22168900	-0.82577100
O	-0.34908900	1.56833600	0.39449800
O	-0.99916400	-0.04218600	-1.71157600
Si	2.87333300	2.38185000	-0.19171400

C	3.82501500	3.69825500	-1.16226600
C	4.12971300	1.10395400	0.39026200
C	2.09238300	3.19703500	1.30802300
C	1.63261700	-1.07522600	-1.99829900
C	3.13679100	-1.18434600	-2.04727800
C	5.96306400	-1.35861000	-2.03562500
C	3.90144500	-0.49684900	-2.98839100
C	3.80877100	-1.97277200	-1.10693100
C	5.19419900	-2.05865100	-1.09859100
C	5.29410400	-0.57986800	-2.97957000
C	-1.86109200	3.11659200	-0.61746000
C	-4.34633400	4.43414100	-0.59342500
C	-2.12785300	4.16053900	-1.51240600
C	-2.85452200	2.76090100	0.30320500
C	-4.08530900	3.41185200	0.31590000
C	-3.35917200	4.80610800	-1.50587900
H	2.10954400	1.52561200	-2.39463400
H	0.14205500	2.92567100	-2.54000500
H	1.72397200	-0.35368500	0.09038900
H	4.58674100	4.17622300	-0.53765500
H	3.15034800	4.47837600	-1.52864700
H	4.33030400	3.25547000	-2.02725100
H	4.82995700	1.58868600	1.08148100
H	4.70411900	0.69554200	-0.44741300
H	3.66397100	0.26706300	0.92010400
H	2.84271200	3.79806900	1.83310000
H	1.25992300	3.84516000	1.02214600
H	1.70714400	2.43996100	1.99196400

H	1.21259300	-2.08983400	-1.91612300
H	1.23704800	-0.65805900	-2.92775600
H	3.40469400	0.10997700	-3.74133500
H	3.22981700	-2.51544100	-0.36234100
H	5.69294200	-2.67350400	-0.35262800
H	5.86872700	-0.03153400	-3.72156300
H	-5.30354900	4.94560100	-0.58473500
H	-1.35924000	4.48087800	-2.20893100
H	-2.64171700	1.97118300	1.01841700
H	-4.83696900	3.12077400	1.04518400
H	-3.54690900	5.61176600	-2.20902400
C	7.46713400	-1.45913600	-2.02243900
H	7.79572900	-2.47595500	-2.25912600
H	7.91493300	-0.78265900	-2.75351400
H	7.86951000	-1.20787600	-1.03675200
C	-1.05626700	-0.59646000	0.54000100
N	-0.65042700	-0.96539300	1.76169700
N	-2.40503300	-0.69600300	0.58029900
N	-1.69926800	-1.25540300	2.59459200
C	0.68224800	-1.00558100	2.29234500
C	-2.74420700	-1.08908200	1.84581500
C	-3.43147200	-0.50488400	-0.46206400
C	1.48892000	-2.10211200	1.97824600
C	1.08777100	0.03199900	3.13348800
C	-4.19247200	-1.14258200	2.19815600
H	-3.05071400	0.26139200	-1.13424200
C	-4.80755000	-0.08227700	0.17733900
C	-3.72071000	-1.81009500	-1.16812100

C	2.78989900	-2.10174700	2.47418500
C	0.95993000	-3.22402400	1.12550000
C	2.40139400	-0.01496500	3.60454300
C	0.13575700	1.12837400	3.52753100
H	-4.31841200	-1.06480500	3.27781200
H	-4.64780900	-2.07997700	1.84846500
O	-4.79032100	-0.01317400	1.59484300
C	-5.85779800	-1.08925200	-0.35406100
H	-5.03428200	0.93626600	-0.13640200
C	-5.07023800	-2.14305400	-1.08771000
C	-2.80941800	-2.63193600	-1.82654000
C	3.26721800	-1.05609900	3.27000500
H	3.44700000	-2.93461700	2.23393500
H	0.72861500	-2.87631100	0.11207200
H	0.03712200	-3.63391000	1.54725500
H	1.69224200	-4.02975600	1.04850400
H	2.75575800	0.79055900	4.24288800
H	-0.67177600	0.72296900	4.14477800
H	-0.30692400	1.58568000	2.63604400
H	0.65690700	1.89712600	4.10111500
H	-6.44731800	-1.49298300	0.47620300
H	-6.56120700	-0.58569200	-1.02398900
C	-5.53355300	-3.32305300	-1.66553100
C	-3.27491400	-3.81274400	-2.39771000
H	-1.76743500	-2.33594100	-1.90328700
C	4.70454900	-1.03785500	3.71723400
C	-4.62717600	-4.15629300	-2.31535000
H	-6.58462800	-3.59020500	-1.60779200

H	-2.58451200	-4.46905200	-2.91730100
H	5.33400400	-0.61656200	2.92554100
H	4.83642200	-0.42473600	4.61103100
H	5.06826400	-2.04605000	3.92942100
H	-4.97438200	-5.08040800	-2.76635800

RS-TS4 -216.21

Zero-point correction= 0.809661 (Hartree/Particle)

C	0.06043400	0.32094900	-0.31729700
C	-0.55614100	2.84067300	-0.26384900
C	1.80029900	2.54975500	-0.41433100
C	0.61253000	3.16044000	-0.93188700
C	1.49872200	0.49422500	-0.56233200
O	-0.48834700	1.99988200	0.71405400
O	-0.73528500	0.30800600	-1.27860000
C	1.90914300	0.10466000	-1.97373600
C	1.90077900	-1.39581800	-2.15734400
C	1.88040500	-4.21771200	-2.39060500
C	3.00949200	-2.15766300	-1.78203800
C	0.77982100	-2.06592800	-2.66012400
C	0.77142100	-3.45376500	-2.76879300
C	3.00104800	-3.54552400	-1.89982100
C	-1.89949500	3.35266800	-0.65553000
C	-4.46111300	4.31354600	-1.28025100
C	-2.19703200	3.79702200	-1.94821900
C	-2.90623600	3.38821600	0.31603200
C	-4.17633200	3.86809300	0.00917400
C	-3.46717400	4.27348500	-2.25696300
H	0.61426500	3.75382500	-1.84088500

H	2.18773900	0.16034800	0.20648900
H	1.22706600	0.58474600	-2.67990900
H	2.91967800	0.47039300	-2.17331800
H	3.89075500	-1.65629400	-1.38623100
H	-0.08704200	-1.48212500	-2.95627400
H	-0.10801900	-3.95564900	-3.16684700
H	3.87772000	-4.11704700	-1.60323500
H	-5.45085000	4.68641700	-1.52396700
H	-1.43792900	3.74081300	-2.72189400
H	-2.66417200	3.03382400	1.31425800
H	-4.94323500	3.90193700	0.77820200
H	-3.68648200	4.60527100	-3.26709700
C	1.86388500	-5.71895200	-2.52892200
H	1.88880100	-6.01780300	-3.58170800
H	2.72588100	-6.17159900	-2.03349500
H	0.95694500	-6.14573700	-2.09105000
C	-0.46695000	-0.28932700	0.95958400
N	0.15572500	-0.78348100	2.03307400
N	-1.77756700	-0.45883700	1.21744200
N	-0.71649000	-1.24837900	2.98117600
C	1.57135400	-0.90962700	2.22163900
C	-1.88289900	-1.04304300	2.44589100
C	-2.96647100	-0.15378400	0.41678800
C	2.19110500	-2.04471300	1.70016000
C	2.25559900	0.14311500	2.83203300
C	-3.25716700	-1.22719600	2.99795500
H	-2.69843000	0.66756200	-0.24916000
C	-4.17971400	0.22326800	1.34277000

C	-3.42784700	-1.38703800	-0.32739900
C	3.58384800	-2.08753000	1.75682900
C	1.37579500	-3.13480800	1.05930600
C	3.64482400	0.04764800	2.87094200
C	1.50861100	1.32580500	3.38650200
H	-3.21624900	-1.35820500	4.07898100
H	-3.74483600	-2.10152500	2.54414900
O	-3.95897200	-0.03100300	2.72399400
C	-5.38294800	-0.57414700	0.78518400
H	-4.35209100	1.29795700	1.29067600
C	-4.78136400	-1.62788800	-0.10929200
C	-2.65566400	-2.23356600	-1.11631500
C	4.32230900	-1.04384800	2.31761800
H	4.10002900	-2.94767500	1.33737100
H	0.95231900	-2.79349200	0.10558100
H	0.54987900	-3.43767000	1.70963500
H	1.99641800	-4.00624800	0.84661900
H	4.21498300	0.85276700	3.32871900
H	0.87722800	1.02015400	4.22692700
H	0.85315100	1.76751000	2.62637100
H	2.20608500	2.08767400	3.73913400
H	-5.97206100	-0.98652800	1.61039300
H	-6.04235900	0.08966200	0.21633900
C	-5.39630200	-2.73097400	-0.69621200
C	-3.27207500	-3.33978100	-1.69537700
H	-1.60146100	-2.02320400	-1.27335700
C	5.82826900	-1.06751100	2.30142800
C	-4.63224900	-3.58445800	-1.48884700

H	-6.45143200	-2.92833800	-0.53153500
H	-2.69197200	-4.01853900	-2.31228700
H	6.20566900	-0.42522000	1.49805200
H	6.24020900	-0.69376300	3.24199400
H	6.20963900	-2.07653900	2.13261700
H	-5.09706100	-4.45179800	-1.94682800
H	1.76566500	2.46925800	0.67600400
Si	3.52027700	3.00212700	-1.01724400
C	4.75886200	1.73578600	-0.36302600
H	4.61311400	0.73555900	-0.78202600
H	5.78630800	2.04741700	-0.57596900
H	4.64836600	1.65316300	0.72450000
C	3.95190900	4.67243200	-0.25233800
H	3.92720200	4.62172500	0.84048000
H	4.95329000	4.99659300	-0.55344200
H	3.23777000	5.43932700	-0.56681200
C	3.60165900	3.18572200	-2.89070800
H	2.90815300	3.96631300	-3.21981400
H	4.60763600	3.48976800	-3.19712400
H	3.34729100	2.26760000	-3.42492000

SR-TS4 -230.50

Zero-point correction= 0.810108 (Hartree/Particle)

C	-0.07286900	0.23007600	0.29334900
C	-2.38768300	0.10336600	1.64227000
C	-2.49341500	1.72928000	-0.09368600
C	-2.77286100	1.38109500	1.26696800
C	-0.42051900	1.55098400	-0.25507600
O	-1.81514300	-0.65940000	0.77952200

C	0.17396400	2.70307300	0.54046400
C	1.68868300	2.71975000	0.49975400
C	4.51140700	2.54817700	0.32119500
C	2.36471900	3.14344700	-0.64763900
C	2.45026200	2.22931700	1.56777000
C	3.83356500	2.13757900	1.47450300
C	3.75525900	3.05855400	-0.73704000
C	-2.50408200	-0.38124900	3.04633200
C	-2.67412500	-1.32969800	5.67259800
C	-1.52726800	-1.24594600	3.54964400
C	-3.56865600	-0.00060700	3.86766800
C	-3.65274600	-0.47196200	5.17500200
C	-1.61011400	-1.71367500	4.85639000
H	-3.06229500	2.11937900	2.00862800
H	-0.30133800	1.62942900	-1.33736100
H	-0.20713800	3.64964100	0.14188300
H	-0.17092300	2.61265700	1.57561800
H	1.79537600	3.53135700	-1.49044000
H	1.93786800	1.87004100	2.45316800
H	4.40138200	1.72432000	2.30499700
H	4.25935200	3.39674600	-1.63973400
H	-2.74048700	-1.69835000	6.69153000
H	-0.69651600	-1.50396700	2.89914900
H	-4.34118000	0.65066900	3.46850200
H	-4.48722500	-0.17668500	5.80377100
H	-0.84164200	-2.37534300	5.24494200
O	0.56800200	0.09067100	1.35318400
C	6.01633500	2.47319600	0.25963200

H	6.47078500	3.23302000	0.90330700
H	6.37319500	1.49763400	0.60708100
H	6.38501800	2.63694300	-0.75654000
C	0.21060000	-0.85363500	-0.75484900
N	-0.50395300	-1.72673700	-1.47197200
N	1.49337600	-1.10482500	-1.08587900
N	0.29420400	-2.52432100	-2.25335100
C	-1.93013100	-1.89634100	-1.63635200
C	1.50170400	-2.12452700	-1.98960600
C	2.72734100	-0.40533500	-0.71977900
C	-2.57181100	-2.88714100	-0.89689500
C	-2.57104900	-1.12035700	-2.60161400
C	2.81030400	-2.43152900	-2.63782400
H	2.44440300	0.58503800	-0.36088800
C	3.66544900	-0.28849200	-1.97548300
C	3.55759700	-1.12911200	0.31339800
C	-3.93512200	-3.06424600	-1.12374600
C	-1.82503200	-3.68889300	0.13043500
C	-3.93678000	-1.32934900	-2.78397200
C	-1.82271100	-0.09159600	-3.40737200
H	2.66210700	-3.03649500	-3.53157600
H	3.48302200	-2.95520900	-1.94581100
O	3.33018900	-1.17577700	-3.03790800
C	5.08882400	-0.54676400	-1.43712300
H	3.56929400	0.70754100	-2.41024100
C	4.89213700	-1.18076800	-0.08338300
C	3.13856800	-1.63239000	1.54043300
C	-4.63363300	-2.29170400	-2.05153100

H	-4.46552600	-3.82082200	-0.55148000
H	-1.53079400	-3.02146800	0.94616200
H	-0.92275600	-4.13824900	-0.29549600
H	-2.45369400	-4.48649400	0.52939300
H	-4.46529000	-0.73234700	-3.52363700
H	-0.90318100	-0.50680600	-3.83110600
H	-1.54627300	0.76655100	-2.78580900
H	-2.44235600	0.27641500	-4.22720300
H	5.64092800	-1.17963300	-2.13876300
H	5.63317300	0.39911300	-1.35085800
C	5.84572200	-1.74870500	0.75727400
C	4.09537500	-2.20994600	2.37246600
H	2.09636200	-1.54185300	1.83099500
C	-6.10964900	-2.49446700	-2.27791100
C	5.43659300	-2.26558700	1.98522700
H	6.88910200	-1.79414000	0.45882700
H	3.79779400	-2.61244900	3.33520500
H	-6.51810300	-3.23414100	-1.58682500
H	-6.65673100	-1.55807600	-2.13739500
H	-6.30475800	-2.83813400	-3.29823800
H	6.16822400	-2.71612100	2.64844000
Si	-3.02576500	3.37907500	-0.81626600
H	-2.65659800	0.88311100	-0.76745800
C	-4.78270900	3.15714100	-1.46614500
H	-5.46306000	2.87134300	-0.65863200
H	-5.15990100	4.07874400	-1.92051000
H	-4.81499300	2.36711800	-2.22372600
C	-1.93193000	3.87117100	-2.27385300

H	-0.89111300	4.03929000	-1.98209200
H	-1.94459900	3.09774400	-3.04912200
H	-2.30417400	4.79539900	-2.72744200
C	-3.01755000	4.72542200	0.50094400
H	-3.75484100	4.50338800	1.27858100
H	-2.04262200	4.82468800	0.98598300
H	-3.27810900	5.69401000	0.06294800

SS-TS4 -240.79

Zero-point correction= 0.810833 (Hartree/Particle)

C	0.18189200	0.49410600	-0.65821100
C	2.68566900	1.50766000	-0.77290000
C	1.11882300	3.25358300	-0.41661700
C	2.23381800	2.75090100	-1.16450400
C	-0.39794100	1.85336300	-0.66201400
O	2.04349600	0.89414200	0.16668100
Si	1.30001200	3.40173700	1.47016100
C	3.12204700	3.40126800	1.95128400
C	0.34510500	2.20849700	2.58453800
C	0.60017600	5.11676100	1.87215900
C	-1.01584700	2.27835100	-1.99076000
C	-2.45648100	1.83555700	-2.09538000
C	-5.13296300	0.91231200	-2.03697200
C	-3.45584000	2.56117000	-1.43500000
C	-2.82314300	0.65724900	-2.74966000
C	-4.14318500	0.21194400	-2.72892900
C	-4.76854100	2.10127800	-1.39524400
C	3.83024000	0.84064700	-1.45262300
C	5.99041800	-0.43605100	-2.69365900

C	3.77369200	-0.52367700	-1.73994400
C	4.97907000	1.56191700	-1.79244300
C	6.05342700	0.92714700	-2.41072400
C	4.84499200	-1.15840300	-2.35844300
H	0.63505900	4.13048900	-0.85509000
H	2.58656000	3.22221400	-2.07585200
H	-0.99777600	2.09844800	0.21312800
H	3.23662500	3.39048000	3.04031000
H	3.61300400	4.30170200	1.56935800
H	3.64170400	2.53422500	1.53636600
H	0.51460600	2.51311800	3.62368300
H	-0.73749500	2.23043700	2.41804600
H	0.69932500	1.18115300	2.47969200
H	1.12862700	5.89909900	1.31787500
H	-0.46081600	5.18100700	1.60547000
H	0.68790800	5.34438100	2.93940300
H	-0.97683000	3.36720000	-2.08812700
H	-0.41372300	1.84603000	-2.79351700
H	-3.19267600	3.48892400	-0.93059200
H	-2.05731500	0.07972100	-3.26103600
H	-4.40388200	-0.71663900	-3.23134900
H	-5.52704300	2.67769400	-0.87023800
H	6.82851500	-0.93196800	-3.17402400
H	2.86457400	-1.05921600	-1.49251000
H	5.03029500	2.61973100	-1.54847500
H	6.94421700	1.49418000	-2.66370800
H	4.78343000	-2.22059600	-2.58207500
O	0.44384200	-0.12872300	-1.70246200

C	-6.54512800	0.39092400	-1.96318700
H	-6.82757700	-0.11731900	-2.88851400
H	-6.64733400	-0.33722700	-1.14997200
H	-7.25877100	1.19866300	-1.78331600
C	-0.09484300	-0.34966200	0.59270900
N	0.67263800	-1.21097900	1.27495000
N	-1.29571300	-0.39780900	1.21290200
N	0.00644400	-1.76067600	2.33889100
C	1.95223400	-1.81033500	0.96062900
C	-1.18371700	-1.25110900	2.27373300
C	-2.64271500	-0.05705800	0.74087000
C	1.95144500	-2.84626500	0.02667400
C	3.07977800	-1.45287100	1.70368200
C	-2.38372900	-1.42279100	3.15085100
H	-2.58293200	0.78347200	0.05313300
C	-3.58306600	0.26741500	1.94521600
C	-3.27560300	-1.26131800	0.08326600
C	3.16629200	-3.48967000	-0.21935900
C	0.70633500	-3.22751100	-0.72566500
C	4.26621100	-2.11711400	1.41160700
C	3.01747000	-0.39016400	2.76178400
H	-2.07383400	-1.66449200	4.16759100
H	-3.02392700	-2.23010300	2.76986200
O	-3.05955900	-0.18111500	3.18802000
C	-4.92572700	-0.40796500	1.58052900
H	-3.68968500	1.34638000	2.06849400
C	-4.56376300	-1.46474900	0.56514500
C	-2.71078000	-2.08149200	-0.88492200

C	4.33380600	-3.12234100	0.44371700
H	3.19487400	-4.28891700	-0.95598200
H	0.45480500	-2.43144200	-1.43558700
H	-0.14452500	-3.36728400	-0.04936900
H	0.86025900	-4.15857700	-1.27371300
H	5.16626100	-1.83902300	1.95427800
H	2.20414000	-0.58376200	3.46837200
H	2.83326500	0.57608300	2.28587200
H	3.95548900	-0.34632400	3.31811600
H	-5.41113400	-0.79793800	2.47972400
H	-5.59940800	0.33210300	1.13152100
C	-5.32509600	-2.52134400	0.07091600
C	-3.47067900	-3.14218000	-1.37138400
H	-1.71428900	-1.87387300	-1.26931000
C	5.65317900	-3.76451400	0.10802700
C	-4.76808800	-3.35655500	-0.89682600
H	-6.33305300	-2.69657700	0.43575400
H	-3.05698200	-3.80097100	-2.12772800
H	6.24270000	-3.09095800	-0.52380400
H	6.23759800	-3.96853500	1.00883700
H	5.51428900	-4.70173200	-0.43550300
H	-5.34857300	-4.18741300	-1.28463700

RR-M4

Zero-point correction= 0.813956 (Hartree/Particle)

C	0.06383800	-0.67489800	-0.76767100
C	-2.09142900	-0.51891800	-1.74395000
C	-0.81250000	1.59140800	-1.53134900
C	-1.77490800	0.68161500	-2.23912800

C	0.12410800	0.86163300	-0.52865100
O	-1.41887300	-1.02643900	-0.66452200
O	0.63432400	-1.13130600	-1.81216400
Si	-1.96183600	2.84602500	-0.64187400
C	-2.64975100	4.03844300	-1.93131800
C	-1.12992700	3.84918800	0.71986900
C	-3.34006900	1.84058700	0.16289700
C	1.58549100	1.32072000	-0.66927300
C	1.84940100	2.78044900	-0.39243400
C	2.31032500	5.52956900	0.14647500
C	1.76332800	3.74196500	-1.40370000
C	2.20521200	3.22093500	0.88649300
C	2.42719300	4.56680300	1.15336700
C	1.98012700	5.09224000	-1.13713700
C	-3.20783900	-1.35406000	-2.23594000
C	-5.39181700	-2.87297800	-3.11658400
C	-3.42951000	-1.55128700	-3.60200900
C	-4.09520200	-1.92441000	-1.31598100
C	-5.17866900	-2.67897400	-1.75268100
C	-4.51634300	-2.30378400	-4.03866900
H	-0.21487700	2.13103700	-2.27476900
H	-2.32574300	1.06334600	-3.09387500
H	-0.21627700	1.05471400	0.49778500
H	-3.36828100	4.73056800	-1.48141600
H	-3.15974500	3.50613500	-2.73975200
H	-1.84284700	4.63329200	-2.37188600
H	-1.88903800	4.47379300	1.20468600
H	-0.33451300	4.49905500	0.34525400

H	-0.69892000	3.19633900	1.48587700
H	-3.82316400	2.40392500	0.96838700
H	-4.09990700	1.54231800	-0.56481200
H	-2.91819500	0.92261100	0.59079100
H	2.21984000	0.71132300	-0.00799300
H	1.88784600	1.07549400	-1.69027300
H	1.53517500	3.43094600	-2.41967300
H	2.31681900	2.49390100	1.68644600
H	2.69920800	4.87852400	2.15907200
H	1.90025600	5.81738000	-1.94311800
H	-6.23538000	-3.46419000	-3.45848300
H	-2.73048700	-1.12677900	-4.31614600
H	-3.93713700	-1.74030900	-0.25721900
H	-5.86370000	-3.11075700	-1.02906500
H	-4.67323600	-2.45613000	-5.10206700
C	2.57040100	6.98582100	0.43671600
H	3.64303300	7.18065400	0.53812300
H	2.19105900	7.62250600	-0.36563300
H	2.09302200	7.29344000	1.37099900
C	0.54315100	-1.47188000	0.51250600
N	-0.01406900	-1.55255600	1.72344500
N	1.54498300	-2.38639000	0.52314000
N	0.55392300	-2.51765400	2.49710200
C	-1.16245800	-0.86507100	2.25853400
C	1.48368400	-3.01967000	1.74232200
C	2.47155700	-2.84221700	-0.57077400
C	-0.98145600	0.35504000	2.90520000
C	-2.39292400	-1.52997400	2.20252100

C	2.29937200	-4.22754000	2.08512200
H	1.82230500	-3.17031100	-1.37960900
C	3.38037100	-3.95885100	0.02422800
C	3.52540600	-1.84782800	-1.02671600
C	-2.11939200	0.97829000	3.42450500
C	0.38893700	0.94055300	3.12080400
C	-3.49239500	-0.87381600	2.74615200
C	-2.49144300	-2.91395200	1.62104300
H	1.65771500	-4.93322000	2.61475400
H	3.13453700	-3.96506800	2.74526100
O	2.73247100	-4.85031300	0.90214600
C	4.61270900	-3.22738300	0.59663300
H	3.70281200	-4.56698600	-0.82411200
C	4.73531600	-2.07565400	-0.36097900
C	3.46245400	-0.93227400	-2.07411900
C	-3.37767000	0.38771500	3.34008700
H	-2.01075400	1.94420200	3.91172400
H	1.06626500	0.71899000	2.29282200
H	0.83330700	0.52262900	4.03039800
H	0.33617400	2.02536700	3.23803900
H	-4.46486100	-1.35895000	2.70688700
H	-1.91058800	-3.61998300	2.22282500
H	-2.10135800	-2.92568000	0.59946400
H	-3.52953600	-3.25178000	1.60453700
H	4.44124700	-2.86575300	1.61689600
H	5.49342000	-3.87304000	0.62071600
C	5.87089200	-1.33698700	-0.67121100
C	4.60275700	-0.19319900	-2.38477500

H	2.52566200	-0.80167700	-2.60091700
C	-4.60167800	1.09718700	3.85476800
C	5.79214100	-0.37946200	-1.68013600
H	6.80685800	-1.52004500	-0.15198200
H	4.56432600	0.53245400	-3.19103200
H	-5.15144100	1.55067500	3.02323000
H	-5.27983100	0.40295600	4.35722500
H	-4.33676000	1.89185900	4.55515100
H	6.66940500	0.20597800	-1.93710700

RS-M4

Zero-point correction= 0.813685 (Hartree/Particle)

C	0.43827200	0.30554700	0.48293100
C	0.06525700	2.75430200	0.35975900
C	-1.51447100	1.31664600	1.60955700
C	-1.04667400	2.65021700	1.10641800
C	-1.09490900	0.26257100	0.57367900
O	0.84282000	1.68568000	0.01961300
O	1.05359200	-0.09276400	1.54071100
C	-1.56556700	-1.15769700	0.89215500
C	-3.06810100	-1.30858900	0.95811600
C	-5.88291700	-1.62897100	1.06375700
C	-3.67709100	-1.99920000	2.00597500
C	-3.89316100	-0.78288200	-0.04358900
C	-5.27268700	-0.94046300	0.00976000
C	-5.06127800	-2.15854100	2.05766100
C	0.59914300	4.02358300	-0.18764500
C	1.64611700	6.41712100	-1.21720100

C	1.94985200	4.11833600	-0.54246200
C	-0.22430200	5.14078300	-0.37946500
C	0.29612400	6.32764300	-0.88134000
C	2.46821200	5.30621300	-1.04926300
H	-1.57077500	3.55521400	1.39488900
H	-1.52139800	0.57608600	-0.38634100
H	-1.18032300	-1.83697300	0.11847700
H	-1.11028200	-1.47769200	1.83180200
H	-3.05827700	-2.43039200	2.78873600
H	-3.45025900	-0.24658400	-0.87911300
H	-5.89166500	-0.51726200	-0.77854200
H	-5.50829500	-2.70197500	2.88622100
H	2.05016400	7.34260700	-1.61461200
H	2.58609700	3.25074600	-0.40935200
H	-1.28367700	5.07088100	-0.15357200
H	-0.35766000	7.18230000	-1.02449100
H	3.51971500	5.36417100	-1.31320700
C	-7.38255900	-1.76849700	1.12854300
H	-7.80084000	-2.00730300	0.14687400
H	-7.67695100	-2.55623200	1.82571100
H	-7.84903400	-0.83639900	1.46384200
C	1.02674500	-0.43614800	-0.78187700
N	0.53407600	-0.87442600	-1.94667900
N	2.34115500	-0.72043200	-0.84324700
N	1.49929800	-1.43347400	-2.74682800
C	-0.80984400	-0.85299600	-2.46352800
C	2.58373200	-1.32675200	-2.04207900
C	3.42045400	-0.54834900	0.14024800

C	-1.47484000	-2.08402500	-2.56229400
C	-1.35585700	0.34626000	-2.92308900
C	4.00642700	-1.61959200	-2.38585500
H	3.10793500	0.25041700	0.81242200
C	4.77752500	-0.24259200	-0.59348600
C	3.67716900	-1.84019700	0.88402300
C	-2.75411800	-2.07997000	-3.10610400
C	-0.82105100	-3.37291300	-2.13659500
C	-2.64342900	0.29022900	-3.46764500
C	-0.63873900	1.66698800	-2.82680500
H	4.11742800	-1.74422300	-3.46270800
H	4.35576100	-2.52780100	-1.87593600
O	4.74204600	-0.47634300	-1.99637400
C	5.82781000	-1.13303000	0.11042000
H	5.01807000	0.81741800	-0.50714900
C	5.02741300	-2.17734700	0.84697300
C	2.74248700	-2.63695400	1.53832600
C	-3.35470600	-0.90262600	-3.56187700
H	-3.29805300	-3.01874200	-3.17557900
H	-0.22387000	-3.25354700	-1.22800900
H	-0.14668800	-3.73287700	-2.91940800
H	-1.57702600	-4.13779400	-1.95224600
H	-3.09434800	1.21223000	-3.82623000
H	-0.81100300	2.25030400	-3.73396200
H	0.43650000	1.55511300	-2.68098700
H	-1.02085300	2.24659200	-1.97859200
H	6.51888300	-1.54772700	-0.62983800
H	6.42230700	-0.53641100	0.81022800

C	5.47130300	-3.34390000	1.46444600
C	3.18997100	-3.80618100	2.14851600
H	1.70543000	-2.31563700	1.57997100
C	-4.75038900	-0.93906400	-4.12659200
C	4.54220800	-4.15715700	2.10943900
H	6.52198000	-3.61753400	1.44043600
H	2.48424300	-4.44793300	2.66591100
H	-5.45490600	-1.31354700	-3.37771600
H	-5.07908500	0.05313300	-4.44105600
H	-4.80553100	-1.60836800	-4.98989800
H	4.87283900	-5.07226100	2.59053600
H	-2.61114600	1.31526300	1.66782900
Si	-0.91256200	1.18331000	3.44002200
C	0.68799200	2.12753100	3.73098200
H	0.56376200	3.19506600	3.52856200
H	1.00815600	2.01162100	4.77215300
H	1.46239200	1.72828500	3.07303900
C	-2.28319500	2.07776500	4.39927200
H	-3.23317700	1.53757400	4.32487400
H	-2.02870400	2.16450500	5.46071700
H	-2.44422500	3.09074800	4.01445800
C	-0.77400000	-0.54291200	4.18690300
H	-1.70198400	-1.11285900	4.08354000
H	0.04365100	-1.08862700	3.71068200
H	-0.56073700	-0.44932300	5.25760600

SR-M4

Zero-point correction= 0.812937 (Hartree/Particle)

C	-0.28399100	0.06977700	0.53629800
C	-2.56867000	0.88112600	1.00756400
C	-1.30115300	2.11615300	-0.61786100
C	-2.41894400	2.06135900	0.38904500
C	-0.03051700	1.54896600	0.04692000
O	-1.76174600	-0.15630200	0.66526200
C	0.50595400	2.38958400	1.22895800
C	2.00535400	2.25143900	1.36701800
C	4.79637900	1.76459800	1.37893900
C	2.85494100	2.89938800	0.46585800
C	2.57997900	1.38271700	2.30198700
C	3.94920200	1.14468500	2.30383100
C	4.22843800	2.65381800	0.46372200
C	-3.55209000	0.54391200	2.05786700
C	-5.36394100	-0.11507100	4.08377200
C	-3.22961500	-0.43715500	3.00182000
C	-4.79437800	1.18166500	2.12932400
C	-5.69291600	0.85666600	3.14023400
C	-4.13042400	-0.76012600	4.01102000
H	-3.01541300	2.92408800	0.66818800
H	0.75732100	1.51281500	-0.71980700
H	0.24822500	3.44092600	1.06330600
H	0.01402200	2.06619700	2.14860900
H	2.43236600	3.58995000	-0.26259000
H	1.92595500	0.83612200	2.97141200
H	4.36840800	0.43358000	3.01260700
H	4.86662400	3.15968100	-0.25729300
H	-6.06811700	-0.37128600	4.86903800

H	-2.25757900	-0.91629500	2.93336300
H	-5.06064400	1.91780400	1.37652600
H	-6.65671500	1.35415100	3.18570800
H	-3.86899400	-1.51531700	4.74589600
O	0.36018100	-0.33565900	1.57219800
C	6.27673200	1.47837800	1.39239000
H	6.75041900	1.89745300	2.28572200
H	6.46250500	0.39857700	1.40217300
H	6.77470800	1.90733100	0.51878100
C	0.05834900	-0.89254300	-0.65378800
N	-0.66013500	-1.73433500	-1.40010400
N	1.33381600	-1.11591600	-1.02400900
N	0.11888300	-2.47650200	-2.25429800
C	-2.08085900	-1.96765000	-1.37543300
C	1.32689100	-2.07353300	-1.99672000
C	2.60012100	-0.58706500	-0.51548900
C	-2.60361300	-2.71137200	-0.31393800
C	-2.85470700	-1.44272100	-2.40510300
C	2.63731400	-2.36252200	-2.65394700
H	2.39791400	0.34941700	0.00351600
C	3.59611200	-0.37020000	-1.70346600
C	3.31406200	-1.54678600	0.40804600
C	-3.98107200	-2.89319000	-0.29008300
C	-1.70231300	-3.25850900	0.75942800
C	-4.23325200	-1.65730700	-2.33689800
C	-2.23013700	-0.69270500	-3.55201200
H	2.48127100	-2.83464400	-3.62357000
H	3.25428000	-3.01783700	-2.02501300

O	3.25164200	-1.10527400	-2.87531900
C	4.97263400	-0.78470600	-1.14240500
H	3.58072900	0.67466400	-2.02040600
C	4.65979500	-1.64075400	0.06008800
C	2.78949100	-2.21150900	1.51096400
C	-4.81017800	-2.36492800	-1.28519500
H	-4.42318400	-3.44910800	0.53307900
H	-1.18162700	-2.44511100	1.27761500
H	-0.94588500	-3.92483600	0.33035100
H	-2.28149800	-3.82460200	1.49110500
H	-4.86677800	-1.25172100	-3.12154500
H	-1.69151000	-1.37650400	-4.21509900
H	-1.50709500	0.05108000	-3.20116500
H	-2.99527500	-0.17758300	-4.13532100
H	5.55320300	-1.29920100	-1.91385400
H	5.53336500	0.10803000	-0.84274600
C	5.52025000	-2.42415200	0.82462000
C	3.65434000	-3.00280300	2.26522100
H	1.74376600	-2.06815000	1.77124600
C	-6.30350300	-2.54414200	-1.19679000
C	5.00601600	-3.10657500	1.92644900
H	6.57203900	-2.50619700	0.56538600
H	3.27713800	-3.53621200	3.13179600
H	-6.71516600	-1.92272400	-0.39527800
H	-6.79435600	-2.26063200	-2.12999800
H	-6.56300600	-3.58216800	-0.97236500
H	5.66398700	-3.72522700	2.52868200
Si	-1.13185300	3.73060600	-1.59084800

C	-2.75393500	3.95919100	-2.52284500
H	-2.72654800	4.85011600	-3.15747600
H	-2.96031000	3.09429100	-3.16162000
H	-3.59345600	4.06673100	-1.82899700
C	0.27754100	3.51798400	-2.82626500
H	0.10663400	2.64284100	-3.46248800
H	0.36436900	4.39247700	-3.47817700
H	1.24046000	3.38044300	-2.32375400
C	-0.82785200	5.25636300	-0.52392700
H	0.20697200	5.31895400	-0.17491300
H	-1.03720400	6.16120100	-1.10356400
H	-1.47971100	5.26528700	0.35543600
H	-1.56023900	1.38934000	-1.41417500

SS-M4

Zero-point correction= 0.814456 (Hartree/Particle)

C	0.23163800	0.60738500	-0.38730200
C	2.41130700	1.67781500	-0.65514700
C	0.98695500	2.68261900	1.01875400
C	2.09879800	2.79372000	0.01654300
C	-0.20415100	1.93802500	0.36684600
O	1.72530200	0.53024400	-0.37410400
Si	1.64760100	2.00836000	2.68648600
C	3.29071000	1.12095800	2.47534000
C	0.32470300	0.96570300	3.55164600
C	1.95779300	3.51259200	3.78526200
C	-0.91032900	2.88419500	-0.64242700
C	-2.38607600	2.61668500	-0.80111000

C	-5.13039100	1.93748800	-0.94724700
C	-3.27723300	2.99620900	0.20781900
C	-2.89476000	1.91678900	-1.89985000
C	-4.24340800	1.58615600	-1.96953500
C	-4.62644600	2.65234700	0.14253800
C	3.47017700	1.51828500	-1.66975100
C	5.45165100	1.17495600	-3.61408900
C	3.28187800	0.61143200	-2.71788000
C	4.66273300	2.24471700	-1.59939200
C	5.64554900	2.07741700	-2.56993000
C	4.26630700	0.44408700	-3.68543100
H	0.62939900	3.68667200	1.27960200
H	2.60662900	3.73056200	-0.18355900
H	-0.92460800	1.67966600	1.15640200
H	3.61204000	0.64871800	3.40921100
H	4.05241200	1.85187900	2.18585000
H	3.24621400	0.36344300	1.68889000
H	0.68217900	0.62741500	4.53006600
H	-0.56913800	1.57623000	3.72292200
H	0.01023000	0.07997400	2.99054300
H	2.65423000	4.20532300	3.30114700
H	1.03155100	4.05835900	3.99090300
H	2.39544700	3.21808800	4.74426000
H	-0.78395800	3.91097700	-0.28170400
H	-0.40773800	2.79910800	-1.60713900
H	-2.90333800	3.54639700	1.06940400
H	-2.20194500	1.57817200	-2.66278300
H	-4.61045300	1.00479400	-2.81314500

H	-5.29883300	2.94576900	0.94563100
H	6.22206900	1.03993400	-4.36671800
H	2.34393000	0.06683100	-2.76176300
H	4.82435200	2.92337400	-0.76668300
H	6.57055900	2.64175700	-2.50396200
H	4.10824000	-0.25595900	-4.50050400
O	-0.28686400	0.43022300	-1.55443800
C	-6.58403800	1.54711700	-1.03075400
H	-7.08165500	2.05494300	-1.86268400
H	-6.68552800	0.46942100	-1.20075000
H	-7.11941400	1.80407800	-0.11301300
C	-0.07894900	-0.68224600	0.46567700
N	0.67233500	-1.73785900	0.82117200
N	-1.33056900	-1.02927400	0.82861400
N	-0.06603300	-2.71996200	1.43724900
C	2.05351700	-2.04910100	0.53627700
C	-1.27609000	-2.25716800	1.42538000
C	-2.63815100	-0.46305300	0.49816100
C	2.40897000	-2.30284500	-0.79328700
C	2.94327000	-2.17740600	1.59990200
C	-2.54224100	-2.77116700	2.03241400
H	-2.51532100	0.59000800	0.25424200
C	-3.62169700	-0.65443500	1.69857500
C	-3.29365300	-1.20250400	-0.64656100
C	3.74789400	-2.57563300	-1.04984600
C	1.37821300	-2.32143500	-1.88819600
C	4.27472300	-2.46344700	1.28808600
C	2.49581300	-2.04361600	3.03048600

H	-2.31629400	-3.48068600	2.82828800
H	-3.16797400	-3.26311100	1.27623500
O	-3.18957100	-1.65491400	2.61562200
C	-4.97494000	-1.00765200	1.04451800
H	-3.67813600	0.25881400	2.29402800
C	-4.61876400	-1.49764100	-0.33753600
C	-2.73368400	-1.51868500	-1.87924500
C	4.69811500	-2.63581400	-0.02693800
H	4.05851800	-2.74565600	-2.07796400
H	0.87246400	-1.35389000	-1.99025900
H	0.61026400	-3.07403000	-1.67101700
H	1.84547600	-2.58275900	-2.84019900
H	4.99464100	-2.54339900	2.09861900
H	1.93244600	-2.92893600	3.33955000
H	1.84574700	-1.17654300	3.16842100
H	3.35893400	-1.93314800	3.68970600
H	-5.50688700	-1.74413800	1.65402300
H	-5.60119500	-0.10998700	0.98197600
C	-5.42398300	-2.13151300	-1.28010200
C	-3.54303800	-2.16031800	-2.81523600
H	-1.70866900	-1.22365900	-2.09436300
C	6.14738400	-2.88308000	-0.35454600
C	-4.87517600	-2.46153400	-2.51893500
H	-6.45972100	-2.36919400	-1.05403700
H	-3.13858200	-2.42025700	-3.78813600
H	6.57922600	-2.00930300	-0.85257900
H	6.73114300	-3.08230600	0.54641700
H	6.25836500	-3.73402700	-1.03218500

H	-5.49021400	-2.95995800	-3.26158700

RR-TS5

Zero-point correction= 0.811918 (Hartree/Particle)

C	0.65139900	-0.43484600	-1.06095100
C	-0.51982000	-2.54206900	-0.19656500
C	1.90831800	-2.11831500	-0.07111400
C	0.90899600	-2.99784000	-0.19714400
O	1.61178200	-0.77077600	-0.03309300
C	-0.71766700	-1.09255200	-0.75433700
O	1.06448900	-0.32926700	-2.20909000
Si	-1.15231700	-2.72488200	1.59285400
C	-0.88613500	-4.50469400	2.15093900
C	-0.13750700	-1.54897300	2.66497600
C	-2.98627000	-2.31537600	1.70282600
C	3.34593700	-2.43620800	-0.04049500
C	6.07260700	-3.05044500	-0.11247000
C	4.24846900	-1.56210500	-0.65195200
C	3.82167800	-3.61007600	0.55451200
C	5.17759900	-3.91619100	0.51554300
C	5.60303500	-1.87436800	-0.69441200
H	-1.11056600	-3.23555100	-0.80671900
H	1.13172700	-4.05573500	-0.28852900
H	-1.42306500	-5.20521000	1.50356600
H	-1.24836000	-4.65050500	3.17321100
H	0.17567400	-4.76795100	2.12995600
H	0.08149500	-0.63071200	2.10971700
H	0.82729700	-1.99872800	2.91992600

H	-0.65279900	-1.28771300	3.59460200
H	-3.56319900	-2.90815700	0.98404100
H	-3.35950100	-2.53541000	2.70910500
H	-3.18952300	-1.26105600	1.49352300
H	7.13077700	-3.29083600	-0.14351600
H	3.86532800	-0.65215100	-1.10376900
H	3.12403100	-4.27170200	1.06083000
H	5.53901100	-4.82675500	0.98315300
H	6.29186600	-1.19566100	-1.18950600
C	-1.56863200	-1.11040000	-2.03757100
H	-1.22919200	-0.48093800	0.00205200
C	-2.96919500	-1.63602000	-1.82408000
C	-3.31715600	-2.92879900	-2.22291500
C	-3.95699400	-0.86763400	-1.19798800
C	-4.59352200	-3.43884100	-1.99446600
H	-2.57529700	-3.54619500	-2.72367100
C	-5.23305800	-1.37150100	-0.97614100
H	-3.73041500	0.13707200	-0.84868900
C	-5.57491500	-2.66832700	-1.36981300
H	-4.83137100	-4.45081500	-2.31208000
H	-5.97413300	-0.75106900	-0.47802500
H	-1.59621700	-0.10383900	-2.47228700
H	-1.04069900	-1.72625400	-2.77148200
C	0.49545000	1.44988800	-0.33459900
N	1.56174300	2.21790100	-0.05984400
N	-0.51380600	2.36026700	-0.30712400
N	1.27022200	3.55189800	0.14368800
C	2.95795400	1.86732600	-0.04517600

C	-0.01538100	3.59861100	-0.00464200
C	-1.93957900	2.19330300	-0.51026800
C	3.54225700	1.46380500	1.15673600
C	3.68414300	2.04553100	-1.22188600
C	-0.97536500	4.74161900	-0.01026800
H	-2.07575800	1.29431400	-1.11207400
C	-2.56013500	3.41969800	-1.26342300
C	-2.72164900	2.11883500	0.78204000
C	4.91749800	1.25194400	1.16195000
C	2.70069500	1.23014300	2.37916700
C	5.06239000	1.82223800	-1.16679500
C	2.98927100	2.43195700	-2.49920400
H	-0.44236700	5.68856400	-0.09148900
H	-1.58160200	4.74620700	0.90588800
O	-1.77884700	4.60299500	-1.17067500
C	-3.96184900	3.59083500	-0.63504800
H	-2.62017400	3.21493500	-2.33361300
C	-3.87479200	2.89687100	0.70257500
C	-2.40842200	1.39662100	1.92730900
C	5.69338800	1.43866100	0.01538000
H	5.39626700	0.92811300	2.08310200
H	2.09830600	0.32726100	2.23628100
H	2.01740000	2.06547100	2.55948000
H	3.32761800	1.09649100	3.26247900
H	5.65148500	1.95414100	-2.07115800
H	2.43435800	3.36761600	-2.37753300
H	2.27966800	1.64617700	-2.78065200
H	3.71230600	2.56214000	-3.30627100

H	-4.21310700	4.65249800	-0.56287200
H	-4.71853300	3.11549400	-1.26966100
C	-4.75500900	2.94424100	1.77940200
C	-3.29196700	1.44413800	3.00403600
H	-1.49184700	0.81555800	1.98645400
C	7.18350200	1.22001800	0.07043200
C	-4.45713800	2.20984300	2.92678400
H	-5.65493200	3.54995000	1.73200100
H	-3.07013100	0.88446300	3.90686900
H	7.41660000	0.26362500	0.54784800
H	7.62231500	1.22053800	-0.93015600
H	7.67232600	2.00728900	0.65287400
H	-5.13433500	2.24095700	3.77413400
C	-6.96675700	-3.20282700	-1.14876200
H	-7.64615700	-2.86321100	-1.93744100
H	-6.97594800	-4.29513700	-1.15212600
H	-7.37473200	-2.85962200	-0.19463200

RS-TS5

Zero-point correction= 0.812608 (Hartree/Particle)

C	0.37502900	-0.98601400	-0.56545200
C	-1.11913900	-2.18733000	0.98110900
C	0.94308100	-1.09335500	1.78951100
C	-0.16179500	-1.80053400	2.07079000
O	1.28660400	-0.69028800	0.52125100
C	-1.06374900	-1.06049600	-0.06743100
O	0.78712000	-1.74959800	-1.43620900
C	1.95521100	-0.67564800	2.78284300
C	3.89457500	0.09113800	4.65135900

C	3.30539400	-0.61716400	2.41824500
C	1.58600900	-0.33324600	4.08865800
C	2.55111400	0.04296200	5.01812100
C	4.26691900	-0.23829000	3.34929900
H	-0.31528500	-2.13694600	3.09076700
H	4.64626600	0.38945000	5.37504600
H	3.58590400	-0.87092900	1.39967000
H	0.53522800	-0.34473000	4.36397900
H	2.25242500	0.30937200	6.02711900
H	5.31235200	-0.20223200	3.05745900
C	-2.01580600	-1.19943600	-1.24900200
H	-1.27075000	-0.13148300	0.47910800
C	-3.48440100	-1.24640600	-0.88840700
C	-4.36771800	-1.99219700	-1.67583800
C	-4.01994300	-0.53641500	0.19080500
C	-5.73058000	-2.02100100	-1.40307900
H	-3.97549800	-2.55423200	-2.52018200
C	-5.38606000	-0.56582300	0.46312400
H	-3.36860900	0.04112700	0.84378700
C	-6.26480300	-1.30803100	-0.32634600
H	-6.39233400	-2.60883200	-2.03434200
H	-5.77458300	-0.00514500	1.30952200
H	-1.83555800	-0.35730100	-1.93556700
H	-1.74634600	-2.08909000	-1.82073400
C	0.47781700	0.87099700	-1.27800000
N	1.61064200	1.27958800	-1.86235700
N	-0.28087300	1.99883500	-1.30180300
N	1.61336700	2.60468000	-2.23785100

C	2.83043500	0.52924300	-1.98562900
C	0.44335600	3.01781700	-1.86464900
C	-1.53813000	2.28730600	-0.62250400
C	3.80977700	0.73806900	-1.01287900
C	2.99108100	-0.34435900	-3.05889600
C	-0.21382600	4.35365900	-1.98264100
H	-2.14574100	1.38050900	-0.63028700
C	-2.32546700	3.44894200	-1.32934200
C	-1.28173700	2.79434500	0.77947900
C	4.98995800	0.00528900	-1.12481900
C	3.58698800	1.72783000	0.09862100
C	4.19121200	-1.05005300	-3.13111800
C	1.89961000	-0.52014200	-4.07731900
H	0.23841200	4.93575200	-2.78541800
H	-0.11819000	4.91103400	-1.03925000
O	-1.56972900	4.12825800	-2.31877900
C	-2.79433100	4.38062000	-0.18315900
H	-3.17292800	3.03770600	-1.87869000
C	-1.99192700	3.96584700	1.02525700
C	-0.42722100	2.24342600	1.73098800
C	5.19238200	-0.89652700	-2.17156000
H	5.76931800	0.14553400	-0.37879800
H	2.65981000	1.49706600	0.63427900
H	3.50649000	2.74465100	-0.29765200
H	4.40857100	1.69566300	0.81678800
H	4.34406100	-1.74147300	-3.95609000
H	1.56279200	0.44664600	-4.46372300
H	1.04854200	-1.02175700	-3.60682600

H	2.24908200	-1.12585700	-4.91540600
H	-2.63631100	5.42605300	-0.46630600
H	-3.86749500	4.25355700	-0.00752800
C	-1.88009800	4.59648100	2.26198000
C	-0.31528700	2.87860200	2.96540500
H	0.15959600	1.35445300	1.50789300
C	6.46052600	-1.70706400	-2.25065500
C	-1.04310100	4.04203000	3.22875900
H	-2.42525500	5.51310700	2.46656600
H	0.35060300	2.46896400	3.71853600
H	7.30214700	-1.17135600	-1.80531400
H	6.34647700	-2.65284000	-1.71110200
H	6.71293800	-1.94655700	-3.28621400
H	-0.94308500	4.52925000	4.19336300
C	-7.73798300	-1.36797600	-0.01335600
H	-7.98475300	-2.28437900	0.53260500
H	-8.04621600	-0.52140800	0.60440100
H	-8.33704700	-1.36101600	-0.92759300
Si	-0.68443400	-3.99636300	0.47243300
H	-2.14010600	-2.23496100	1.38236700
C	1.17212300	-4.28497600	0.51909800
H	1.68188200	-3.56029700	-0.12118400
H	1.55993600	-4.18641300	1.53707200
H	1.40360000	-5.29354600	0.16062800
C	-1.50144100	-5.05885700	1.80497800
H	-2.59152100	-4.96249100	1.77569000
H	-1.25383700	-6.11581700	1.66487900
H	-1.16408000	-4.76998200	2.80575400

C	-1.37056200	-4.52765900	-1.20091100
H	-0.81272700	-4.04664700	-2.00896700
H	-1.26247800	-5.61238200	-1.30735300
H	-2.43247800	-4.28304400	-1.30544400

SR-TS5

Zero-point correction= 0.812137 (Hartree/Particle)

C	-0.03726200	0.46190700	-1.01540400
C	2.33700900	0.90416500	-0.92154600
C	0.88976500	2.26832000	0.59504700
C	2.21007500	1.78529000	0.07521200
C	-0.31632900	1.76180700	-0.23155200
O	1.25561600	0.34338100	-1.54743700
C	-1.02976000	2.73777800	-1.21383100
C	-2.50609300	2.39784500	-1.27807000
C	-5.17785800	1.46461500	-1.16237600
C	-3.32752400	2.69475300	-0.18758600
C	-3.05758000	1.66508000	-2.33229700
C	-4.36622400	1.20312100	-2.27155500
C	-4.64291600	2.23408800	-0.12794200
C	3.61112100	0.41597400	-1.49756000
C	5.99702700	-0.57513100	-2.59906900
C	3.59130300	-0.37184900	-2.65489500
C	4.84654900	0.70329000	-0.90385500
C	6.02632100	0.21659000	-1.45174500
C	4.77437400	-0.86457200	-3.19770300
H	3.11435700	2.17138900	0.53369800
H	-1.08007800	1.50772900	0.50647600

H	-0.92120300	3.76448700	-0.85536800
H	-0.57055900	2.68992600	-2.20643900
H	-2.92380200	3.26628800	0.64642700
H	-2.42892900	1.39925500	-3.17509000
H	-4.76094100	0.60355800	-3.08811400
H	-5.25851700	2.47105600	0.73719100
H	6.92033400	-0.95902700	-3.02168600
H	2.63991800	-0.59995300	-3.12097300
H	4.89203100	1.28940600	0.00797000
H	6.97385200	0.44539000	-0.97334000
H	4.73823500	-1.47602000	-4.09408500
O	-0.90639900	-0.05888000	-1.71282400
C	-6.57971400	0.91432800	-1.09649700
H	-7.16533700	1.21693700	-1.96947600
H	-6.56254400	-0.18171800	-1.07971200
H	-7.10243600	1.26194900	-0.20160700
C	0.04056600	-0.79765900	0.58734600
N	0.85054600	-1.61094600	1.27517000
N	-1.18773600	-1.15986300	1.04904800
N	0.20569900	-2.45142900	2.16270800
C	2.26092000	-1.78093300	1.06800700
C	-1.04262200	-2.14034000	1.99424000
C	-2.53554000	-0.76057000	0.64246100
C	2.67617200	-2.49691200	-0.05610100
C	3.15355600	-1.25534600	2.00425300
C	-2.27366200	-2.55229600	2.73397800
H	-2.48081800	0.18048600	0.09544700
C	-3.45920900	-0.62924200	1.89711600

C	-3.23150300	-1.79121500	-0.22070800
C	4.04590300	-2.71427200	-0.21125100
C	1.67810000	-2.99031200	-1.06999800
C	4.51050300	-1.49393500	1.80409900
C	2.65485000	-0.43471100	3.16245000
H	-2.01003900	-3.01948100	3.68274500
H	-2.87539200	-3.25362800	2.14109300
O	-2.98672700	-1.36058600	3.02547000
C	-4.83912300	-1.13141000	1.42392200
H	-3.49723400	0.40909700	2.23499700
C	-4.54000700	-1.98703800	0.21739000
C	-2.72786700	-2.45695100	-1.33193400
C	4.97198600	-2.23118800	0.71038100
H	4.39562600	-3.25897000	-1.08465800
H	1.20109700	-2.14509000	-1.58003600
H	0.88802200	-3.57935400	-0.59214800
H	2.17080300	-3.61432400	-1.81800800
H	5.22612400	-1.09349700	2.51872000
H	1.87298200	-0.96181100	3.71554600
H	2.22612800	0.50679800	2.80005200
H	3.46999800	-0.19727700	3.84831000
H	-5.34357000	-1.66753400	2.23300600
H	-5.46841700	-0.27856900	1.14350200
C	-5.37747600	-2.86895800	-0.45986800
C	-3.56698300	-3.34579700	-2.00194200
H	-1.71682900	-2.25392700	-1.67135200
C	6.44332700	-2.50568700	0.53945700
C	-4.87977400	-3.54900400	-1.57067700

H	-6.39785100	-3.03137900	-0.12396100
H	-3.19944600	-3.88062300	-2.87175100
H	7.04402900	-1.66868000	0.90501500
H	6.73995600	-3.39751500	1.10160300
H	6.69036000	-2.66917400	-0.51193100
H	-5.51881300	-4.24498800	-2.10500600
Si	1.01699000	4.13539900	0.98476300
C	2.33560600	4.28864700	2.32602700
H	2.39562700	5.32264900	2.68000100
H	2.10008400	3.65557400	3.18778100
H	3.32931300	4.00910500	1.96406200
C	-0.60426100	4.78725800	1.70105600
H	-1.04649600	4.05784800	2.38852400
H	-0.41850600	5.70403200	2.26951900
H	-1.34623100	5.02223200	0.93314100
C	1.56518500	5.13332500	-0.51425000
H	0.80103600	5.18094500	-1.29417000
H	1.81003300	6.15845000	-0.21908500
H	2.46262300	4.68670100	-0.95364900
H	0.76849500	1.84290600	1.60660200

SS-TS5

Zero-point correction= 0.812805 (Hartree/Particle)

C	-0.26714500	-0.93761400	-0.39369300
C	-2.46271200	-1.83684400	-0.41126400
C	-1.08664700	-2.51087600	1.46699500
C	-2.19383500	-2.78782200	0.49193100
C	0.14854900	-1.94451300	0.72173200

O	-1.69343700	-0.69461700	-0.40175100
Si	-1.76719100	-1.47768700	2.93724700
C	-3.29694700	-0.50228100	2.45248000
C	-0.39766700	-0.39728100	3.66227800
C	-2.27384000	-2.72636200	4.25976100
C	0.96617600	-3.09905800	0.08087200
C	2.42889900	-2.78142400	-0.11493700
C	5.15797200	-2.07906200	-0.38814800
C	3.30072100	-2.83666400	0.97718100
C	2.94635300	-2.37843800	-1.34997800
C	4.28721500	-2.03523300	-1.48107500
C	4.64207900	-2.48156600	0.84625500
C	-3.53089600	-1.83059700	-1.42579100
C	-5.53884500	-1.78182900	-3.37294000
C	-3.33363600	-1.14191400	-2.62749000
C	-4.74648100	-2.48596100	-1.20449300
C	-5.74179000	-2.46626400	-2.17586800
C	-4.33184800	-1.12084000	-3.59531700
H	-0.77317200	-3.44899200	1.94074200
H	-2.75959700	-3.71257100	0.51364100
H	0.79773400	-1.43130100	1.44109600
H	-3.59936800	0.18343600	3.25097500
H	-4.12552400	-1.19331100	2.26752400
H	-3.13640100	0.07301300	1.53744000
H	-0.75612900	0.13524400	4.54949200
H	0.45023500	-1.01880900	3.97147900
H	-0.02108700	0.34583500	2.95220500
H	-3.00168900	-3.43939400	3.85822700

H	-1.41385500	-3.29569000	4.62621400
H	-2.73696800	-2.22710800	5.11653700
H	0.89485700	-3.96425700	0.74871300
H	0.50418300	-3.37704500	-0.86940600
H	2.92008700	-3.15009500	1.94752500
H	2.27324700	-2.29196700	-2.19611500
H	4.66134900	-1.69306700	-2.44392000
H	5.30024100	-2.52813400	1.71097900
H	-6.31963400	-1.76035700	-4.12651900
H	-2.38251800	-0.64430700	-2.78733900
H	-4.91630800	-2.98989000	-0.25735100
H	-6.68392200	-2.97327800	-1.99270900
H	-4.16729800	-0.58960500	-4.52798600
O	0.28808900	-0.96182500	-1.49491000
C	6.61026700	-1.71141500	-0.55477800
H	7.13149800	-2.44608000	-1.17639800
H	6.70963500	-0.73861800	-1.04831100
H	7.12394700	-1.66561200	0.40909600
C	0.11751700	0.88425400	0.36561700
N	-0.58734500	2.02347500	0.49753800
N	1.38405900	1.30269900	0.60771300
N	0.17460100	3.12182200	0.83921900
C	-1.97852800	2.24656800	0.20786200
C	1.37604500	2.64167500	0.89553000
C	2.66165100	0.62642500	0.41263200
C	-2.40464600	2.14596700	-1.12255300
C	-2.83205300	2.60941200	1.24814000
C	2.67087000	3.25225900	1.32348400

H	2.49928400	-0.45061200	0.46723600
C	3.69813000	1.09483700	1.49095400
C	3.30002100	1.00193800	-0.90653600
C	-3.75847100	2.33603800	-1.37737200
C	-1.42227800	1.85720500	-2.22577200
C	-4.17914400	2.80844700	0.93811300
C	-2.32422300	2.76814500	2.65564300
H	2.49201300	4.14473700	1.92301700
H	3.28770700	3.51939300	0.45472200
O	3.31143700	2.29518300	2.15002500
C	5.03046400	1.25683800	0.72573900
H	3.77409300	0.35492500	2.28990600
C	4.63685500	1.34635100	-0.72753600
C	2.70953900	0.97963600	-2.16509600
C	-4.66126900	2.65536100	-0.35939800
H	-4.11955000	2.23072100	-2.39782600
H	-0.86906800	0.94577900	-1.98944300
H	-0.70243500	2.67820000	-2.32285500
H	-1.93714400	1.73420400	-3.18046500
H	-4.86663900	3.07251800	1.73785500
H	-1.68555400	3.65161900	2.74210400
H	-1.72548600	1.90290200	2.95550000
H	-3.15762600	2.86891300	3.35376400
H	5.56819300	2.13676700	1.09193600
H	5.66860400	0.38285600	0.89821200
C	5.42112500	1.67906200	-1.82911200
C	3.49748300	1.31719800	-3.26391500
H	1.67379900	0.66750100	-2.27681700

C	-6.12585700	2.81981100	-0.67251200
C	4.84155100	1.66164100	-3.09711300
H	6.46520600	1.95236100	-1.70341600
H	3.06620000	1.30587700	-4.25967100
H	-6.56242300	1.86306700	-0.97552500
H	-6.67942600	3.18608500	0.19445900
H	-6.27514700	3.52272700	-1.49694300
H	5.44027600	1.92186000	-3.96431900

RR-P

Zero-point correction= 0.426529 (Hartree/Particle)

C	-1.71710100	0.49710500	-0.97760700
C	-0.78663300	1.66574900	-0.89104100
C	0.57390500	1.18404500	-0.36084000
C	0.36652500	0.37204800	0.90024100
C	-1.59363700	-0.54687400	-0.15016600
H	-2.53072700	0.50315600	-1.69372800
C	-2.43550400	-1.75765800	-0.11532600
C	-3.18279700	-2.14254600	-1.23554200
C	-2.50079000	-2.53772800	1.04479500
C	-3.99688700	-3.26696100	-1.18694500
H	-3.10863500	-1.57262500	-2.15626400
C	-3.31359700	-3.66637200	1.08694700
H	-1.91540300	-2.25104500	1.91071800
C	-4.06708700	-4.03304500	-0.02444400
H	-4.56749500	-3.55461500	-2.06400000
H	-3.35791600	-4.26014700	1.99419400
H	-4.69876200	-4.91458300	0.01016100

O	1.02795700	0.45157300	1.89812800
O	-0.63095700	-0.56111000	0.85485500
H	-0.63865200	2.09704200	-1.89054300
Si	-1.58681900	3.04772400	0.17306700
C	-3.22224000	3.50428300	-0.63557300
H	-3.08056300	3.81335000	-1.67579900
H	-3.91457300	2.65726900	-0.62321500
H	-3.69872100	4.33245300	-0.10252900
C	-0.42885100	4.53101100	0.19623500
H	0.51541500	4.29614500	0.69636400
H	-0.20146900	4.87236000	-0.81831900
H	-0.88816400	5.36554500	0.73479500
C	-1.87941500	2.40749300	1.91618800
H	-2.45561900	3.14031900	2.48955000
H	-2.44771900	1.47185800	1.90213400
H	-0.94185100	2.22649600	2.44952000
H	1.21778400	2.02055500	-0.07273100
C	1.32979400	0.32179500	-1.40426500
H	1.47755600	0.95045600	-2.28914000
H	0.68744600	-0.51259800	-1.70527400
C	2.65608800	-0.19405300	-0.90721200
C	3.82172700	0.55619000	-1.06598900
C	2.74155200	-1.41768800	-0.23832400
C	5.03929500	0.09513700	-0.57467800
H	3.77531500	1.51273400	-1.58151000
C	3.95828800	-1.87627600	0.25321400
H	1.84173200	-2.01269600	-0.09610900
C	5.12797900	-1.12934400	0.09017000

H	5.93587800	0.69455100	-0.70965900
H	4.00268900	-2.82944300	0.77362400
C	6.45023000	-1.64666100	0.59624000
H	6.89332600	-2.35095700	-0.11562400
H	7.16324400	-0.83230300	0.74364600
H	6.33027800	-2.17301600	1.54646600

***RS*-P**

Zero-point correction= 0.426771 (Hartree/Particle)

C	-0.14332500	-0.23223600	0.90734400
C	0.50004900	1.32892600	-0.93111300
C	1.98702200	-0.48724400	-0.16953000
C	1.73806700	0.49378900	-1.04372000
O	1.11924400	-0.74611000	0.88698500
C	-0.61886700	0.42898500	-0.37223900
O	-0.78394900	-0.34519000	1.91739000
Si	0.91986700	2.91937200	0.06545700
C	1.15282800	2.48106500	1.87879600
C	-0.42000600	4.22777600	-0.14018900
C	2.52870300	3.58453700	-0.65082200
C	3.15400600	-1.38936500	-0.15315200
C	5.34685200	-3.13259200	-0.15754300
C	3.08608100	-2.61629800	0.51592100
C	4.34177000	-1.03684200	-0.80616700
C	5.42608800	-1.90496400	-0.81324800
C	4.17592700	-3.48137100	0.50932400
H	0.19367200	1.66354000	-1.93043000
H	2.44381400	0.67382200	-1.84617300

H	1.51447100	3.35587800	2.42810300
H	1.88881800	1.67987400	2.00162900
H	0.22023900	2.15181000	2.34654700
H	-0.65207200	4.39270800	-1.19715800
H	-1.34741000	3.96128100	0.37253900
H	-0.07093400	5.17910700	0.27423500
H	2.79006100	4.53976300	-0.18559300
H	2.44599700	3.75097500	-1.72976700
H	3.35410500	2.88794600	-0.47649900
H	6.19667000	-3.80721000	-0.15897000
H	2.17632200	-2.88622900	1.03988400
H	4.42567800	-0.06960900	-1.29189500
H	6.34096200	-1.61661300	-1.32063200
H	4.10816600	-4.43145700	1.02925900
C	-1.96936900	1.12621400	-0.16084600
H	-2.09187000	1.86544500	-0.96134600
H	-1.94814500	1.67240600	0.78830300
C	-3.14326100	0.17377800	-0.18135900
C	-3.73521600	-0.29062300	0.99435900
C	-3.65159000	-0.27902300	-1.40115500
C	-4.80524900	-1.17866100	0.94715900
H	-3.33617000	0.03333400	1.95011800
C	-4.71879600	-1.17032600	-1.44595200
H	-3.20577600	0.07273500	-2.32942700
C	-5.31655000	-1.63184100	-0.27122300
H	-5.25022200	-1.53054100	1.87450200
H	-5.09708800	-1.51012900	-2.40670600
H	-0.75532300	-0.39731600	-1.08759800

C	-6.49562200	-2.57010900	-0.31467000
H	-7.43847000	-2.01649900	-0.25346500
H	-6.51023900	-3.14530900	-1.24343300
H	-6.47300400	-3.27196800	0.52272400

SR-P

Zero-point correction= 0.427577 (Hartree/Particle)

C	1.60984400	0.62982700	1.08898400
C	0.11841700	0.47422600	1.18909800
C	-0.13080600	-1.04996900	1.16720000
C	0.45243700	-1.64201500	-0.09779800
C	2.31948800	-0.18459100	0.30027900
H	2.12102200	1.41213500	1.63736300
C	3.77413800	-0.15161500	0.06073800
C	4.51563500	1.00821000	0.31906400
C	4.43467900	-1.28424700	-0.42773600
C	5.89026800	1.02420400	0.12013200
H	4.01219900	1.90860100	0.65736200
C	5.81118300	-1.26241400	-0.62959900
H	3.86289800	-2.17825100	-0.64884100
C	6.54457500	-0.11223800	-0.35274400
H	6.45105600	1.93103400	0.32182900
H	6.31105600	-2.14901200	-1.00596700
H	7.61753200	-0.09634400	-0.51323500
O	-0.04321800	-2.49641700	-0.78363400
O	1.69436300	-1.19052600	-0.43355500
H	0.53677200	-1.45635500	1.94574500
C	-1.52114600	-1.59664400	1.51844500

H	-1.66150000	-1.42100500	2.59004800
H	-1.47441600	-2.68295800	1.38405600
C	-2.73972800	-1.06783400	0.79761800
C	-3.73727000	-0.40807200	1.51903900
C	-2.96310400	-1.30638900	-0.56396800
C	-4.91538900	0.00963700	0.90636400
H	-3.59485100	-0.22869000	2.58208400
C	-4.13488700	-0.87423900	-1.17708100
H	-2.22305700	-1.85928400	-1.13354600
C	-5.13112400	-0.20914200	-0.45495000
H	-5.67740600	0.51550500	1.49371700
H	-4.28812200	-1.07247600	-2.23500500
H	-0.23175200	0.86554800	2.15233700
Si	-0.56697800	1.65929800	-0.18348000
C	0.50175400	3.21295800	-0.05322000
H	0.50592300	3.61381900	0.96627400
H	1.53728000	3.02282900	-0.34857900
H	0.10147400	3.99141400	-0.71041400
C	-2.34589600	2.21864800	0.04963800
H	-3.05990300	1.49977700	-0.35580300
H	-2.59059200	2.38106100	1.10337100
H	-2.47901600	3.17047000	-0.47602500
C	-0.31790400	0.89682600	-1.88470100
H	-0.63120100	1.61870100	-2.64611600
H	0.73225700	0.65040300	-2.06698500
H	-0.91172000	-0.01098300	-2.02147000
C	-6.38612200	0.27690000	-1.13307700
H	-6.68058800	-0.39083100	-1.94633600

H	-7.21676800	0.34557300	-0.42677900
H	-6.23430200	1.27251700	-1.56319700

***SS*-P**

Zero-point correction= 0.426759 (Hartree/Particle)

C	1.36575200	0.53070200	-0.49632800
C	-0.06773600	0.70947600	-0.09099800
C	-0.29030200	0.04854900	1.28447500
C	0.39814000	-1.30173800	1.39429700
C	2.15599700	-0.40349400	0.04046400
H	1.79488400	1.17025000	-1.25952100
H	-0.72485200	0.23929600	-0.83912100
Si	-0.50295800	2.57914500	-0.09167700
C	0.29684200	3.36201200	-1.60804400
H	-0.08563500	4.37793400	-1.74580100
H	1.38435600	3.42996500	-1.51163700
H	0.07098800	2.79697300	-2.51811800
C	-2.35897500	2.87319800	-0.19269400
H	-2.85894400	2.70281200	0.76399700
H	-2.54591200	3.91199300	-0.48356900
H	-2.83045200	2.22189000	-0.93450800
C	0.20542500	3.35332600	1.47136100
H	0.11716500	4.44318100	1.43098900
H	-0.31851400	3.01298500	2.37031900
H	1.26640900	3.10703600	1.58406800
C	3.59069100	-0.60555200	-0.24007800
C	4.37990000	0.44958000	-0.71426300
C	4.18055900	-1.85863900	-0.03987800

C	5.72214100	0.24752300	-1.01028900
H	3.94576500	1.43796800	-0.82862000
C	5.52695000	-2.05461500	-0.33071400
H	3.57735100	-2.67531000	0.33967400
C	6.30078300	-1.00627500	-0.82098700
H	6.32215700	1.07498400	-1.37476500
H	5.97167900	-3.03204000	-0.17439000
H	7.35098900	-1.16123500	-1.04549100
O	-0.05219600	-2.27077800	1.94568300
O	1.66107800	-1.37697300	0.89755600
H	0.25880800	0.65595700	2.02242600
C	-1.74976000	-0.03514100	1.75574200
H	-2.06456800	0.96554100	2.07143000
H	-1.76988400	-0.66469300	2.65152400
C	-2.76064000	-0.56122700	0.75625500
C	-4.03133600	0.01350500	0.69510100
C	-2.49478300	-1.63975200	-0.09570400
C	-4.99957700	-0.45851700	-0.18750700
H	-4.27004700	0.84827700	1.34950000
C	-3.45970000	-2.10555800	-0.98153000
H	-1.52916200	-2.13346100	-0.06476200
C	-4.72963700	-1.52510800	-1.04420000
H	-5.97827100	0.01347800	-0.21272000
H	-3.22358100	-2.94132300	-1.63551300
C	-5.77327900	-2.05463800	-1.99391600
H	-5.35395400	-2.21895800	-2.99025800
H	-6.16885500	-3.01369100	-1.64445900
H	-6.61228300	-1.36151600	-2.08650600

R1H

Zero-point correction= 0.144874 (Hartree/Particle)

C	-3.20179100	0.05976500	0.00000600
C	-2.58104400	-1.18606700	0.00000100
C	-1.19275300	-1.26847800	-0.00000400
C	-0.40395300	-0.11065100	-0.00000500
C	-1.04303000	1.13858800	0.00000000
C	-2.42821400	1.22145400	0.00000500
H	-4.28466300	0.12873000	0.00001000
H	-3.17683300	-2.09268800	0.00000100
H	-0.70566300	-2.23955800	-0.00000800
H	-0.45347400	2.04975700	0.00000000
H	-2.90966100	2.19378200	0.00000900
C	1.05330500	-0.25583700	-0.00001000
H	1.43422200	-1.27704700	-0.00001500
C	1.97330600	0.72173100	-0.00000900
H	1.71252100	1.77576900	-0.00000400
C	3.41002700	0.38271600	-0.00001400
O	3.84566200	-0.75055800	0.00001800
H	4.10313700	1.24639800	0.00003900

R1Cl

Zero-point correction= 0.135210 (Hartree/Particle)

C	-0.24891500	1.30338000	0.00005600

C	-1.52334200	-1.17150500	-0.00029000
C	-1.63757900	1.25215000	0.00011900
C	0.52251600	0.13452400	-0.00017900
C	-0.13873000	-1.10282100	-0.00034100
C	-2.26167100	0.01051100	-0.00010600
C	1.98136000	0.25563700	-0.00018800
C	2.88330600	-0.73788600	0.00042300
C	4.32651200	-0.42143100	0.00032700
O	4.77700700	0.70548400	-0.00029500
H	5.00650700	-1.29493000	0.00088200
H	0.24863200	2.26865400	0.00020100
H	-2.03367900	-2.12773400	-0.00039800
H	-2.23055000	2.15932800	0.00035000
H	0.43128700	-2.02588400	-0.00057300
H	2.37904900	1.27020500	-0.00065100
H	2.60548200	-1.78762700	0.00105000
Cl	-4.00256000	-0.07301400	0.00015100

Si-TS1H

Zero-point correction= 0.530798 (Hartree/Particle)

C	-3.87674400	-3.84395900	0.61762600
C	-2.72751500	-3.37624200	-0.00892400
C	-2.79580700	-2.33016000	-0.93882200
C	-4.05035400	-1.78736900	-1.24104200
C	-5.20041300	-2.24993400	-0.61052700
C	-5.11747100	-3.27817700	0.32493400
H	-3.80662100	-4.65731300	1.33336500
H	-1.76699900	-3.83105900	0.21549000

H	-4.11331300	-0.97185500	-1.95690900
H	-6.16161000	-1.80411700	-0.84643400
C	-1.59776900	-1.72436600	-1.53848200
H	-1.73746300	-1.13188300	-2.44240500
C	-0.37188300	-1.73672400	-1.01055000
C	0.75000000	-0.94324900	-1.62090200
H	-0.14063500	-2.27338700	-0.09096300
O	0.55392800	-0.22109600	-2.62916400
H	1.73661500	-1.43530900	-1.48065600
C	-2.18769900	-0.00307300	1.19350200
C	-4.43107300	1.67815100	0.95061700
C	-3.35397000	-0.27170600	1.90861500
C	-2.16525500	1.09830300	0.35048200
C	-3.26725200	1.94786300	0.24196000
C	-4.46910100	0.55545500	1.77872100
C	-2.95897400	3.09555200	-0.69478700
C	-1.42972400	3.06749400	-0.77552000
C	-1.08178300	1.57361600	-0.59336900
N	0.28993500	1.44158500	-0.11766800
C	1.04881500	2.49124100	0.32566300
C	0.46408800	3.87113800	0.38670800
O	-0.94239000	3.80527500	0.33925900
N	2.26914300	2.11451500	0.56805100
N	2.24795700	0.77495400	0.24666700
C	1.06489300	0.33089600	-0.18479000
C	3.45353600	0.00139800	0.30141800
C	5.77286300	-1.50592900	0.38201800
C	4.25060900	-0.05180300	-0.84559700

C	3.77223500	-0.66473400	1.48461700
C	4.94657000	-1.41584400	1.50316000
C	5.41281300	-0.81823600	-0.77897300
C	3.85939900	0.70425200	-2.08838200
C	2.86915300	-0.56489600	2.68477800
C	7.01984000	-2.35176100	0.41231000
H	-1.31981500	-0.64992600	1.28140600
H	-5.29294300	2.33421400	0.87093500
H	-3.39936100	-1.14216200	2.55502500
H	-5.37302100	0.32796500	2.33489300
H	-3.38947300	2.92741200	-1.68850000
H	-3.30658400	4.06563100	-0.33158300
H	-1.02189700	3.48166800	-1.70702100
H	-1.11752900	1.04112800	-1.55242800
H	0.85975900	4.46145600	-0.45286000
H	0.74209800	4.36418400	1.31938600
H	5.22134800	-1.94181300	2.41409400
H	6.05381500	-0.87754400	-1.65515800
H	4.58619700	0.53120100	-2.88380100
H	3.82469500	1.77919000	-1.88293600
H	2.86624400	0.41205600	-2.45138300
H	3.28926300	-1.10928100	3.53183400
H	2.72410700	0.47897500	2.97792800
H	1.88004600	-0.97924700	2.46392300
H	7.39323400	-2.47062500	1.43171300
H	7.81185500	-1.90906600	-0.19615800
H	6.81751500	-3.35102200	0.01344000
H	-6.01322400	-3.64344200	0.81689800

Re-TS1H

Zero-point correction= 0.530978 (Hartree/Particle)

C	-3.42562200	-1.45744100	1.43583000
C	-6.19208100	-1.37627600	0.91818400
C	-4.33335500	-1.99893000	2.34668700
C	-3.91980500	-0.88205000	0.27397800
C	-5.28968000	-0.83148200	0.01402400
C	-5.70320000	-1.96202000	2.08675400
C	-5.55068100	-0.13361400	-1.30207400
C	-4.24426900	0.62647000	-1.55425000
C	-3.16564700	-0.25258400	-0.87633200
N	-2.02981900	0.56952400	-0.47902800
C	-2.00186100	1.93467400	-0.54673700
C	-3.21227600	2.68150100	-1.01826200
O	-4.35474800	1.86877400	-0.87125700
N	-0.83274800	2.39192300	-0.21099100
N	-0.11762800	1.24793500	0.06804200
C	-0.81313400	0.11724700	-0.09067200
C	1.26350400	1.35748300	0.44383000
C	3.93359500	1.53466100	1.15193900
C	2.21551700	1.44936600	-0.57199000
C	1.59377000	1.34035900	1.79961200
C	2.94424000	1.42968800	2.13221100
C	3.55342900	1.54031200	-0.19003600
C	1.79580800	1.41200400	-2.01650200
C	0.52154200	1.21229400	2.84904600
C	5.38458200	1.65072100	1.54128700

H	-2.35740000	-1.47209500	1.63754900
H	-7.26041700	-1.33613800	0.72783500
H	-3.97160200	-2.44881000	3.26536100
H	-6.39697700	-2.38530500	2.80593800
H	-5.72514900	-0.85828800	-2.10509400
H	-6.39597500	0.55797700	-1.27596100
H	-4.02829300	0.80208800	-2.61617200
H	-2.76863400	-1.00221600	-1.57318000
H	-3.07102300	2.97660400	-2.06832800
H	-3.36333900	3.58094500	-0.42005100
H	3.23015000	1.41531200	3.18141900
H	4.31898600	1.58417800	-0.96089000
H	2.66894100	1.30164800	-2.66313400
H	1.27410600	2.33400500	-2.29360600
H	1.11437800	0.57348300	-2.20331000
H	-0.24840700	1.97842200	2.71835200
H	0.02290800	0.23913900	2.78420300
H	0.94573000	1.31335400	3.84930000
H	5.61804800	0.99462700	2.38404200
H	5.62464600	2.67594600	1.84219600
H	6.03563900	1.37725900	0.70841100
C	-0.32667400	-1.60373900	-0.79705900
H	-1.03785700	-2.17728400	-0.16207500
C	1.08158600	-1.69884200	-0.27907900
H	1.21317700	-1.62819000	0.79996000
C	2.10908400	-1.80407000	-1.12358500
H	1.86487100	-1.83206100	-2.18479000
C	3.53414600	-1.76576200	-0.76775900

C	4.47314800	-1.50372400	-1.77258800
C	3.99200800	-1.90811300	0.54986600
C	5.82518200	-1.35983400	-1.47214500
H	4.12976500	-1.39605800	-2.79836200
C	5.34032800	-1.77129100	0.85098300
H	3.28465400	-2.12365000	1.34489800
C	6.26363400	-1.49045300	-0.15757000
H	6.53551300	-1.14901700	-2.26551800
H	5.67678000	-1.88362900	1.87732700
O	-0.55394000	-1.49576200	-2.02997200
H	7.31675500	-1.38136400	0.08180700

Si-TS1Cl

Zero-point correction= 0.521020 (Hartree/Particle)

C	4.01179200	-3.00819000	-0.19357800
C	2.78488500	-2.72409200	0.39263600
C	2.62883900	-1.62348700	1.24482100
C	3.75087800	-0.83427300	1.52334900
C	4.98391000	-1.09963100	0.94023900
C	5.09888500	-2.18296800	0.07777800
H	4.13027000	-3.86022900	-0.85364500
H	1.93669900	-3.37031700	0.18800800
H	3.64577700	0.02477500	2.18024100
H	5.84345700	-0.46979600	1.14027600
C	1.32175800	-1.20530700	1.76938500
H	1.31791500	-0.55065800	2.64042700
C	0.14278700	-1.44616100	1.19181700
C	-1.11745600	-0.80478400	1.70563500

H	0.04728000	-2.05969200	0.29662300
O	-1.07932700	-0.00510500	2.67212200
H	-2.01055100	-1.44850800	1.55677200
C	1.76715100	0.36770300	-1.10407200
C	3.76154700	2.34085400	-0.88382900
C	2.98458700	0.22075700	-1.76782100
C	1.56963900	1.49688200	-0.32283100
C	2.54509000	2.49076900	-0.22978000
C	3.97783700	1.19258200	-1.64692800
C	2.04929700	3.63839600	0.62331800
C	0.53523200	3.40740500	0.65889700
C	0.39941300	1.87239900	0.56063600
N	-0.92349800	1.52407000	0.05859700
C	-1.79956500	2.43050100	-0.47512800
C	-1.40492500	3.87177100	-0.60766700
O	-0.00520000	4.00412500	-0.51391700
N	-2.94726700	1.87504900	-0.72825900
N	-2.75751300	0.57319300	-0.31930100
C	-1.54272900	0.32335900	0.17570700
C	-3.84545500	-0.35979600	-0.35851000
C	-5.93090200	-2.17835800	-0.40272100
C	-4.67472500	-0.44612800	0.76348100
C	-4.01781100	-1.14531200	-1.49793200
C	-5.07516000	-2.05423200	-1.49804500
C	-5.71550500	-1.37178100	0.71697100
C	-4.43943500	0.43539700	1.96201900
C	-3.08257500	-1.00890000	-2.66954000
C	-7.08499300	-3.14706600	-0.43393700

H	0.99353700	-0.39053800	-1.18515800
H	4.52649000	3.10869700	-0.81434100
H	3.16662200	-0.66398500	-2.36944900
H	4.92489700	1.05714600	-2.15988400
H	2.45896700	3.58998200	1.63871000
H	2.27800900	4.62353600	0.20981800
H	0.04053000	3.81515000	1.55031000
H	0.47442000	1.40839800	1.55212000
H	-1.90943800	4.45072300	0.17983000
H	-1.71105600	4.26510900	-1.57810500
H	-5.23146500	-2.68036200	-2.37290900
H	-6.37400800	-1.46547700	1.57704600
H	-5.16682900	0.21523400	2.74513400
H	-4.54337400	1.48925300	1.68363600
H	-3.43083000	0.30691300	2.37386500
H	-3.39013900	-1.66119800	-3.48821200
H	-3.06209600	0.02230500	-3.03399800
H	-2.05862900	-1.27170200	-2.38444000
H	-6.90475600	-3.95557800	-1.14559700
H	-8.00693700	-2.63868600	-0.73407400
H	-7.25765100	-3.58647800	0.55132800
Cl	6.63842900	-2.51719000	-0.68079200

Re-TS1Cl

Zero-point correction= 0.521326 (Hartree/Particle)

C	-3.81231400	-1.82903900	1.09295600
C	-6.59594900	-1.73287500	0.68010200
C	-4.67165300	-2.60426600	1.87222700

C	-4.36310000	-1.01485600	0.11357200
C	-5.74180700	-0.95621400	-0.09190700
C	-6.05002300	-2.55899400	1.66345700
C	-6.06916300	0.02191000	-1.19788800
C	-4.79540800	0.86770900	-1.29946200
C	-3.66792400	-0.10393100	-0.87368400
N	-2.54596900	0.64539600	-0.32291900
C	-2.55936100	1.99063400	-0.07836200
C	-3.80781700	2.78035000	-0.32990900
O	-4.91897400	1.91275700	-0.34352100
N	-1.39235400	2.40074700	0.31907000
N	-0.63520200	1.24917600	0.31037500
C	-1.30461800	0.15997700	-0.08104200
C	0.76138100	1.33105300	0.63532000
C	3.46056400	1.50107000	1.23460300
C	1.64348500	1.71903000	-0.37323200
C	1.17689500	1.00752500	1.92788700
C	2.54012600	1.09564900	2.20467500
C	2.99617400	1.80473800	-0.04505400
C	1.13639200	1.98702100	-1.76439200
C	0.17852500	0.57254700	2.96781900
C	4.92421500	1.62014100	1.57305600
H	-2.73796000	-1.84976900	1.25868300
H	-7.67081600	-1.68943000	0.53198500
H	-4.26547800	-3.24407500	2.64866800
H	-6.70566400	-3.16596000	2.27944000
H	-6.25128700	-0.49911400	-2.14432300
H	-6.93250200	0.65669400	-0.98529500

H	-4.62086400	1.29264000	-2.29655400
H	-3.27287400	-0.65291800	-1.73846900
H	-3.71377400	3.31548000	-1.28617000
H	-3.96448900	3.51061900	0.46495100
H	2.89175500	0.84621800	3.20323300
H	3.70714100	2.09204200	-0.81581400
H	1.97050400	2.13567700	-2.45327100
H	0.50786400	2.88323400	-1.78456800
H	0.52873400	1.14665800	-2.12326400
H	-0.61801200	1.31423300	3.07936900
H	-0.29851500	-0.37210500	2.68513700
H	0.66318600	0.43576800	3.93579400
H	5.26196600	0.76810700	2.16987600
H	5.11528300	2.52855900	2.15376500
H	5.53644400	1.66005800	0.66908100
C	-0.79025000	-1.34151100	-1.18845500
H	-1.49567300	-2.05610400	-0.70998000
C	0.61387700	-1.52393500	-0.68750300
H	0.72824300	-1.71415700	0.37896200
C	1.65654800	-1.38555800	-1.50775100
H	1.43426200	-1.15880900	-2.54947100
C	3.06867200	-1.38362900	-1.10543700
C	4.02072800	-0.83638600	-1.97333200
C	3.49865500	-1.83180200	0.15119100
C	5.35221800	-0.69668400	-1.59536800
H	3.70566600	-0.48933900	-2.95365400
C	4.82351400	-1.70644000	0.54312500
H	2.78811500	-2.27994400	0.83823100

C	5.73829100	-1.12765200	-0.33258000
H	6.08123400	-0.25740100	-2.26717500
H	5.15081400	-2.04842100	1.51900100
O	-1.01538500	-0.94533900	-2.35883700
Cl	7.40006000	-0.93344100	0.17395500

RR-TS4H

Zero-point correction= 0.783401 (Hartree/Particle)

C	-0.12017300	-0.24874300	-0.95763900
C	-0.55666500	2.46912400	-0.05639800
C	1.57262400	2.02489300	-1.15612000
C	0.30473200	2.57820800	-1.19998900
C	1.26492300	-0.25414400	-1.13849200
O	-0.24043100	1.74304400	0.90571800
O	-0.92802900	0.19336600	-1.81368500
Si	2.86184100	2.34069000	0.19326400
C	3.98097600	3.61829400	-0.64783700
C	3.97804300	0.88258400	0.60541100
C	2.20945700	3.14389400	1.76156800
C	1.79454700	-0.46854000	-2.54775900
C	3.30391600	-0.51302800	-2.56244500
C	6.11073500	-0.59600800	-2.44366000
C	4.07342700	0.48273400	-3.16552700
C	3.96536600	-1.56105200	-1.91186700
C	5.35337000	-1.60643200	-1.85123800
C	5.46674000	0.44667700	-3.10328300
C	-1.89642700	3.14512200	-0.03601700
C	-4.39525200	4.39714200	0.13058200

C	-2.22878700	4.18176300	-0.91340800
C	-2.82471800	2.75106800	0.93250000
C	-4.06746400	3.37077000	1.01552700
C	-3.47193000	4.80202100	-0.83185300
H	2.07004500	2.02374500	-2.12893900
H	-0.13566200	2.88522500	-2.14288400
H	1.89998900	-0.61124700	-0.33866100
H	4.79488500	3.91888200	0.02045900
H	3.42233500	4.51713800	-0.92660700
H	4.43088600	3.20123700	-1.55544600
H	4.64967400	1.17488600	1.42178300
H	4.59289300	0.61763800	-0.26099100
H	3.43793200	-0.00975500	0.92719300
H	3.04847200	3.58498500	2.31109000
H	1.50092900	3.94212500	1.52131400
H	1.69624500	2.43129400	2.40679000
H	1.41236900	-1.42845100	-2.92893100
H	1.41197500	0.29515800	-3.23037000
H	3.57899300	1.29702600	-3.68967200
H	3.37650700	-2.34257100	-1.43439400
H	5.84635100	-2.42581800	-1.33664400
H	6.04714500	1.23487700	-3.57291700
H	-5.36303200	4.88449500	0.19458200
H	-1.50594200	4.51863300	-1.64947000
H	-2.55558000	1.94972300	1.61471100
H	-4.77900000	3.04900500	1.77086800
H	-3.71846300	5.60799400	-1.51561800
C	-0.76321700	-0.70592500	0.32030700

N	-0.27723500	-1.28345700	1.43378900
N	-2.11122200	-0.71737100	0.49489100
N	-1.26442400	-1.61102100	2.32428700
C	1.07645900	-1.54297100	1.83571200
C	-2.35724900	-1.26454500	1.72200200
C	-3.22484700	-0.39735300	-0.42420200
C	1.77138600	-2.59098100	1.22112800
C	1.59503200	-0.80683400	2.90489700
C	-3.76381400	-1.31422400	2.21537500
H	-2.89514700	0.44139800	-1.03224200
C	-4.53826200	-0.05602500	0.37753100
C	-3.59031100	-1.61154600	-1.25180800
C	3.07608100	-2.82872100	1.64666700
C	1.12892200	-3.43185500	0.15014800
C	2.89737600	-1.10476400	3.30938400
C	0.77107800	0.22891800	3.62126500
H	-3.77583900	-1.33894900	3.30492700
H	-4.27854600	-2.20660100	1.83130500
O	-4.39157700	-0.12451700	1.78722800
C	-5.63535600	-1.01284200	-0.15086000
H	-4.79509100	0.98479100	0.18631500
C	-4.92605000	-1.96477700	-1.07654600
C	-2.75605600	-2.34401000	-2.09378200
C	3.65802100	-2.08783000	2.67869000
H	3.64700200	-3.62259400	1.17056900
H	0.96698000	-2.85063600	-0.76469100
H	0.15330500	-3.80393000	0.47840900
H	1.76013900	-4.28860900	-0.09244300

H	3.33137200	-0.53704900	4.12864300
H	0.05093600	-0.25874700	4.28551400
H	0.21178000	0.84922600	2.91522100
H	1.41534000	0.86987700	4.22642700
H	-6.13425400	-1.51689000	0.68401600
H	-6.40721300	-0.44799300	-0.68225700
C	-5.45089300	-3.07218200	-1.73925700
C	-3.28206000	-3.45341500	-2.74942300
H	-1.72755200	-2.03313800	-2.24514500
C	5.09278800	-2.32103300	3.07021700
C	-4.61966100	-3.81652200	-2.57126500
H	-6.49204100	-3.35148300	-1.60675500
H	-2.65027300	-4.03731100	-3.41077000
H	5.75457300	-1.72190300	2.43532100
H	5.27457400	-2.03160700	4.10739200
H	5.37419500	-3.36945400	2.94691000
H	-5.01472300	-4.68389500	-3.09047300
H	7.19440400	-0.62403400	-2.39342200

RS-TS4H

Zero-point correction= 0.782312 (Hartree/Particle)

C	-0.24981300	0.00274000	0.44054800
C	0.93821400	2.61972900	-0.15428600
C	-1.46143700	2.43527400	0.11743800
C	-0.25505700	2.90199400	0.60314800
C	-1.59391400	0.29058300	0.71723500
O	0.90562700	2.06027300	-1.26676500
O	0.69048300	0.28517600	1.22866900

C	-1.93921500	0.34066800	2.19051700
C	-1.98293100	-1.07998600	2.71010300
C	-2.01850200	-3.79103800	3.44079800
C	-3.15326000	-1.83588900	2.59527500
C	-0.82889200	-1.69864500	3.20522800
C	-0.84709800	-3.04492100	3.56347300
C	-3.17452900	-3.18002900	2.95979600
C	2.27754600	3.01019900	0.40337800
C	4.82754600	3.65148200	1.35098300
C	2.55655500	2.94489500	1.77112600
C	3.28831100	3.38634100	-0.48615900
C	4.55650600	3.71278300	-0.01544300
C	3.82780100	3.26325100	2.24114200
H	-0.17019500	3.38868700	1.57013200
H	-2.38332600	-0.02876900	0.04860800
H	-1.17811000	0.92615500	2.71492400
H	-2.91178100	0.81373200	2.34840400
H	-4.05325400	-1.36520500	2.20446800
H	0.08473400	-1.11590700	3.28190200
H	0.05612800	-3.51190400	3.94576000
H	-4.09349700	-3.75142000	2.86639900
H	5.81736200	3.90024400	1.72072900
H	1.78609700	2.59585500	2.45138700
H	3.05638500	3.41877100	-1.54673900
H	5.33287900	4.01602200	-0.71124000
H	4.04320800	3.19622200	3.30284800
C	0.18157200	-0.57483100	-0.86986100
N	-0.52683900	-1.18405900	-1.83157900

N	1.46778500	-0.75839400	-1.23451500
N	0.26240100	-1.72024500	-2.81077800
C	-1.95233700	-1.29032000	-1.90882400
C	1.46936800	-1.45245900	-2.40741400
C	2.72457600	-0.35340000	-0.60384500
C	-2.57937700	-2.28808500	-1.16130600
C	-2.63731200	-0.33395600	-2.66112200
C	2.78988100	-1.68804600	-3.06117200
H	2.51290900	0.51380500	0.01729500
C	3.80989500	-0.01743400	-1.68286600
C	3.31479600	-1.49111600	0.19556700
C	-3.97382000	-2.29111000	-1.15512800
C	-1.77379800	-3.26058600	-0.34352300
C	-4.02918000	-0.38999900	-2.63294100
C	-1.88937900	0.70664700	-3.45226200
H	2.65056800	-1.94352400	-4.11142700
H	3.32937400	-2.50289000	-2.55788200
O	3.49633400	-0.46681500	-2.99578700
C	5.11077600	-0.65869600	-1.14387800
H	3.89489000	1.06496900	-1.78049900
C	4.65848000	-1.67228100	-0.12078600
C	2.66977700	-2.29009800	1.13362000
C	-4.70951300	-1.34508600	-1.87166100
H	-4.49410200	-3.04173100	-0.56535000
H	-1.33165400	-2.75672300	0.52623800
H	-0.96212900	-3.69494200	-0.93456900
H	-2.40686800	-4.06608300	0.03149500
H	-4.59754400	0.34229600	-3.20194600

H	-1.43454100	0.25729500	-4.34081700
H	-1.07848900	1.15658400	-2.86821300
H	-2.56598800	1.49929100	-3.77708300
H	5.68640500	-1.09026200	-1.96812900
H	5.73538900	0.10872500	-0.67327700
C	5.38956900	-2.67777900	0.50601800
C	3.40295900	-3.29910200	1.75380400
H	1.62435700	-2.11611000	1.37661900
C	-6.21406400	-1.32073300	-1.80284400
C	4.75126500	-3.49069200	1.44106200
H	6.43771100	-2.83163500	0.26689600
H	2.92454700	-3.94055400	2.48705800
H	-6.54918400	-0.50127900	-1.15801500
H	-6.65219300	-1.16092400	-2.79117700
H	-6.60972600	-2.25317900	-1.39569500
H	5.30781300	-4.28280000	1.93189200
H	-1.41697000	2.13009700	-0.92963200
Si	-3.12401300	3.14363500	0.63406300
C	-4.52730000	1.93118700	0.28225900
H	-4.50859700	1.03412700	0.90718700
H	-5.49444500	2.42404500	0.42551100
H	-4.46934800	1.60357600	-0.76206100
C	-3.41204100	4.64217500	-0.47563200
H	-3.41338100	4.35245200	-1.53111500
H	-4.37441800	5.11496500	-0.25425700
H	-2.62387800	5.38715300	-0.33449100
C	-3.10691200	3.73193600	2.42270300
H	-2.35771000	4.52019800	2.54711600

H	-4.08000300	4.15766600	2.68747100
H	-2.87905900	2.93727700	3.13650900
H	-2.03144500	-4.83884700	3.72343800

SR-TS4H

Zero-point correction= 0.782534 (Hartree/Particle)

C	0.22447600	-0.18528300	0.68603700
C	-0.68021300	2.45232800	-0.38883400
C	1.67667600	1.97063000	-0.03288900
C	0.54871900	2.68661600	0.32347800
C	1.55538800	0.05822200	1.05009000
O	-0.74868600	1.71125700	-1.38900600
C	1.75359500	0.49003300	2.48648500
C	1.51382600	-0.67184700	3.42636500
C	1.05865900	-2.89021300	5.08455600
C	2.53039500	-1.59919700	3.67522500
C	0.26330500	-0.87185200	4.01871900
C	0.03865600	-1.97286600	4.84221200
C	2.30793000	-2.70055900	4.49713000
C	-1.91463000	3.17970400	0.05654300
C	-4.23805800	4.52806600	0.82079300
C	-2.79593100	3.66909800	-0.91032100
C	-2.21091800	3.35674300	1.40966300
C	-3.37180000	4.02437700	1.78931100
C	-3.94816200	4.35039500	-0.53109800
H	0.54338500	3.36655600	1.17051300
H	2.36456600	-0.47848600	0.56811200
H	2.76856300	0.87020900	2.63424800

H	1.05066800	1.30122700	2.70243300
H	3.50732200	-1.45077800	3.21886200
H	-0.53128400	-0.16184800	3.81132600
H	-0.93593500	-2.11270900	5.30038300
H	3.11055000	-3.40740400	4.68439700
H	-5.14056700	5.05249700	1.11882000
H	-2.56042500	3.50381000	-1.95805100
H	-1.54563800	2.92975300	2.15408300
H	-3.60632500	4.14555300	2.84224900
H	-4.62281000	4.74007600	-1.28766600
O	-0.76233100	0.32188400	1.27609600
C	-0.10275000	-1.02624400	-0.50852400
N	0.62165700	-1.46343100	-1.55231400
N	-1.30747700	-1.62089000	-0.66195800
N	-0.08846600	-2.31220600	-2.36061800
C	2.01746200	-1.27865900	-1.85032500
C	-1.25486700	-2.38036100	-1.79493000
C	-2.50579000	-1.66907000	0.17954200
C	2.38460300	-0.34301600	-2.81910500
C	2.92859500	-2.14120100	-1.22945800
C	-2.41699900	-3.27848900	-2.06048100
H	-2.22551600	-1.27403900	1.15622600
C	-3.03313900	-3.13795200	0.27874700
C	-3.67513600	-0.91044500	-0.40820800
C	3.74694200	-0.23127100	-3.10583000
C	1.35251700	0.46473400	-3.55728800
C	4.27376800	-1.99387900	-1.55800100
C	2.46291000	-3.18488300	-0.24804700

H	-2.14548500	-4.03262100	-2.79871500
H	-3.28295500	-2.70888200	-2.41838800
O	-2.68247100	-3.95043700	-0.84024500
C	-4.56122400	-2.97702800	0.40030500
H	-2.59884800	-3.64923700	1.13992600
C	-4.84644400	-1.65361700	-0.26779200
C	-3.66584900	0.34471100	-1.00096700
C	4.70045700	-1.03609100	-2.48209100
H	4.06517000	0.49622600	-3.84842600
H	0.63504500	0.93201300	-2.87545500
H	0.79004900	-0.18563600	-4.23573400
H	1.83547500	1.24227000	-4.15214000
H	5.00535600	-2.64258100	-1.08299600
H	2.06486200	-2.72536600	0.66446500
H	3.28951300	-3.83791200	0.03530100
H	1.66968300	-3.80133000	-0.68226200
H	-5.06717500	-3.82833100	-0.06242900
H	-4.85241700	-2.95005900	1.45674000
C	-6.05539600	-1.13557100	-0.71746400
C	-4.88234300	0.85770500	-1.45188100
H	-2.73503200	0.89327800	-1.12832300
C	6.16741800	-0.85310300	-2.76855700
C	-6.06340800	0.12759600	-1.30953200
H	-6.97427700	-1.70592300	-0.61863500
H	-4.90651600	1.83913600	-1.91457400
H	6.32883500	-0.43394100	-3.76384400
H	6.60987100	-0.16408500	-2.04079500
H	6.70692100	-1.80023600	-2.69767700

H	-6.99874100	0.54410000	-1.67014700
Si	3.41905700	2.57403100	0.33895200
H	1.58588800	1.46369500	-0.99452700
C	3.93234600	3.56393900	-1.18232600
H	3.23823800	4.38870900	-1.36633000
H	4.93817100	3.97960300	-1.06444100
H	3.93398300	2.92073900	-2.06902700
C	4.65257700	1.15871200	0.50804800
H	4.53988200	0.57938800	1.42826600
H	4.53758900	0.47191600	-0.33740500
H	5.67405800	1.55300900	0.48297900
C	3.43589300	3.69636900	1.85010300
H	2.81801000	4.58174100	1.67198100
H	3.05713200	3.19765200	2.74616300
H	4.45478600	4.03744400	2.05858100
H	0.88314800	-3.74527600	5.72966600

SS-TS4H

Zero-point correction= 0.782831 (Hartree/Particle)

C	0.06853700	0.53659600	0.57858500
C	-2.87328500	1.40249900	0.46644900
C	-1.15762700	3.11308000	0.52772300
C	-2.19720600	2.45809800	1.16151800
C	0.60137700	1.82592300	0.78372900
O	-2.53358500	1.05774300	-0.68094900
Si	-1.11888400	3.52316200	-1.32243300
C	-2.84799700	3.91537700	-1.95558200
C	-0.25733500	2.35055100	-2.52218700

C	-0.10965900	5.12810300	-1.38014800
C	0.85826700	2.21835700	2.22600500
C	1.93144400	1.35611700	2.85516100
C	3.92051100	-0.26483900	3.99710700
C	3.28706000	1.65297000	2.67850100
C	1.58621500	0.22650800	3.60503300
C	2.57400600	-0.57514800	4.17435700
C	4.27585800	0.85021200	3.24131300
C	-3.98990400	0.69420300	1.17162900
C	-6.04907400	-0.69089300	2.45345800
C	-4.11858000	-0.68427700	1.00862200
C	-4.89870300	1.37830700	1.98187000
C	-5.93125700	0.68838600	2.61294600
C	-5.13572500	-1.37762200	1.65363600
H	-0.68063900	3.89226600	1.12767900
H	-2.39540900	2.59130000	2.22107400
H	1.24320400	2.26222200	0.02542600
H	-2.79869700	4.32616900	-2.96947000
H	-3.33668900	4.65599400	-1.31571800
H	-3.46308000	3.01335600	-1.97100400
H	-0.39942100	2.74440800	-3.53504000
H	0.82278800	2.28396600	-2.35261800
H	-0.69450700	1.35123700	-2.48347700
H	-0.58457500	5.91788100	-0.78906300
H	0.89819600	4.97486800	-0.97688200
H	-0.00347100	5.49588300	-2.40579300
H	1.15611700	3.27117900	2.27671400
H	-0.07313900	2.10470700	2.78960100

H	3.56635600	2.53604800	2.10597000
H	0.53506700	-0.01657400	3.72732700
H	2.28977500	-1.44351700	4.76159000
H	5.32309300	1.09767200	3.09609100
H	-6.84849000	-1.22860100	2.95407100
H	-3.38964100	-1.19338500	0.39350400
H	-4.80589000	2.45410200	2.10234500
H	-6.64351800	1.22664000	3.23058000
H	-5.20976400	-2.45584500	1.53848300
O	-0.66745400	-0.05964300	1.39335800
C	0.45246100	-0.17049800	-0.69796700
N	-0.20460500	-1.09977800	-1.41032300
N	1.65774200	-0.05196300	-1.31969200
N	0.52261900	-1.53238400	-2.48689300
C	-1.42462100	-1.83189600	-1.16061100
C	1.63978400	-0.88400300	-2.40397500
C	2.97025600	0.42345600	-0.84257400
C	-1.38927200	-2.81666900	-0.16858800
C	-2.48888100	-1.69334400	-2.06066800
C	2.83326200	-0.90272100	-3.30251800
H	2.82256600	1.26550500	-0.17254800
C	3.89017700	0.81606300	-2.05396300
C	3.70600400	-0.69681400	-0.14834800
C	-2.51608400	-3.63078300	-0.03270000
C	-0.18331600	-3.02380000	0.70606400
C	-3.57998900	-2.54053400	-1.88479200
C	-2.49006900	-0.67592100	-3.16713200
H	2.53114900	-1.14906900	-4.32039300

H	3.56611500	-1.64530200	-2.95665700
O	3.36627100	0.40473200	-3.30547800
C	5.26295400	0.16905000	-1.74481600
H	3.96089300	1.90094700	-2.13699000
C	4.99035600	-0.84702000	-0.66309600
C	3.22758800	-1.49420600	0.88362200
C	-3.61776900	-3.50489900	-0.87564200
H	-2.51756800	-4.39342100	0.74170600
H	-0.09993500	-2.19983100	1.42134200
H	0.73616900	-3.05759600	0.11021200
H	-0.26723400	-3.96659000	1.24923300
H	-4.42672700	-2.44320800	-2.55975500
H	-1.52181500	-0.62333500	-3.67040100
H	-2.71907700	0.30545800	-2.74439500
H	-3.24879100	-0.93109200	-3.90938300
H	5.68328100	-0.26341200	-2.65818100
H	5.96857000	0.92961100	-1.39529200
C	5.82878100	-1.83182800	-0.14775000
C	4.06739800	-2.48075400	1.39336000
H	2.23486800	-1.33427300	1.30040700
C	-4.82402500	-4.39514200	-0.72415700
C	5.35487400	-2.64881100	0.87780000
H	6.83250900	-1.96589200	-0.53962200
H	3.72214900	-3.11184700	2.20574000
H	-5.73530500	-3.79756500	-0.62798500
H	-4.94644500	-5.03939900	-1.60011200
H	-4.73775100	-5.03372200	0.15764400
H	5.99698000	-3.42357900	1.28429400

H	4.68922600	-0.89100300	4.43907800

RR-TS4Cl

Zero-point correction= 0.773642 (Hartree/Particle)

C	-0.37808300	-0.26080400	-0.88816200
C	-0.97678700	2.46669500	-0.08705700
C	1.24251200	2.06731100	-1.01024600
C	-0.03587700	2.57648800	-1.16575200
C	1.01679300	-0.21223800	-0.95735900
O	-0.71320100	1.76915200	0.91168400
O	-1.12817700	0.12367600	-1.82016800
Si	2.40859900	2.47106800	0.42543500
C	3.54512300	3.76521900	-0.36450100
C	3.53884200	1.06495200	0.96394400
C	1.60728200	3.28857300	1.91465100
C	1.65934000	-0.44274900	-2.31641600
C	3.16475500	-0.44462600	-2.21004400
C	5.93493800	-0.44406300	-1.86132500
C	3.95567100	0.55758300	-2.77219400
C	3.79932000	-1.45632300	-1.48053600
C	5.17652600	-1.46730600	-1.29988500
C	5.33941800	0.56971800	-2.60011000
C	-2.33051000	3.10828100	-0.17810400
C	-4.86353700	4.29998500	-0.21591400
C	-2.62677600	4.11169600	-1.10569100
C	-3.31257900	2.71733500	0.73707100
C	-4.57243700	3.30706200	0.71850600
C	-3.88694100	4.70189800	-1.12570900

H	1.81404800	2.06332600	-1.94176400
H	-0.41143900	2.84818100	-2.14667100
H	1.59934900	-0.52623300	-0.10134100
H	4.29424700	4.11223800	0.35494800
H	2.97925900	4.63556900	-0.71075700
H	4.07879100	3.34281600	-1.22297200
H	4.14342900	1.40421200	1.81356900
H	4.22195300	0.79570800	0.15181600
H	3.00635700	0.16484600	1.27720300
H	2.38358600	3.78309900	2.50889700
H	0.88413900	4.04620500	1.59865800
H	1.08016600	2.57186300	2.54401100
H	1.33425600	-1.42011700	-2.70547000
H	1.31295800	0.29621800	-3.04394800
H	3.48966800	1.34744200	-3.35533600
H	3.19816800	-2.24405300	-1.03072200
H	5.66048900	-2.25131400	-0.72774000
H	5.94794100	1.35509900	-3.03440800
H	-5.84462400	4.76414700	-0.23110300
H	-1.86379000	4.44686500	-1.80087400
H	-3.07123100	1.94230500	1.45883300
H	-5.32581700	2.98796300	1.43324700
H	-4.10561300	5.48245300	-1.84736200
C	-1.10461900	-0.70852500	0.34780400
N	-0.68672900	-1.25019400	1.50557300
N	-2.46060400	-0.75032200	0.42467500
N	-1.72747500	-1.58276000	2.33050200
C	0.64065300	-1.47322200	2.00526200

C	-2.78191200	-1.27637600	1.64360500
C	-3.51191500	-0.47732000	-0.57868800
C	1.39977300	-2.51410000	1.45811300
C	1.06779900	-0.70799700	3.09417200
C	-4.21916600	-1.34844800	2.03601600
H	-3.15775300	0.35470500	-1.18218600
C	-4.88627700	-0.14744400	0.11894200
C	-3.79140200	-1.71885500	-1.39924600
C	2.67659200	-2.71487000	1.97730300
C	0.85389800	-3.38488800	0.35783600
C	2.34461000	-0.96989700	3.59374200
C	0.17604500	0.32557200	3.72735600
H	-4.30940300	-1.35005700	3.12217800
H	-4.68544900	-2.26054900	1.63670900
O	-4.83975900	-0.18292100	1.53672300
C	-5.92280200	-1.13900900	-0.46443300
H	-5.14981200	0.88317300	-0.11350900
C	-5.12919600	-2.09639700	-1.31278600
C	-2.88325400	-2.45362500	-2.15861600
C	3.16893500	-1.94505700	3.03468800
H	3.29592500	-3.50427400	1.55736700
H	0.75192100	-2.82314000	-0.57773200
H	-0.13766100	-3.76844400	0.61779600
H	1.51517600	-4.23483400	0.17975000
H	2.70834500	-0.37888300	4.43051400
H	-0.57778400	-0.16131800	4.35365500
H	-0.34655700	0.91585500	2.96934400
H	0.76443200	0.99516600	4.35783600

H	-6.47131800	-1.63451300	0.34401400
H	-6.66471600	-0.60285900	-1.06380700
C	-5.58220500	-3.23004900	-1.98401600
C	-3.33787000	-3.58922400	-2.82270600
H	-1.85255100	-2.12488900	-2.24185200
C	4.57681700	-2.13989800	3.53077400
C	-4.67777100	-3.97606800	-2.73408200
H	-6.62464700	-3.52807100	-1.92080100
H	-2.64791800	-4.17531100	-3.42096300
H	5.26722800	-1.51943900	2.94908700
H	4.67282200	-1.85025200	4.57931600
H	4.89592700	-3.17944200	3.42621700
H	-5.01661800	-4.86367000	-3.25877900
Cl	7.66906600	-0.43900200	-1.63466900

RS-TS4Cl

Zero-point correction= 0.772494 (Hartree/Particle)

C	-0.14956500	-0.18018500	-0.32528100
C	1.51668900	-2.59284600	-0.51763900
C	-0.89212500	-2.79335200	-0.65618400
C	0.33399100	-2.87936400	-1.28898400
C	-1.43964300	-0.64230700	-0.62652100
O	1.47490800	-2.36093100	0.70574900
O	0.75310700	-0.05160200	-1.19283600
C	-1.89124800	-0.36364900	-2.04505600
C	-2.21502300	1.10971400	-2.14983000
C	-2.75492000	3.84490900	-2.08250000
C	-3.47910300	1.58124000	-1.78516500

C	-1.22764900	2.03997400	-2.49403600
C	-1.48945500	3.40709800	-2.45858700
C	-3.76023900	2.94397100	-1.75104000
C	2.85583000	-2.56828700	-1.19812000
C	5.39678200	-2.45214100	-2.35688200
C	3.01404300	-2.08522900	-2.49982400
C	3.98077300	-2.98078300	-0.47778300
C	5.24566100	-2.93028100	-1.05574100
C	4.28070100	-2.02626200	-3.07475200
H	0.42385800	-3.06677100	-2.35475600
H	-2.21509200	-0.66790100	0.12881500
H	-1.08819400	-0.63766500	-2.73554000
H	-2.77975100	-0.94865300	-2.29507500
H	-4.25499900	0.87094200	-1.50860000
H	-0.23889900	1.68142900	-2.76391700
H	-0.72293900	4.12789900	-2.72256400
H	-4.74245500	3.30637100	-1.46753800
H	6.38292600	-2.40656600	-2.80824400
H	2.14635400	-1.71202000	-3.03449800
H	3.84047500	-3.34214300	0.53680300
H	6.11301900	-3.26425000	-0.49454900
H	4.39940100	-1.63605700	-4.08059500
C	0.28412100	0.09968000	1.07846200
N	-0.43798800	0.29308600	2.19113200
N	1.54582500	0.41283600	1.43869200
N	0.32512400	0.68664800	3.25480700
C	-1.85106800	0.11500000	2.33593400
C	1.52422700	0.76054500	2.75655300

C	2.79949700	0.42281500	0.68391200
C	-2.69077000	1.14642700	1.91366700
C	-2.30758500	-1.11661000	2.81006900
C	2.83493200	1.04986800	3.40855000
H	2.68578600	-0.26926100	-0.14743700
C	4.00414200	0.00810200	1.59562100
C	3.13436200	1.81942500	0.21576400
C	-4.06277500	0.89920500	1.94880800
C	-2.12259800	2.43272100	1.37808900
C	-3.68737600	-1.30759400	2.83801300
C	-1.33821000	-2.17944100	3.25593000
H	2.73819200	0.99019900	4.49240300
H	3.19096300	2.05186600	3.13047300
O	3.72557100	0.03645400	2.99057500
C	5.14059200	0.98423300	1.21010400
H	4.26654700	-1.03028500	1.39280300
C	4.45291500	2.14117600	0.52642800
C	2.29770300	2.71585700	-0.44025100
C	-4.57369600	-0.32152400	2.39423700
H	-4.74470100	1.67479900	1.60909400
H	-1.66458000	2.26847500	0.39361600
H	-1.35445700	2.83305600	2.04606500
H	-2.90695900	3.18158400	1.25649800
H	-4.08085900	-2.25637200	3.19591700
H	-0.89901800	-1.91478000	4.22306100
H	-0.51013000	-2.29780100	2.54776800
H	-1.84596500	-3.13998200	3.36155600
H	5.70773100	1.27138200	2.10045700

H	5.83574800	0.49056800	0.52182800
C	4.96168300	3.39058700	0.18248700
C	2.80801200	3.96761000	-0.77627700
H	1.27744500	2.43290300	-0.68719200
C	-6.05448100	-0.59675800	2.36941800
C	4.12909900	4.30092400	-0.46619500
H	5.98746600	3.65559400	0.42112300
H	2.17640300	4.68997700	-1.28344000
H	-6.29793900	-1.28274000	1.55113400
H	-6.38408400	-1.06610500	3.29972700
H	-6.62811800	0.32042700	2.22317200
H	4.51073300	5.28137800	-0.73275800
H	-0.81479900	-2.78118200	0.43257900
Si	-2.44222700	-3.64489700	-1.29444000
C	-3.99974300	-2.85932200	-0.57291100
H	-4.19348500	-1.84388900	-0.92953400
H	-4.87487100	-3.47341500	-0.80896900
H	-3.90884800	-2.81083700	0.51828800
C	-2.36679000	-5.41012600	-0.63376300
H	-2.32922600	-5.41695000	0.46007100
H	-3.24636700	-5.98298900	-0.94491100
H	-1.47483000	-5.92467300	-1.00217900
C	-2.47508900	-3.72132200	-3.17554700
H	-1.60731900	-4.28038500	-3.53961500
H	-3.37233400	-4.24938400	-3.51371000
H	-2.46264500	-2.73736100	-3.64953200
Cl	-3.08864900	5.56227100	-2.02325400

SR-TS4Cl

Zero-point correction= 0.772816 (Hartree/Particle)

C	0.21565100	0.27736600	-0.31202000
C	-0.77818500	-2.41721700	-1.17096500
C	1.59493400	-1.88840900	-1.12570600
C	0.46168300	-2.18427200	-1.86297100
C	1.54735300	0.26696200	-0.75076600
O	-0.85850800	-2.46301000	0.07250400
C	1.76047100	0.83439100	-2.13759000
C	1.53282300	2.32987700	-2.14361200
C	1.09601600	5.08152100	-2.04597300
C	2.54875400	3.20284000	-1.74383800
C	0.29111500	2.86715700	-2.49533400
C	0.06590200	4.24031300	-2.44918000
C	2.34236800	4.57772900	-1.69165400
C	-2.01377300	-2.67467800	-1.98257300
C	-4.34241400	-3.19127500	-3.43600900
C	-2.92583700	-3.63348700	-1.53527100
C	-2.28205200	-1.96529300	-3.15511100
C	-3.44548300	-2.21958200	-3.87563000
C	-4.08031600	-3.89991400	-2.26444500
H	0.46539400	-2.18068600	-2.94913700
H	2.36141800	0.34827900	-0.03935600
H	2.77579000	0.62183500	-2.48429900
H	1.05807400	0.34661800	-2.82148100
H	3.52134800	2.80044800	-1.46912800
H	-0.50673300	2.19360600	-2.79026600
H	-0.89619400	4.65700800	-2.72633500

H	3.13440700	5.25237000	-1.38642100
H	-5.24665200	-3.39191300	-4.00201600
H	-2.71160100	-4.16024500	-0.60961600
H	-1.59243900	-1.18803500	-3.47031900
H	-3.65772500	-1.65402500	-4.77751600
H	-4.77858200	-4.65660500	-1.91909000
O	-0.77074000	0.30765800	-1.08872200
C	-0.11612500	0.16924500	1.14456000
N	0.58754300	-0.21244700	2.22335200
N	-1.30312200	0.58092500	1.64328700
N	-0.12090900	-0.06160400	3.38634600
C	1.96957200	-0.59903800	2.33760700
C	-1.26447100	0.41422300	2.99764100
C	-2.46703800	1.22737100	1.03081000
C	2.28631600	-1.95058300	2.48576500
C	2.91894100	0.42655000	2.41970200
C	-2.40412800	0.97615900	3.78058400
H	-2.16157900	1.56050900	0.03884400
C	-2.94794900	2.42328200	1.91817000
C	-3.67740500	0.32370200	0.96126200
C	3.63697700	-2.26966600	2.64257200
C	1.21569800	-3.00606200	2.53130500
C	4.25029500	0.05397300	2.58651400
C	2.50665100	1.87329100	2.33846700
H	-2.13471300	1.04949100	4.83383600
H	-3.30182900	0.35555800	3.67295800
O	-2.60020000	2.29369700	3.29511500
C	-4.47704900	2.44360600	1.72114600

H	-2.47649900	3.35518000	1.60080400
C	-4.82136300	1.02361800	1.34167200
C	-3.72340500	-1.00756100	0.57061000
C	4.62732200	-1.28816700	2.68504400
H	3.91610000	-3.31494700	2.74963500
H	0.50409800	-2.90193200	1.70637700
H	0.65290000	-2.92128300	3.46708100
H	1.66373300	-4.00072200	2.49161000
H	5.01032600	0.82899900	2.64393400
H	2.10329100	2.11958000	1.34921800
H	3.36103700	2.52480200	2.52706400
H	1.73158400	2.09872900	3.07763400
H	-4.96831800	2.79241500	2.63325500
H	-4.74214300	3.13702400	0.91477300
C	-6.05918000	0.39089700	1.32540700
C	-4.96823800	-1.63688900	0.55857400
H	-2.81292500	-1.54199700	0.30960800
C	6.08032700	-1.66648600	2.79864200
C	-6.12275100	-0.94497400	0.92888800
H	-6.95789500	0.92279000	1.62342700
H	-5.03546800	-2.67575200	0.25211600
H	6.20273200	-2.62521400	3.30676200
H	6.52438700	-1.75859000	1.80143000
H	6.64635500	-0.90821000	3.34421000
H	-7.08128200	-1.45424900	0.91490500
Si	3.33198200	-2.18498300	-1.78616800
H	1.49034700	-2.10563700	-0.06202700
C	3.78084100	-3.92972800	-1.22919800

H	3.06359100	-4.66127000	-1.61151600
H	4.77969100	-4.21229200	-1.57669700
H	3.77349200	-3.99008000	-0.13545800
C	4.60423600	-1.02406100	-1.02021400
H	4.52763100	0.00976400	-1.36763300
H	4.48926900	-1.02208600	0.06902300
H	5.61428900	-1.37989700	-1.24962800
C	3.35938200	-2.09851500	-3.66649400
H	2.71247100	-2.87179700	-4.09173200
H	3.01982500	-1.13099400	-4.04590500
H	4.37379500	-2.27014800	-4.03988400
Cl	0.82174400	6.80995300	-1.98594600

SS-TS4Cl

Zero-point correction= 0.773492 (Hartree/Particle)

C	0.12944200	0.09123200	-0.68502600
C	3.08228600	0.34770800	-1.46241100
C	1.44752900	1.64717100	-2.68270800
C	2.37016400	0.61472100	-2.67496400
C	-0.36111800	0.84710400	-1.77097800
O	2.88472400	1.02070000	-0.43266800
Si	1.69103000	3.29692300	-1.77868500
C	3.50641400	3.79694200	-1.79081400
C	0.98156600	3.53718300	-0.04722100
C	0.74842700	4.52650000	-2.87383300
C	-0.79568900	0.04599000	-2.98378300
C	-1.98453900	-0.83707000	-2.67400100
C	-4.17734700	-2.44996000	-2.06024900

C	-3.28556800	-0.32555200	-2.70924600
C	-1.80695200	-2.17730700	-2.31589600
C	-2.89731700	-2.98889600	-2.01055500
C	-4.38649100	-1.11945200	-2.40292000
C	4.05095700	-0.79573800	-1.44812600
C	5.82553300	-2.94939600	-1.35605100
C	4.02583200	-1.68126700	-0.37186300
C	4.96477100	-0.99641700	-2.48443200
C	5.85837500	-2.06432600	-2.43151900
C	4.90111800	-2.75999600	-0.32899400
H	0.92723600	1.78037300	-3.63498700
H	2.43788200	-0.10367500	-3.48700500
H	-0.87508100	1.78320600	-1.57977500
H	3.62234500	4.81575100	-1.40638800
H	3.91127100	3.77216100	-2.80678600
H	4.09286800	3.11847700	-1.16780200
H	1.27775800	4.53431700	0.29807200
H	-0.11303300	3.50480800	-0.02558000
H	1.38344200	2.80311100	0.65368400
H	1.14198500	4.53505600	-3.89540100
H	-0.31578900	4.26917400	-2.93212400
H	0.82053400	5.54528000	-2.47942900
H	-1.04868800	0.72320300	-3.80634500
H	0.04539600	-0.57612600	-3.30528600
H	-3.44334600	0.71261000	-2.99515900
H	-0.79970800	-2.57947100	-2.26857100
H	-2.75710400	-4.03009800	-1.74005500
H	-5.39343100	-0.71773000	-2.43129400

H	6.51452800	-3.78787000	-1.32218100
H	3.28400700	-1.52609200	0.40022000
H	4.98378500	-0.30572200	-3.32330200
H	6.57863900	-2.20786300	-3.23105200
H	4.85336400	-3.45904100	0.50202400
O	0.70336300	-1.01194600	-0.80228500
C	-0.09565100	0.63226800	0.70556500
N	0.61266100	0.44152800	1.82915400
N	-1.18516200	1.34248100	1.10447600
N	0.03322100	1.04610000	2.91216800
C	1.74436900	-0.40556000	2.12515800
C	-1.05042100	1.57819000	2.44376200
C	-2.52221900	1.51120400	0.50899100
C	1.50543400	-1.77961400	2.22472700
C	2.94954500	0.19024000	2.51576300
C	-2.10068900	2.39182300	3.12830400
H	-2.42574200	1.56578700	-0.57154800
C	-3.22564300	2.79611500	1.07441800
C	-3.42256000	0.36986400	0.91304100
C	2.56409000	-2.58533900	2.64922800
C	0.16342900	-2.38050400	1.90755200
C	3.96778300	-0.66202100	2.93468300
C	3.16842800	1.67741000	2.49165100
H	-1.66729000	2.92324300	3.97584000
H	-2.91500200	1.74739600	3.48888300
O	-2.55521600	3.34892700	2.19483200
C	-4.67222100	2.35062300	1.40241300
H	-3.20467000	3.59418800	0.33127500

C	-4.63348300	0.84199100	1.40979400
C	-3.14934100	-0.98822900	0.80555200
C	3.80098200	-2.04666800	2.99613200
H	2.40631900	-3.65850500	2.72103200
H	0.00495900	-2.37660400	0.82462700
H	-0.64867700	-1.81260800	2.37593100
H	0.11162400	-3.40671200	2.27535900
H	4.92095000	-0.22777600	3.22679000
H	2.28929500	2.22213100	2.84433200
H	3.38468600	1.98814700	1.46644700
H	4.01331700	1.94122200	3.13076700
H	-4.98699300	2.78445000	2.35684900
H	-5.36391500	2.72080900	0.63868000
C	-5.61284700	-0.06237200	1.81207000
C	-4.12770200	-1.88906800	1.21606100
H	-2.20651600	-1.33921100	0.38968200
C	4.94153700	-2.92540000	3.43933000
C	-5.34970400	-1.42742200	1.71212500
H	-6.56433600	0.28840700	2.20033200
H	-3.94781700	-2.95608000	1.13054600
H	5.80145300	-2.79969800	2.77407800
H	5.26830200	-2.66366200	4.45005300
H	4.65670400	-3.97984500	3.43457500
H	-6.10469100	-2.14337100	2.02082200
Cl	-5.55037100	-3.46028900	-1.66489600

Si-TS1'

Zero-point correction= 0.632923 (Hartree/Particle)

C	-1.02743700	3.28874100	0.92486200
C	-3.67468900	4.12070400	0.44566400
C	-1.46010100	4.54910900	1.34050800
C	-1.93200800	2.46270400	0.27719600
C	-3.24318800	2.86968200	0.02566200
C	-2.77328500	4.95686500	1.10777000
C	-3.99077700	1.79030300	-0.72746400
C	-2.85841000	0.93384900	-1.30347900
C	-1.73861400	1.05680200	-0.24506300
N	-0.44124100	0.79043600	-0.83467600
C	-0.21793800	0.79012000	-2.18619700
C	-1.36870300	0.90737200	-3.13390400
O	-2.44936800	1.56418000	-2.50989000
N	1.02696400	0.54440300	-2.45242000
N	1.57706200	0.36153900	-1.20662400
C	0.70790200	0.47056800	-0.18522800
C	2.96661900	0.05616700	-1.07590900
C	5.65726400	-0.54245200	-0.75492300
C	3.42017400	-1.20669800	-1.46717700
C	3.81904800	1.04212100	-0.56666100
C	5.16486400	0.71994300	-0.41571900
C	4.77638000	-1.48513800	-1.28364200
C	2.49867800	-2.21186200	-2.10762200
C	3.29283300	2.40221300	-0.18971800
H	-0.00429000	2.96092100	1.08663700
H	-4.69016300	4.45206600	0.24976900

H	-0.76957900	5.21783600	1.84394800
H	-3.09491700	5.94063300	1.43429800
H	-4.60869400	1.18836200	-0.05129900
H	-4.62940400	2.17211200	-1.52729100
H	-3.13046200	-0.11301800	-1.49327100
H	-1.88940200	0.32072900	0.55411100
H	-1.65349000	-0.11102500	-3.43960100
H	-1.09183800	1.48237100	-4.01820100
H	5.84558200	1.47061500	-0.02096400
H	5.15187000	-2.46433300	-1.57112800
H	2.89025500	-3.22288200	-1.97704900
H	2.42548000	-2.01319500	-3.18197500
H	1.48078500	-2.16481100	-1.70933700
H	4.11668100	3.09050400	0.00694400
H	2.67065300	2.81874400	-0.98729400
H	2.66825300	2.34670000	0.70891700
C	-1.28454200	-2.11220800	-0.02502500
C	-0.65946500	-1.58011600	1.14828800
H	-1.26472800	-1.42688800	2.03798400
C	0.64575600	-1.07483700	1.09709000
H	1.29513700	-1.57493000	0.37573400
O	-0.77652400	-2.02527000	-1.15849400
C	7.10933400	-0.87999000	-0.53498500
H	7.28006300	-1.18912100	0.50133000
H	7.42858600	-1.69891800	-1.18304800
H	7.74912500	-0.01587200	-0.72966500
Si	1.46299000	-0.52287600	2.70856500
C	3.31557700	-0.27839600	2.48129200

H	3.55765000	0.72194500	2.11471400
H	3.82971400	-0.41683400	3.43777200
H	3.72880400	-0.99847300	1.76609500
C	1.18333800	-1.90924900	3.95513300
H	1.64870900	-1.66618300	4.91571700
H	0.11641200	-2.07821200	4.12764900
H	1.61450300	-2.84937000	3.59844100
C	0.63728600	1.05171200	3.32682800
H	0.78364400	1.17854400	4.40366700
H	1.05224100	1.93091600	2.82573600
H	-0.44040100	1.03521900	3.13130900
C	-2.65548600	-2.73057800	0.09498600
C	-3.40217700	-2.90757200	-1.07401500
C	-3.19953200	-3.14265000	1.31535000
C	-4.67808300	-3.45802900	-1.02449000
H	-2.94988700	-2.61220200	-2.01567500
C	-4.47360300	-3.70107000	1.36602800
H	-2.61690300	-3.05194900	2.22642400
C	-5.21827500	-3.85311200	0.19826800
H	-5.25036000	-3.58526300	-1.93807100
H	-4.88372700	-4.02399200	2.31763200
H	-6.21255600	-4.28650600	0.24000500

Re-TS1'

Zero-point correction= 0.633129 (Hartree/Particle)

C	3.66839400	-1.62332900	0.40960800
C	6.13671600	-0.29601800	0.14387600
C	4.86633500	-2.33596700	0.47578500

C	3.72528200	-0.25213900	0.20747700
C	4.94458000	0.41303900	0.07178900
C	6.08862600	-1.67633000	0.34615600
C	4.71583000	1.89376900	-0.13843200
C	3.24685400	1.96338800	-0.57039900
C	2.59656100	0.75038500	0.13547500
N	1.42064600	0.29700600	-0.59309000
C	1.10839000	0.72645600	-1.85679300
C	1.92067400	1.80222400	-2.50458300
O	3.22915000	1.78455400	-1.98101400
N	0.04676700	0.12928100	-2.30352300
N	-0.30636100	-0.69635700	-1.26204200
C	0.48834400	-0.60510400	-0.17627700
C	-1.45944100	-1.53204700	-1.36593900
C	-3.68551800	-3.19729800	-1.46355000
C	-2.72767100	-0.95197700	-1.45957200
C	-1.26577000	-2.92148000	-1.39226400
C	-2.39556400	-3.73448100	-1.42251400
C	-3.82814600	-1.81245200	-1.50381900
C	-2.93222300	0.53674200	-1.57085600
C	0.11802000	-3.51335500	-1.48198200
H	2.70902100	-2.12574700	0.50214900
H	7.09083100	0.21072300	0.03265500
H	4.84744900	-3.41053200	0.62584300
H	7.01227400	-2.24406500	0.39591500
H	4.86118200	2.44961500	0.79463900
H	5.35998100	2.33571400	-0.90230800
H	2.73394100	2.89476300	-0.30168600

H	2.26448300	1.06920300	1.12687000
H	1.43838800	2.77359000	-2.31608000
H	1.98400400	1.63994300	-3.58115200
H	-2.26655600	-4.81412200	-1.43535800
H	-4.82432100	-1.38035900	-1.56563400
H	-3.84761300	0.83897700	-1.05506200
H	-3.02857200	0.82116900	-2.62356300
H	-2.09954800	1.10139200	-1.15349400
H	0.07103400	-4.60330600	-1.44191400
H	0.58783100	-3.22340600	-2.42759400
H	0.76810700	-3.16109400	-0.67901200
C	-0.74283800	2.20265500	1.03387800
C	-1.20728700	0.87962700	1.36102100
H	-2.25747800	0.64553600	1.21875900
C	-0.31713900	-0.14596300	1.66067400
H	0.66704700	0.18532500	1.99908000
O	0.46082100	2.49846100	0.95713700
C	-4.89190500	-4.09947900	-1.44675000
H	-4.69627200	-5.03235500	-1.98082300
H	-5.16161900	-4.35862000	-0.41757200
H	-5.75661000	-3.61387400	-1.90417300
Si	-0.93772000	-1.70897900	2.52218900
C	0.12863800	-3.21184800	2.15257600
H	1.18904600	-2.94670600	2.09903400
H	0.00842400	-3.96081700	2.94170000
H	-0.16218400	-3.67288400	1.20545000
C	-2.72645100	-2.06447400	2.07214400
H	-3.09053400	-2.91132800	2.66340700

H	-3.37354100	-1.20848400	2.28632200
H	-2.83301300	-2.32040400	1.01421600
C	-0.82047900	-1.31454100	4.36275200
H	-1.19846600	-2.14853200	4.96258600
H	0.21557000	-1.12838100	4.66203800
H	-1.40601600	-0.42423600	4.60926600
C	-1.76157900	3.27154700	0.72675200
C	-1.32808700	4.41379400	0.04780800
C	-3.10795400	3.16986300	1.09163300
C	-2.22232700	5.42651700	-0.27921000
H	-0.27700200	4.48500400	-0.21139200
C	-4.00327900	4.18665100	0.77192500
H	-3.45705200	2.30637600	1.64848700
C	-3.56415300	5.31374600	0.08094300
H	-1.87538900	6.30565000	-0.81310000
H	-5.04419700	4.10159200	1.06740200
H	-4.26427600	6.10403600	-0.17093300
